# Contribution of VEGF-B-Induced Endocardial Endothelial Cell Lineage in Physiological Versus Pathological Cardiac Hypertrophy

**DOI:** 10.1161/CIRCRESAHA.123.324136

**Published:** 2024-04-24

**Authors:** Ibrahim Sultan, Markus Ramste, Pim Peletier, Karthik Amudhala Hemanthakumar, Deepak Ramanujam, Annakaisa Tirronen, Ylva von Wright, Salli Antila, Pipsa Saharinen, Lauri Eklund, Eero Mervaala, Seppo Ylä-Herttuala, Stefan Engelhardt, Riikka Kivelä, Kari Alitalo

**Affiliations:** 1 Wihuri Research Institute (I.S., M.R., P.P., K.A.H., Y.v.W., S.A., P.S., R.K., K.A.), Faculty of Medicine, Biomedicum Helsinki, University of Helsinki, Finland.; 2 Translational Cancer Medicine Program (I.S., M.R., P.P., K.A.H., Y.v.W., S.A., P.S., K.A.), Faculty of Medicine, Biomedicum Helsinki, University of Helsinki, Finland.; 3 Institute of Pharmacology and Toxicology, Technical University of Munich, DZHK partner site Munich Heart Alliance, Germany (D.R., S.E.).; 4 A.I. Virtanen Institute, University of Eastern Finland, Kuopio, Finland (A.T., S.Y.-H.).; 5 Oulu Center for Cell-Matrix Research, Faculty of Biochemistry and Molecular Medicine, Biocenter Oulu, University of Oulu, Finland (L.E.).; 6 Department of Pharmacology (E.M.), Faculty of Medicine, University of Helsinki, Finland.; 7 Stem Cells and Metabolism Research Program (R.K.), Faculty of Medicine, University of Helsinki, Finland.; 8 Faculty of Sport and Health Sciences, University of Jyväskylä, Finland (R.K.).; 9 RNATICS GmbH, Planegg, Germany (D.R.).

**Keywords:** angiogenesis, coronary vessels, heart failure, myocardial infarction, pregnancy

## Abstract

**BACKGROUND:**

Preclinical studies have shown the therapeutic potential of VEGF-B (vascular endothelial growth factor B) in revascularization of the ischemic myocardium, but the associated cardiac hypertrophy and adverse side effects remain a concern. To understand the importance of endothelial proliferation and migration for the beneficial versus adverse effects of VEGF-B in the heart, we explored the cardiac effects of autocrine versus paracrine VEGF-B expression in transgenic and gene-transduced mice.

**METHODS:**

We used single-cell RNA sequencing to compare cardiac endothelial gene expression in VEGF-B transgenic mouse models. Lineage tracing was used to identify the origin of a VEGF-B-induced novel endothelial cell population and adeno-associated virus–mediated gene delivery to compare the effects of VEGF-B isoforms. Cardiac function was investigated using echocardiography, magnetic resonance imaging, and micro-computed tomography.

**RESULTS:**

Unlike in physiological cardiac hypertrophy driven by a cardiomyocyte-specific VEGF-B transgene (myosin heavy chain alpha-VEGF-B), autocrine VEGF-B expression in cardiac endothelium (aP2 [adipocyte protein 2]-VEGF-B) was associated with septal defects and failure to increase perfused subendocardial capillaries postnatally. Paracrine VEGF-B led to robust proliferation and myocardial migration of a novel cardiac endothelial cell lineage (VEGF-B-induced endothelial cells) of endocardial origin, whereas autocrine VEGF-B increased proliferation of VEGF-B-induced endothelial cells but failed to promote their migration and efficient contribution to myocardial capillaries. The surviving aP2-VEGF-B offspring showed an altered ratio of secreted VEGF-B isoforms and developed massive pathological cardiac hypertrophy with a distinct cardiac vessel pattern. In the normal heart, we found a small VEGF-B-induced endothelial cell population that was only minimally expanded during myocardial infarction but not during physiological cardiac hypertrophy associated with mouse pregnancy.

**CONCLUSIONS:**

Paracrine and autocrine secretions of VEGF-B induce expansion of a specific endocardium-derived endothelial cell population with distinct angiogenic markers. However, autocrine VEGF-B signaling fails to promote VEGF-B-induced endothelial cell migration and contribution to myocardial capillaries, predisposing to septal defects and inducing a mismatch between angiogenesis and myocardial growth, which results in pathological cardiac hypertrophy.

**Novelty and Significance**

**What Is Known?**

- VEGF-B (vascular endothelial growth factor B) has greater translational potential than VEGF-A, which induces vascular leakage and tissue inflammation.
- VEGF-B gene transfer to the heart induces angiogenesis and physiological cardiac hypertrophy.
- VEGF-B has 2 isoforms, which have distinct biochemical and functional properties.

**What New Information Does This Article Contribute?**

- VEGF-B gene transfer to the heart leads to vascular expansion of a unique endocardium–derived endothelial cell (EC) population that is slightly expanded after myocardial infarction but not during pregnancy.
- VEGF-B production in cardiac ECs leads to cardiac septal defects, pathological angiogenesis, and cardiac hypertrophy.
- Posttranscriptional regulation of VEGF-B isoforms differs in cardiomyocytes versus cardiac ECs.

Induction of myocardial vessel growth could alleviate cardiomyocyte damage during myocardial ischemia. However, attempts to use VEGF-A for this purpose have been halted because it promotes vascular leakage and inflammation. In contrast, VEGF-B provides a promising therapeutic tool in the heart as it causes little vascular leakage or inflammation. Furthermore, high doses of VEGF-B are well tolerated because it functions indirectly via endogenous VEGF-A signaling. Our present study shows that VEGF-B production by cardiac ECs leads to cardiac pathology. Comparison of the vascular phenotypes in the 2 transgenic models revealed that VEGF-B overexpression induces a novel cardiac EC lineage (VEGF-B-induced endothelial cells) that contributes robustly to subendocardial vessels on paracrine VEGF-B expression but little to autocrine expression. In contrast, the VEGF-B-induced endothelial cells were not expanded in cardiac hypertrophy associated with mouse pregnancy, and they showed only slight expansion during myocardial infarction caused by coronary vessel ligation. The endocardium-derived VEGF-B-induced endothelial cells should provide a vascular marker for the targeting and monitoring of therapeutic attempts to salvage myocardial tissue in critical ischemia. Furthermore, our findings on the cell type-specific regulation of VEGF-B isoforms provide a valuable basis for further translational development of VEGF-B.

**Meet the First Author, see p 1401**

Ischemic heart disease is the number one cause of mortality worldwide.^1^ Cardiac ischemia induced by occlusion of a coronary vessel endangers a massive sudden loss of cardiomyocytes that exceed the regenerative capacity of the myocardium.^2^ Due to their angiogenic properties, VEGFs (vascular endothelial growth factors) and vascular endothelial growth factor receptors (VEGFRs) provide promising therapeutic tools for alleviation of cardiac ischemia before cardiomyocyte damage.^3^ Attempts to use VEGF-A gene transfer as a therapeutic tool to induce angiogenesis^4–8^ have been hindered by VEGF-A-induced vascular leakage and tissue inflammation.^9,10^ More recent studies using VEGF-B gene transduction have shown that VEGF-B activates the endogenous VEGF-A/VEGFR-2 signaling pathway activity,^11–14^ without resulting in significant tissue inflammation or vascular leakage.^12,15^

The *Vegfb* gene encodes 2 protein isoforms, VEGF-B_186_ and VEGF-B_167_, which are secreted by cardiomyocytes in the mammalian heart. Both isoforms bind to VEGFR-1, expressed on the surface of cardiac endothelial cells (ECs).^16^ After its secretion, VEGF-B_186_ undergoes proteolytic cleavage, resulting in VEGF-B_127_ isoform.^17^ Unlike VEGF-B_186_, VEGF-B_167_ and VEGF-B_127_ bind also to the NRP-1 (neuropilin-1) coreceptor.^17^ Previous studies have shown that a cardiomyocyte-specific myosin heavy chain alpha (αMHC)-VEGF-B transgene that produces both VEGF-B_186_ and VEGF-B_167_ isoforms expands the cardiac vasculature and leads to a physiological-like cardiac hypertrophy.^11–14^ Transgenic rats expressing the αMHC-VEGF-B transgene have normal cardiac function and show an improved cardiac ejection fraction (EF%) and fractional shortening (FS%) after myocardial infarction (MI) caused by ligation of the left anterior descending (LAD) coronary artery.^14^ Furthermore, expression of VEGF-B_186_ in mice via AAV (adeno-associated virus) vector delivery decreased scarring and improved cardiac perfusion after MI,^13^ and VEGF-B_167_ gene transfer provided protection against nonischemic heart failure in a canine cardiac tachypacing model.^18,19^ However, in one study, excessive levels of VEGF-B_186_, and in particular of its cleaved VEGF-B_127_ form, predisposed the hypertrophic heart to arrhythmias after MI or dobutamine treatment, which both increase ectopic ventricular activity.^20,21^

The metabolic effects of VEGF-B overexpression are of interest when considering its therapeutic potential in the setting of cardiovascular disease. In obese and insulin-resistant mice, VEGF-B gene transfer promoted weight loss and attenuated metabolic complications.^22^ In rats, VEGF-B also decreased coronary lipoprotein lipase activity and cardiac lipid metabolite accumulation and augmented cardiac insulin action, suggesting that it may be cardioprotective in diabetes.^23^ However, in long-term experiments, transgenic mice expressing only the human VEGF-B_167_ isoform in cardiomyocytes showed an increased death rate, apparently because of mitochondrial lipotoxicity.^24^

As these contrasting results indicate, further development of VEGF-B as a therapeutic tool requires knowledge of its optimal site of expression and associated adverse effects and of the properties of its 2 isoforms. In the present study, we report that paracrine VEGF-B signaling induces a novel endocardium-derived cardiac EC population that is marked by the expression of a unique set of transcripts. We have evaluated here this EC population in 2 different models of cardiac hypertrophy and signaling to better understand how VEGF-B can be safely used in the heart. EC proliferation is stimulated by the binding of VEGF-A to its receptor VEGFR-2. EC migration during sprouting angiogenesis occurs toward higher concentrations of VEGF-A in a gradient generated by VEGF-A-expressing cells.^25^ We wanted to establish a cardiac model of autocrine VEGF-B signaling that would stimulate EC proliferation but not directed migration. For this, we expressed the VEGF-B transgene as an autocrine ligand in the coronary endothelium, under the control of aP2 (adipocyte protein 2)/FABP4 (fatty acid binding protein 4) promoter,^22^ and compared its effects to those in the cardiomyocyte-specific paracrine model (αMHC-VEGF-B),^12^ with particular attention to coronary vessel growth and cardiac hypertrophy.

Our results indicate that autocrine VEGF-B signaling that lacks the paracrine VEGF-B gradient for EC migration induces a scattered pattern of novel VEGF-B-induced endothelial cells (VEGFB-iECs), which, however, fail to contribute to subendocardial angiogenesis as much as in the paracrine model. The autocrine VEGF-B signaling led to septal defects already during embryogenesis. The surviving transgenic mice enabled us to characterize the pathological angiogenesis in postnatal mice. We also studied this novel EC population in other models of cardiac hypertrophy and observed that it was not induced in cardiac hypertrophy during pregnancy and was only minimally expanded by MI. These results indicate that VEGF-B can enhance cardiac angiogenesis in normal and ischemic hearts in a unique way that should allow the development of safe VEGF-B-based therapies for patients with cardiac ischemia.

## METHODS

### Data Availability

All methods and study materials are available to other researchers upon reasonable request. All single-cell RNA sequencing data are available from the GEO database under accession number GSE261561.

See the detailed Methods section in the Supplemental material.

### Mouse Models

All animal experimental procedures were approved by the National Animal Experiment Board following the regulations of the European Union and Finnish legislation. The Supplemental Material contains detailed information on the transgenic rodent lines, experimental procedures, and treatments used in the study. The numbers of mice used in each experiment are indicated in the respective figure legends.

### Cardiac Echography, ECG, and Magnetic Resonance Imaging

Analysis of cardiac function was performed under isoflurane anesthesia using the Vevo 2100 Ultrasound system (FUJIFILM VisualSonics, Inc). Parameters listed in Tables S1 and S2 were calculated using the 2-dimensional M-mode. Lead II ECG signals were acquired using limb electrodes, and the processing of digital signals was performed using the previously published program in MATLAB (MathWorks, Natick, MA).^26^ Cardiac magnetic resonance imaging was performed as described earlier.^27^

### Ligation of the LAD Coronary Artery

Induction of cardiac ischemia and subsequent MI was achieved by ligating the LAD coronary artery in adult mice as described earlier.^13^

### Statistical Analysis

**Figure 1.**
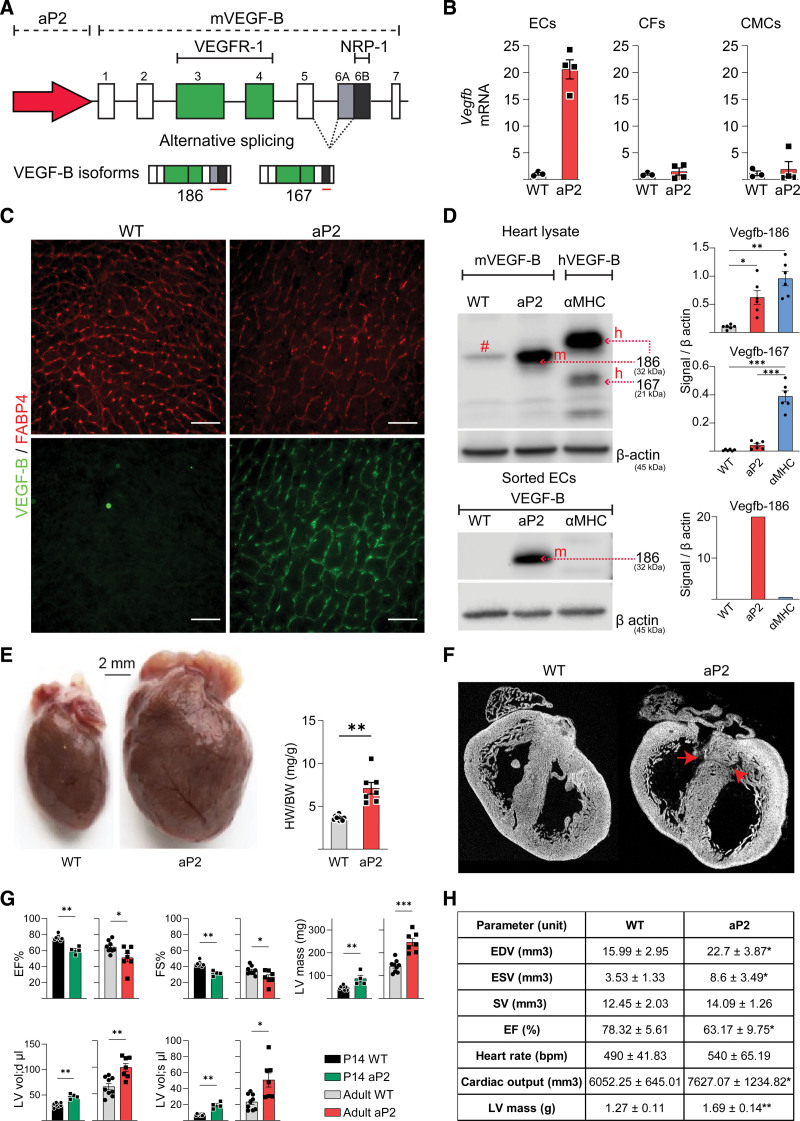
**Septal defects and massive pathological cardiac hypertrophy caused by autocrine VEGF-B (vascular endothelial growth factor B) expression in coronary endothelial cells (ECs). A**, Schematic illustration of the aP2 (adipocyte protein 2)-VEGF-B transgene encoding both VEGF-B isoforms (VEGF-B_186_ and VEGF-B_167_). **B**, Quantitative real-time polymerase chain reaction (RT-qPCR) analysis of m*Vegfb* RNA in ECs, cardiac fibroblasts (CFs), and cardiomyocytes (CMCs) isolated from hearts of 10-week-old aP2-mVEGF-B mice (n=3 wild type [WT], 4 aP2). *Unpaired Mann-Whitney *t* test. **C**, Representative immunohistochemical stainings of aP2 (FABP4 [fatty acid binding protein 4]) and VEGF-B in cryosections from the hearts of adult aP2-VEGF-B mice and their WT littermate mice. Scale bars, 50 µm. **D**, Western blot (WB) analysis of VEGF-B and β-actin in heart lysates (n=6) and isolated ECs from adult aP2-VEGF-B, myosin heavy chain alpha (αMHC)-VEGF-B and WT mice. Quantifications show the fold expression normalized to β-actin. # indicates a background band. *Brown-Forsythe and Welch ANOVA test with Dunnett correction. **E**, Macroscopic images showing the cardiac hypertrophy phenotype in aP2-VEGF-B mice and quantification of the heart weight (HW)/body weight (BW) ratio (n=9 WT, 7 aP2). Scale bar, 2 mm. *Unpaired 2-tailed *t* test with Welch correction. **F**, Ex vivo micro-computed tomography (μCT) scans of P0 aP2-VEGF-B and WT littermate hearts. Red arrows point to the septal defects. **G**, Echocardiography parameters from aP2-VEGF-B and WT littermate hearts at P14 (n=8 WT, 4 aP2) and at 10 wk (n=9 WT, 7 aP2). *P14 unpaired Mann-Whitney *t* test; adults unpaired 2-tailed *t* test with Welch correction. **H**, Cardiac magnetic resonance imaging (MRI) parameters from aP2-VEGF-B and WT littermate hearts (n=5). Values are represented as means±SD. *Unpaired Mann-Whitney *t* test. EDV indicates end-diastolic volume; EF, ejection fraction; ESV, end-systolic volume; LV, left ventricle; and SV, stroke volume.

Prism 10.2.0 software was used for statistical analyses (GraphPad Software, San Diego, CA). In the case of testing a hypothesis, we did not apply experiment-wide multiple test correction. In experiments where the (n) number was ≥6 per group, we tested for normality using the Shapiro-Wilk test. If the data passed normality testing, we applied parametric tests. For the comparison of 2 groups, we used *t* test with Welch correction, while a comparison of ≥3 groups was performed using 1- or 2-way parametric ANOVA tests. In conditions where data did not pass normality testing for n ≥6 or where the (n) number per group was below 6, we applied nonparametric tests. For the comparison of 2 groups, we used the Mann-Whitney *t* test, while a comparison of 3 groups or more was performed using the Kruskal-Wallis ANOVA test. In echocardiography testing where repeated measurements have been taken from the same animals, we tested for normality using the Shapiro-Wilk test despite having (n) number below 6 as the data are supported to follow normal distribution based on data published earlier.^13,28–30^ If the data passed normality testing, we applied parametric 2-way ANOVA testing, while values that did not pass normality testing were analyzed using Wilcoxon matched-pairs signed rank test using the Holm-Sidak method. Based on the observed biological outcome, post hoc correction tests were applied/not applied to further pinpoint which specific means are significant/nonsignificant from the others. Values are indicated as mean±SEM in all figures and as mean±SD in tables (Tables S1 and S2; Figure 1H; Figure S2H). All mouse (n) numbers and statistical tests used are indicated in figure legends, where the absence of an asterisk indicates nonsignificance, * indicates *P*≤0.05, ** indicates *P*≤0.01, and *** indicates *P*≤0.001. All exact *P* values and statistical details, such as normalization procedures, tests establishing normality, sample sizes, named statistical tests, named post hoc correction, and raw/corrected *P* values, are detailed in Table S3.

## RESULTS

### Production and Analysis of Transgenic Mice Expressing VEGF-B in Coronary Endothelium

Endogenous cardiac EC proliferation and migration processes are driven predominantly by paracrine signaling via VEGF-A.^3,31^ We aimed to create a model that short-circuits the signals for EC migration along the paracrine growth factor gradient and retains the signals for EC proliferation. Accordingly, we used aP2-VEGF-B transgenic mice that express the mouse VEGF-B gene under the aP2/FABP4 promoter (Figure 1A),^22^ which is activated in coronary ECs starting at embryonic day E14.5.^32^ At this timepoint in wild-type (WT) mice, the FABP4-expressing cells arise by angiogenic sprouting from the first wave of ECs that colonize the heart and later form the coronary vasculature. During further development, all cardiac blood vascular ECs start to express aP2/FABP4.^32^ As shown by the comparison of VEGF-B RNA expression between WT and transgenic mice in Figure 1B, we detected highly elevated levels of VEGF-B mRNA in the coronary ECs isolated by fluorescence-activated cell sorting from 10-week-old aP2-VEGF-B mice (Figure S1) but not in cardiomyocytes or cardiac fibroblasts isolated from the same mice. VEGF-B immunostaining of cardiac tissue sections confirmed specific expression of the transgene in cardiac ECs (Figure 1C). Western blot analysis of the cardiac lysates and isolated cardiac ECs from the aP2-VEGF-B mice showed expression of the VEGF-B_186_ isoform but, surprisingly, no expression of VEGF-B_167_ (Figure 1D). The mouse VEGF-B_186_ isoform was detected also in sera from the transgenic mice at an average concentration of 10 to 15 ng/mL (Figure S2A), whereas VEGF-B concentration in sera from WT mice was below the enzyme-linked immunosorbent assay detection level.

At 10 weeks of age, the aP2-VEGF-B transgenic hearts were considerably bigger than the hearts of their WT littermates, as indicated by the heart weight to body weight ratios (Figure 1E). However, there was no significant difference in the body weights or the weights of epididymal or subcutaneous fat depots, spleens, kidneys, or lungs between the aP2-VEGF-B mice and their WT littermates (Figure S2B). Immunohistochemical analysis confirmed that the aP2-VEGF-B mice have larger cardiomyocytes and a greater myocardial vessel area fraction than their WT littermates (Figure S2C and S2D).

### Development of Cardiac Pathology in the aP2-VEGF-B Mice

On genotyping of the offspring from matings between the aP2-VEGF-B heterozygous and WT mice at 4 weeks of age, we observed that only about 15% to 20% of the mice were aP2-VEGF-B transgenic, instead of the expected 50%. In contrast, at embryonic day 18.5 (E18.5), both genotypes were equally represented (Figure S2E). Peripartum observation of the pups indicated that about half of the transgenic pups die or are eaten by the mothers during the first hours after birth. This differed significantly from the αMHC-VEGF-B mice that express the transgene in cardiomyocytes and are born in normal Mendelian ratios (Figure S2E). Figure S2F shows a comparison of cardiac hypertrophy development in the aP2-VEGF-B and αMHC-VEGF-B pups at P0, P7, P14, and P28. As can be seen from the data, at P0, there is no significant difference in heart weight/body weight ratios between the 2, but both the αMHC-VEGF-B mice^13^ and the aP2-VEGF-B mice show clear cardiac hypertrophy already at P7 (Figure S2F). We then used micro-computed tomography imaging to compare the transgenic hearts of pups that died postnatally at P0 to the hearts of their WT littermates. Intriguingly, we observed cardiac ventricular septal defects of varying severity in the transgenic pups (Figure 1F; Figure S3A and S3B). Furthermore, echocardiography demonstrated an impaired cardiac function in the surviving transgenic pups at P14. We also found decreased left ventricular EF% in the adult transgenic mice (Figure 1G), which was confirmed by magnetic resonance imaging (Figure 1H; Figure S2G). ECG^26^ of the adult transgenic mice showed a prolonged PQ-interval and increased amplitude and widening of the QRS complex (Figure S2H). These findings indicated that the autocrine VEGF-B signaling in the developing heart leads to septal defects and in the surviving transgenic mice to cardiac hypertrophy with left ventricular dysfunction, thus providing a model of pathological cardiac hypertrophy.

### VEGF-B Transgenic Mice Amplify a Unique Cardiac EC Population

**Figure 2.**
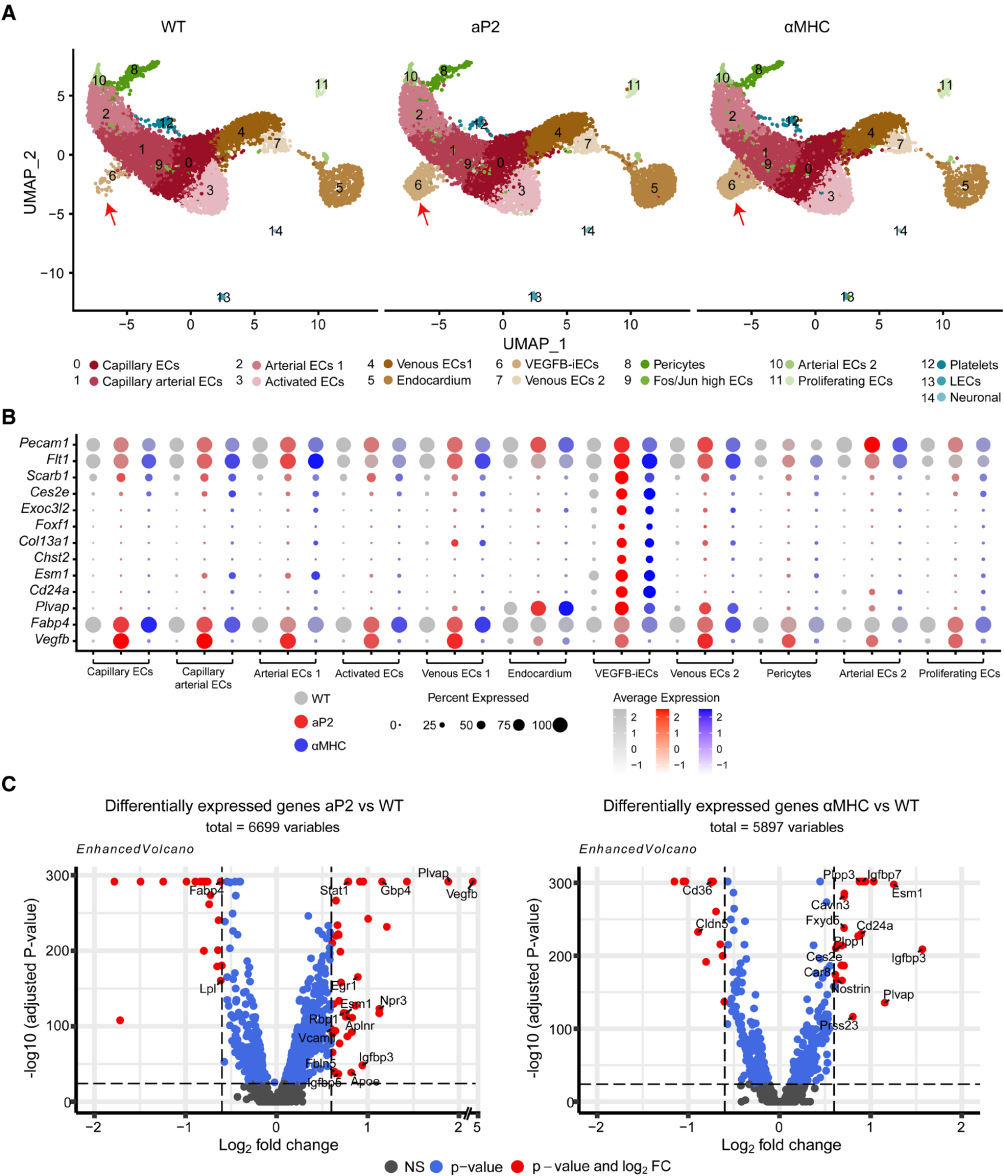
**Single-cell RNA sequencing of isolated cardiac endothelial cells (ECs) from VEGF-B (vascular endothelial growth factor B) transgenic (TG) mice reveals the expansion of a unique EC population. A**, Uniform manifold approximation and projection (UMAP) plot showing clusters obtained from Seurat-integrated analysis of cardiac ECs isolated from aP2 (adipocyte protein 2)-VEGF-B, myosin heavy chain alpha (αMHC)-VEGF-B, and wild-type (WT) adult mice. **B**, Dot plot showing the average expression and the expression percentage of vascular endothelial growth factor B–induced endothelial cells (VEGFB-iECs) and EC markers in selected clusters of aP2-VEGF-B, αMHC-VEGF-B, and WT mice. The dot plot was generated using the DotPlot() function embedded in the Seurat package. The average expression was plotted using log-normalized and scaled data stored in the Seurat object under the slot scale.data. **C**, Volcano plots showing the highest differentially expressed genes (DEGs) across all cardiac ECs from aP2-VEGF-B vs WT mice or αMHC-VEGF-B vs WT mice. Volcano plots present the values obtained from Seurat-integrated DEG analysis where avg log2FC is presented on the *x* axis and log10-adjusted *P* value is presented on the *y* axis. The *P* value is adjusted based on Bonferroni correction using all features in the data set. The single-cell data sets were generated through the pooling of 3 hearts into 1 sample per group.

To analyze cellular mechanisms that contribute to the physiological (αMHC-VEGF-B) and pathological (aP2-VEGF-B) cardiac hypertrophy, we subjected the cardiac ECs to single-cell RNA sequencing (scRNA-seq) analysis. Isolated cardiac ECs of adult WT, aP2-VEGF-B, and αMHC-VEGF-B mice yielded several cell types that were grouped into 15 different clusters based on their exclusive expression of marker genes (Figure 2A; Figure S4A). Interestingly, a comparison of the clusters between the WT and transgenic mice identified a cell population (cluster 6) that was significantly increased in both transgenic models (Figure 2A; Figure S4B). This EC population showed high expression of a distinct set of transcripts (*Plvap*, *Cd24a*, *Chst2*, *Exoc3l2*, *Col13a1*, *Ces2e*, *Esm1*, and *Foxf1*), of which all except *Plvap* were exclusively expressed in the cluster 6 EC population (Figure 2B; Figure S4C). Thus, we designated these cells as VEGF-B-induced ECs (hereafter VEGFB-iECs). Further analysis showed that the VEGFB-iEC markers, *Plvap*, *Cd24a*, *Esm1*, and *Ces2e*, represent some of the most differentially expressed genes among all cardiac ECs isolated from the transgenic versus WT mice (Figure 2C). These findings indicated that significant VEGF-B-specific transcriptomic changes occur in the cardiac EC population in the 2 transgenic models that express VEGF-B in the heart. We also performed scRNA-seq of the adult cardiac stromovascular fraction from the aP2-VEGF-B, αMHC-VEGF-B, and WT hearts to address possible VEGF-B-induced effects in other cardiac cell types. Analysis of the scRNA-seq data showed 23 cell clusters that were annotated based on their expression of specific markers (Figure S5A and S5B). These included cardiac ECs, cardiac fibroblasts, and immune cells. We also captured a small population that likely represents skewed small cardiomyocytes that managed to fit within the beads used in the scRNA pipeline. Analysis of cell numbers/clusters did not indicate major differences between the WT and VEGF-B transgenic mice, apart from a slightly increased number of captured macrophages in the VEGF-B transgenic mice (Figure S5C). Also, ECs in the stromovascular fraction cell analysis showed increased expression of VEGFB-iEC marker transcripts in aP2-VEGF-B mice and even more prominently in αMHC-VEGF-B mice (Figure S5D). Analysis of differential gene expression in cardiac fibroblasts in the transgenic versus WT mice did not indicate major differences, with the exception that low levels of *Vegfb* mRNA were detected in cardiac fibroblasts in aP2-VEGF-B mice (Figure S5E).

Because scRNA-seq analysis suggested that there is an increased number of macrophages in the VEGF-B transgenic versus WT hearts, we performed immunofluorescence analysis of Cd45 leukocyte and Cd206 M2 macrophage markers in cardiac sections, which, however, did not indicate any significant differences between the VEGF-B transgenic mice or their WT littermates (Figure S6A and S6B). For further confirmation, we used quantitative real-time polymerase chain reaction (RT-qPCR) to check the cardiac expression of *Adgre1* (F4/80), a well-known and widely used marker of murine macrophage populations, and the inflammation markers *Vcam1*, *Il6*, *Il1*-β, and *Tnfα* (Figure S6C). We also analyzed the cardiac stress markers *Nppa* and *Nppb* (Figure S6D) but found no significant differences in these transcripts between the transgenic and WT mice.

**Figure 3.**
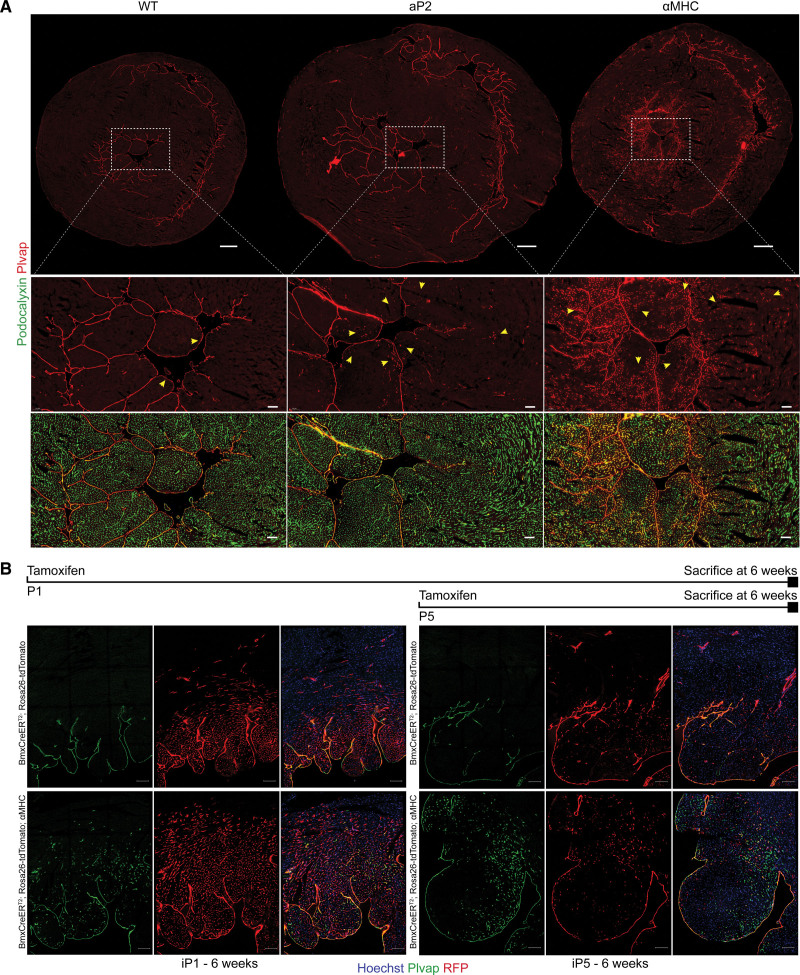
**Vascular endothelial growth factor B–induced endothelial cells (VEGFB-iECs) are derived from the endocardium and reside in the subendocardium. A**, Representative immunohistochemical stainings of Plvap and podocalyxin in cardiac cryosections from aP2 (adipocyte protein 2)-VEGF-B, myosin heavy chain alpha (αMHC)-VEGF-B, and wild-type (WT) mice. Scale bars, 500 and 100 µm, respectively. Note that the yellow arrows point to VEGFB-iECs. **B**, Representative immunohistochemical stainings of RFP (red fluorescent protein) and Plvap in 200-µm-thick cardiac sections from 6-week-old BmxCreER^T2^; Rosa26-tdTomato; αMHC-VEGF-B and their BmxCreER^T2^; and Rosa26-tdTomato littermates that were induced with tamoxifen on P1 or P5. Scale bar, 100 µm.

Immunohistochemical staining of cardiac sections confirmed that Plvap marks only the endocardium in WT mice^33^ (Figure 3A). Staining of sections from aP2-VEGF-B hearts revealed a similar pattern, plus a weaker spotwise staining in a limited number of subendocardial vessels (Figure 3A, quantified in Figure S7B). Interestingly, the αMHC-VEGF-B mice showed an additional striking pattern of expression, in a gradient-like fashion with the strongest staining in capillary-sized vessels in the immediate subendocardial area, gradually fading toward the mid-myocardial and epicardial areas (Figure 3A). To accurately localize the expanded EC population in the heart, we stained thick cardiac sections from phosphate-buffered saline–perfused hearts with antibodies against the protein encoded by the *Cd24* marker. In the WT hearts, weak Cd24 staining decorated 5% to 10% of the podocalyxin-positive endothelium in different regions of the heart (Figure S7A and S7B). In the aP2-VEGF-B mice, both subendocardial and subepicardial regions showed increased Cd24 staining (in 25 and 15% of the ECs, respectively), while, in the αMHC-VEGF-B mice, Cd24 staining was observed mostly in the subendocardial region (26% of the ECs; Figure S7A and S7B). In addition to the staining of Cd24 in the ECs, we observed occasional Cd24-positive cells within the vascular lumen. Because Cd24 is expressed in a majority of immature cells of most if not all major hematopoietic lineages,^34,35^ these cells had likely escaped vascular phosphate–buffered saline perfusion. Quantifications across different regions of the heart showed a consistently significant increase in vessel lumen size in aP2-VEGF-B mice, whereas the αMHC-VEGF-B hearts showed a trend of increasing lumen size only in the mid-myocardium (Figure S7C). For additional confirmation of the location of the VEGFB-iECs, we costained Col13a1 and Plvap in cardiac sections from WT and VEGF-B transgenic mice. We observed a clear overlap of both markers in the subendocardial ECs (Figure S8A). Overall, the superimposed expression patterns of Plvap, Cd24, and Col13a1 markers suggested that the coronary endothelium responds to VEGF-B by activation of ECs first in the subendocardial region and then by a more widespread activation in the coronary vasculature.

We next validated that the expanded VEGFB-iEC-containing vessels are functional. We injected fluorescent LE-lectin via the tail vein into αMHC-VEGF-B and WT littermate mice, allowed it to circulate for 10 minutes, and then euthanized the mice and stained cardiac sections for Plvap. Decoration of the cardiac Plvap-positive endothelium with lectin fluorescence confirmed that the VEGFB-iEC vessels are functional (Figure S8B).

To analyze the endothelial identity of VEGFB-iECs, we checked the expression of capillary, arterial, and venous markers in them by using our FAC-sorted EC scRNA-seq data set. We found increased expression of the capillary marker *Car4* and minimal expression of arterial marker *Gja4* or venous marker *Nr2f2* (nuclear receptor subfamily 2 group F member 2; Figure S9A). We further confirmed the capillary endothelial identity by immunofluorescence staining and found that the Plvap-positive ECs were negative for the venous marker Nr2f2 (also known as Coup-tfII [chicken ovalbumin upstream promoter transcription factor II]), and unlike arterial ECs, they had no adjacent SMA (smooth muscle actin)–positive smooth muscle cells (Figure S9B). The VEGFB-iECs can, thus, be classified as capillary ECs.

To explore whether the VEGFB-iEC markers are cardiac-specific and to compare their expression between the aP2-VEGF-B and αMHC-VEGF-B mice, we used RT-qPCR to quantify the marker transcripts in the heart, lungs, skeletal muscles (tibialis anterior), and livers from the transgenic and WT control mice (Figure S9C). We found that the VEGFB-iEC marker transcripts are more abundant in aP2-VEGF-B than WT hearts and most significantly abundant in the αMHC-VEGF-B hearts (Figure S9C). Presumably, because the aP2 promoter that drives transgenic expression is weakly active also in skeletal muscle,^36^ 6 of the 8 transcript markers were more elevated in the tibialis anterior muscle in the aP2-VEGF-B mice than in the WT mice (Figure S9C). Thus, our results show that VEGF-B induces expansion of capillary-like ECs that exhibit specific markers, and that paracrine VEGF-B signaling is a stronger inducer of the VEGFB-iECs than autocrine VEGF-B signaling.

### Cardiac VEGFB-iECs Are Derived From the Endocardium

In the heart, the expression of Plvap is restricted to the endocardium in WT mice^33^ (Figure 3A). Furthermore, our scRNA-seq analysis and immunohistochemical staining of Plvap plus SMA in the transgenic mice indicated that the arterial and VEGFB-iEC markers are mutually exclusive (Figure S9A and S9B). This suggested that VEGFB-iECs are endocardium-derived. To lineage trace the endocardial cells, we crossed αMHC-VEGF-B mice with BmxCreER^T2^; Rosa26-LSL-tdTomato mice that express the inducible recombinase in endocardial ECs and in a subset of arterial ECs. We then activated the lineage tracer by tamoxifen in BmxCreER^T2^; Rosa26-tdTomato; αMHC-VEGF-B mice and their BmxCreER^T2^; Rosa26-tdTomato littermates at postnatal day 1 (P1) or at postnatal day 5 (P5) and analyzed the mice at 6 weeks of age (Figure 3B). Immunofluorescence analysis of cardiac sections from BmxCreER^T2^; Rosa26-tdTomato; αMHC-VEGF-B mice showed a vascular pattern that indicated expansion of the tdTomato+ (RFP [red fluorescent protein] +) endothelium, most extensively in the subendocardial vessels, with a descending gradient toward the epicardial side of the left ventricular wall (Figure 3B), whereas the BmxCreER^T2^; Rosa26-tdTomato littermates showed a comparable gradient in the immediate subendocardial region that did not extend deeper into the myocardium (Figure 3B). Apart from the endocardial cells and few other scattered ECs, we did not observe any other Plvap-positive ECs in WT hearts. However, when lineage tracing was activated in BmxCreER^T2^; Rosa26-tdTomato; αMHC-VEGF-B pups at P1, these mice showed 87.62% overlap between tdTomato (RFP) and Plvap staining at 6 weeks. Induction at P5 resulted in 40.07% overlap at 6 weeks, indicating that the VEGFB-iECs are induced during the early postnatal period (Figure 3B). Because recent studies have shown that a subset of coronary arteries forms by angiogenic extension of endocardium-derived Dll4-positive vascular tunnels in the neonatal heart,^37,38^ we stained also Dll4 in adult aP2-VEGF-B, αMHC-VEGF-B, and WT mice. We found Dll4 in arteries coated by SMA-positive smooth muscle cells, whereas the VEGFB-iECs, identified by Plvap staining, did not show Dll4 staining (Figure S10A).

We were intrigued by our result showing the early postnatal endocardial contribution to VEGFB-iECs, as the endocardium is believed to not express VEGFR-2,^39^ while our mechanistic hypothesis was that the VEGFB-iECs are induced by displacement of the endogenous VEGF-A from VEGFR-1 to VEGFR-2. We, thus, stained the endocardium for VEGFR-2 and Cd31 in P1 and in adult hearts. We detected weak VEGFR-2 staining only in some segments of the endocardial layer at P1 (Figure S10B). Cardiac scRNA-seq data of FAC-sorted ECs from adult mice showed substantial expression of VEGFR-2 (*Kdr*) in the adult endocardium and elevated expression in VEGFB-iECs (Figure S10C). Immunostaining of adult WT hearts showed weak VEGFR-2-positive endocardial segments that were mostly located within the endocardium in between juxtaposed trabeculations that may experience only moderate fluid shear stress from the ventricular pumping of circulating blood (Figure S10D).

To test the function of VEGFR-2 in the induction of VEGFB-iECs, we generated BmxCreER^T2^; Rosa26-tdTomato; αMHC-VEGF-B; VEGFR-2^fl/fl^ mice and their BmxCreER^T2^; Rosa26-tdTomato; αMHC-VEGF-B; VEGFR-2^wt/wt^ littermates, deleted VEGFR-2 in the endocardium by administering tamoxifen daily during P1 to P3 and analyzed the mice at 4 to 6 weeks of age (Figure S11A). In contrast to the control αMHC-VEGF-B mice (Figure 3B; Figure S11A), the VEGFR-2-deleted αMHC-VEGF-B mice showed few Plvap-positive but tdTomato (RFP)-negative VEGFB-iECs in the subendocardial region (Figure S11A). Lineage tracing showed 87.62% overlap between RFP and Plvap staining in 6-week-old mice induced at P1 (Figure 3B), indicating that only few (≃12%) VEGFB-iECs were induced before P1. Such cells could account for the VEGFB-iECs observed in the αMHC-VEGF-B mice in which VEGFR-2 had been deleted. Furthermore, the tdTomato-positive ECs in the undeleted hearts extended deeper into the myocardium than in the VEGFR-2-deleted hearts (Figure S11A), indicating that endocardial VEGFR-2 deletion decreases the endocardial contribution to the expanding coronary vasculature. These results support the model that the VEGF-B transgene-induced activation of the endocardium during the early postnatal stages drives the formation of VEGFB-iECs that contribute to the perfusion of the subendocardial myocardium.

### VEGFB-iEC Population Does Not Expand During Physiological Cardiac Hypertrophy in Pregnant Mice

**Figure 4.**
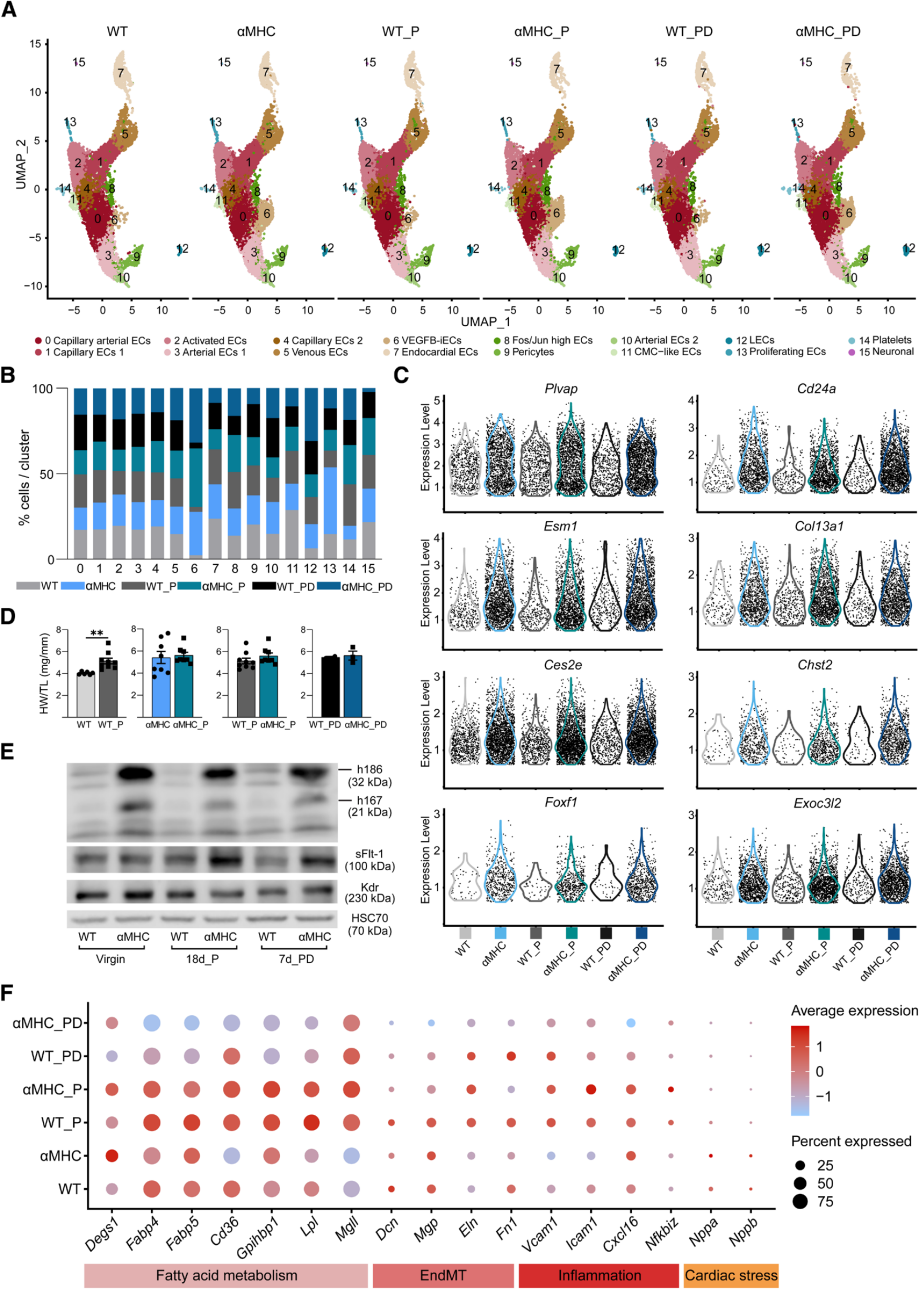
**Cardiac hypertrophy in pregnant mice does not increase vascular endothelial growth factor B–induced endothelial cells (VEGFB-iECs). A**, Uniform manifold approximation and projection (UMAP) plot showing clusters obtained from Seurat-integrated analysis of isolated cardiac endothelial cells (ECs) from pregnant wild-type (WT) and myosin heavy chain alpha (αMHC)-VEGF-B littermate mice at 18 days of pregnancy (_P) and 7 days postdelivery (_PD) in comparison to nonpregnant WT and αMHC-VEGF-B mice. **B**, Bar plot showing the comparison of the percentage of each cell population across all 6 groups. **C**, Violin plots showing expression of VEGFB-iEC cluster markers in all 6 groups. **D**, Heart weight (HW)/tibial length (TL) comparison in 18-day pregnant vs nonpregnant mice and at 7 days postdelivery from WT and αMHC-VEGF-B transgenic (TG) mice (n=6 WT, 9 WT_P, 8 αMHC, 8 αMHC_P, 2 WT_PD, and 3 αMHC_PD). *n≥6 unpaired 2-tailed *t* test with Welch correction; n≤6 unpaired Mann-Whitney *t* test. **E**, Western blot (WB) analysis of hVEGF-B, mVEGFR-1, and mVEGFR-2 in heart lysates from all 6 groups. **F**, Dot plot showing the average expression and the expression percentages of markers of fatty acid metabolism, endothelial-to-mesenchymal transition (EndMT), inflammation, and cardiac stress across all groups. The dot plot was generated using the DotPlot() function embedded in the Seurat package. The average expression is plotted using log-normalized and scaled data stored in the Seurat object under the slot scale.data. The single-cell data sets were generated through pooling of 2-3 hearts into 1 sample per group.

Normal pregnancy in mice involves a marked increase in blood volume and cardiac output that is associated with the growth of heart size by day 18 postcoitum and reversion of cardiac hypertrophy by day 7 postdelivery.^40^ To know whether the expansion of VEGFB-iECs was triggered during endogenous physiological cardiac hypertrophy, we isolated cardiac ECs from αMHC-VEGF-B mice and WT littermates at day 18 of pregnancy and 7 days postdelivery and subjected them to scRNA-seq analysis. At these timepoints, we recovered more VEGFB-iECs from the αMHC-VEGF-B mice than their WT littermates, but the results indicated that pregnancy as such did not alter the VEGFB-iECs (Figure 4A). Analysis of the number of cells per cluster and EC transcript expression confirmed that VEGFB-iECs were not amplified by pregnancy or after delivery (Figure 4B and 4C). Furthermore, heart weight/tibial length analysis confirmed cardiac hypertrophy at day 18 of pregnancy in WT mice and showed that VEGF-B transgene expression does not further increase the physiological cardiac hypertrophy in the pregnant mice (Figure 4D). Western blot analysis showed decreased expression of the VEGF-B transgene-encoded protein by day 18 of pregnancy and 7 days postdelivery and a parallel increase in the soluble form of VEGFR-1 (Figure 4E). In agreement with published data,^41^ transcripts related to fatty acid metabolism were upregulated on day 18 of pregnancy in both αMHC-VEGF-B transgenic and their WT littermate hearts (Figure 4F). These hearts expressed also low levels of markers of endothelial-to-mesenchymal transition, inflammation, and cardiac stress (Figure 4F).

### The VEGF-B-Induced Cardiac EC Population Is Slightly Expanded in Mice Subjected to MI

**Figure 5.**
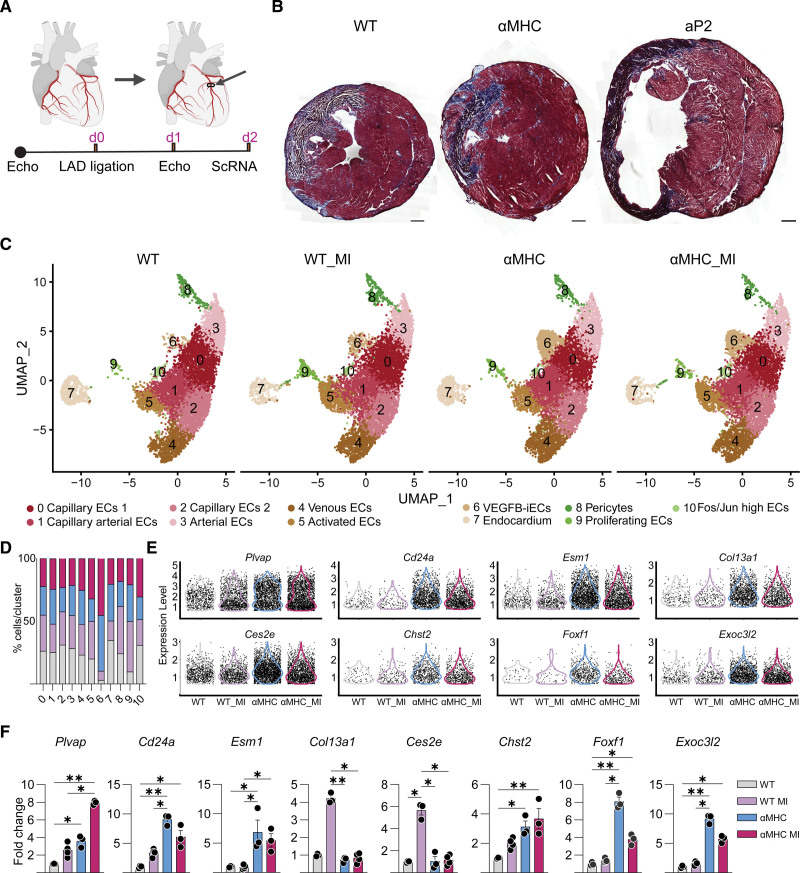
**Vascular endothelial growth factor B–induced endothelial cells (VEGFB-iECs) are not increased by myocardial infarction (MI) in transgenic (TG) mice. A**, Diagram indicating the left anterior descending (LAD coronary artery) ligation site and the timeline of the experiments. **B**, Representative images of Masson trichrome stained cardiac sections obtained from adult aP2 (adipocyte protein 2)-VEGF-B, myosin heavy chain alpha (αMHC)-VEGF-B, and wild-type (WT) mice. Scale bars, 500 µm. **C**, Uniform manifold approximation and projection (UMAP) plot showing clusters obtained from Seurat-integrated analysis of cardiac endothelial cells (ECs) isolated from WT and αMHC-VEGF-B mice under normal conditions and 2 days after LAD coronary artery ligation. **D**, Bar plot showing the comparison of the percentage of each cell population across all 4 groups. **E**, Violin plots showing the expression of VEGFB-iEC markers in all 4 groups. **F**, Quantitative real-time polymerase chain reaction (RT-qPCR) data showing relative expression of VEGFB-iEC marker transcripts in heart lysates (n=3–4). *Kruskal-Wallis ANOVA test. The single-cell data sets were generated through pooling of 2 to 3 hearts into 1 sample per group.

Li et al^42^ have shown that *Plvap* transcripts are induced in ECs during MI caused by ligation of the LAD coronary artery. Thus, Plvap provides an endothelial marker of cardiac neovasculogenesis.^42^ To investigate whether the MI-induced ECs reported by Li et al are related to the VEGFB-iECs, we ligated the LAD coronary artery in αMHC-VEGF-B mice and their WT littermates and analyzed the cardiac ECs by scRNA-seq 2 days after ligation (Figure 5A). Of interest, whereas most of the control and αMHC-VEGF-B mice survived the LAD operation, 9 of the 11 operated aP2-VEGF-B mice died within 1 day after the operation; thus, they were not analyzed further (Figure S12A).

Masson trichrome staining revealed significant thinning of the left ventricular wall in the surviving aP2-VEGF-B mice 2 days after the LAD operation (Figure 5B). In echocardiography, EF% and FS% were significantly decreased in both WT and αMHC-VEGF-B mice after the LAD operation (Table S1). Single-cell RNA sequencing comparison of all 4 groups indicated that the VEGFB-iECs were expanded by 4.3% by MI in the WT mice but not further increased in the αMHC-VEGF-B mice (Figure 5C and 5D). The slight increase in VEGFB-iECs cell numbers in the LAD-operated WT mice was not reflected as a significant increase in the total level of the VEGFB-iEC marker transcripts in the heart (Figure 5E). In addition, no further increase in VEGFB-iEC marker transcripts occurred in the LAD-operated versus nonoperated αMHC-VEGF-B mice (Figure 5E). In agreement with the previously published data,^42^ we observed that the MI treatment induced an overall increase of *Plvap* transcripts in cardiac ECs, and the elevated Plvap expression was further increased in transgenic mice subjected to MI (Figure 5E). To further validate our findings, we performed RT-qPCR for VEGFB-iEC marker transcripts using cardiac lysates from all 4 groups, which confirmed findings from the scRNA-seq analysis (Figure 5F). Taken together, the results indicate that the VEGFB-iEC population is not expanded during pregnancy and is only slightly expanded after MI. These findings suggest that the VEGFB-iECs possess a translational potential that can be used via gene transduction of VEGF-B in the ischemic heart.

### VEGF-B Isoforms Are Differentially Regulated at Posttranscriptional Level

As shown in Figure 1D, the αMHC-VEGF-B transgenic mice express both VEGF-B polypeptide isoforms in cardiomyocytes, whereas the aP2-VEGF-B mice express only the VEGF-B_186_ isoform in the cardiac ECs. Yet, RT-qPCR analysis indicated that the aP2-VEGF-B mice express similar overall levels of the 186 and 167 transcripts in the cardiac ECs (Figure S12B and S12C). This raised a question about the mechanism behind the differential expression of the VEGF-B isoform–encoded proteins in the cardiomyocytes versus ECs of transgenic mice. To answer this question, we compared the VEGF-B RNA versus protein expression in mice injected with AAV9s encoding VEGF-B_167_, VEGF-B_186_, or their combination. We chose the AAV9 vector because it transduces cardiac cells effectively.^43^ After 1 week of vector transduction, the VEGF-B polypeptides were analyzed from the blood plasma of mice treated with heparin to increase the release of the tissue-sequestered VEGF-B_167_ into blood. The results indicated that only the full-length 186 isoform and its proteolytically processed 127 aa fragment are produced by AAV9-VEGF-B_186_, whereas no signal was obtained from parallelly treated mice expressing AAV9-VEGF-B_167_, unless a 3-fold higher concentration of AAV9-VEGF-B_167_ was used (Figure S12D). We, furthermore, confirmed that the VEGF-B antibody recognizes both isoforms equally (Figure S12E).

We then analyzed whether the variation in AAV9 transduction or transgene RNA expression can explain the differences between the VEGF-B isoform levels. We first estimated the concentration of intraperitoneal transfected AAV in different tissues using qPCR amplification of the nontranscribed woodchuck hepatitis virus posttranscriptional regulatory element in the vector. The highest AAV9 transduction levels were detected in the spleen, heart, subcutaneous white adipose tissue, brown adipose tissue, and liver (Figure S12F). Analysis of transcripts encoding the 2 VEGF-B isoforms in the liver and heart at 1, 2, and 4 weeks after vector transduction indicated that there is a direct correlation between the transcript expression level and the delivered AAV9 dose (Figure S12G). Yet, on the protein level, the VEGF-B_186_ isoform was expressed at a much higher level than the VEGF-B_167_ isoform in the heart at all 3 timepoints (Figure S12H). This suggested that VEGF-B_167_ was degraded at a faster rate than VEGF-B_186_, regardless of the cell of origin.

All mice treated with the same dose of the AAV9-VEGF-B vector showed similar kinetics of cardiac EC proliferation that peaked at the 2-week timepoint, declining thereafter (Figure S13A and S13B). Interestingly, however, the mice transduced with AAV9-VEGF-B_167_ showed a trend of less cardiac EC proliferation than VEGF-B_186_ mice (Figure S13A and S13B), suggesting a faster turnover of the VEGF-B_167_ isoform. Mice injected with either vector showed cardiac hypertrophy at 2 and 4 weeks after gene delivery (Figure S13C), yet echocardiography did not indicate significant differences in EF% or FS% between the groups (Figure S13D; Table S2). To investigate whether the 2 VEGF-B isoforms induce different downstream signaling cascades, we injected WT mice with 19.8*10^^11^vp of AAV9 encoding either VEGF-B_186_, VEGF-B_167_, or scrambled control. Two weeks later, we analyzed VEGFR-1, VEGFR-2, and NRP-1 and the phosphorylation of VEGFR-1, ERK1/2, and AKT by gel electrophoresis of cardiac lysates. We found no significant difference in the phosphorylation of VEGFR-1 between the different groups (Figure S14A and S14B). However, sVEGFR-1 (soluble VEGFR-1) and the premature form of VEGFR-2^44^ were significantly elevated in the AAV9-VEGF-B_186_ and AAV9-VEGF-B_167_ hearts than in control hearts. In line with our previous findings, the mature form of VEGFR-2^44^ showed elevated expression in mice treated with AAV9-VEGF-B_186_ (Figure S14A and S14B). NRP-1 and VEGFR-1 were significantly increased in the AAV9-VEGF-B_186_-treated mice in comparison to the 2 other groups, perhaps because of the more abundant expression of VEGF-B_186_ than VEGF-B_167_ (Figure S14A and S14B). Probing of VEGFR-2 downstream signaling by phospho-ERK1/2 or phospho-AKT analysis did not reveal any significant differences between the groups at this timepoint (Figure S14A and S14B). These results suggest that the concentration and distribution of the ligands rather than their differential signaling determine the biological effects of the VEGF-B isoform–specific vectors.

### Both VEGF-B Isoforms Expand VEGFB-iEC Population Indirectly via VEGFR-2 Activation

Analysis of VEGFB-iEC markers by RT-qPCR in heart lysates showed that the AAV9-VEGF-B vectors increase VEGFB-iECs transcript levels minimally at the 1-week timepoint (Figure S15A) and more robustly at 2 and 4 weeks (Figure S15A). The results also implied that AAV-VEGF-B_186_ is a stronger inducer of VEGFB-iECs than VEGF-B_167_ when both were delivered at the same dose. *Flt-1* gene deletion has been shown to increase VEGFR-2 signaling activity by depleting VEGFR-1 that acts as a decoy receptor for VEGF-A,^11,45^ thus promoting increased binding of endogenous VEGF-A to VEGFR-2.^11^ To further check whether the expansion of the VEGFB-iECs in adult mice is mediated by displacement of the endogenous VEGF-A from VEGFR-1 to VEGFR-2, we deleted VEGFR-1 in Cdh5 (cadherin 5)-CreER^T2^; VEGFR-1^fl/fl^ mice^46^ for 2 weeks, followed by analysis of the VEGFB-iEC markers. In addition, we included to the analysis mice with constitutive deletion of VEGFR-1 tyrosine kinase domain (VEGFR-1TK^−/−47^). The results showed that with the exception of *Cd24a* and *Col13a1*, all transcript markers were significantly increased in mice deleted of endothelial VEGFR-1 for 2 weeks but not in the mice lacking only the VEGFR-1 tyrosine kinase domain (Figure S15B and S15C). However, there were only a few Plvap-positive VEGFB-iECs in the immediate subendocardium in adult WT mice treated with the AAV9-VEGF-B_186_ vector versus scrambled control (Figure S16A).

To further confirm that the increase of VEGFB-iECs is mediated via VEGF-A displacement from VEGFR-1 to VEGFR-2, we deleted VEGFR-2 using Cdh5-CreER^T2,11,48^ followed by administration of AAV9-VEGF-B_186_ for 2 weeks (Figure S16B). The results confirmed that deletion of VEGFR-2 from ECs inhibits both the VEGF-B_186_-induced cardiac hypertrophy^11^ and the increase in VEGFB-iEC markers (Figure S16C and S16D). Accordingly, we conclude that amplification of VEGFB-iECs requires VEGFR-2 activity. We think that increased VEGF-B signaling in the adult heart targets the VEGFR-2-positive endocardial and vascular ECs and leads to preferential expansion of VEGFB-iECs as they have more abundant VEGFR-2 expression than other vascular ECs. The possibility remains that on ischemia/cardiac damage, some of the already established VEGFB-iECs display a strong contribution to VEGF-A-induced angiogenesis as suggested by Figure 6 in the study by Räsänen et al.^13^

## DISCUSSION

We demonstrate, using 2 transgenic models and viral vector-mediated gene transduction, that both paracrine and autocrine signaling by VEGF-B in the heart induce the expansion of a unique endocardium–derived cardiac EC population during the early postnatal period. Although the VEGFB-iECs are similarly induced in both VEGF-B transgenic models, the gradient induced by paracrine VEGF-B secretion in αMHC-VEGF-B mice leads to a well-organized VEGFB-iEC vessel gradient descending from the subendocardial vessels toward the subepicardium in the heart. In contrast, autocrine production of VEGF-B in cardiac ECs in aP2-VEGF-B mice disrupts this pattern and results in failure of VEGFB-iEC migration accompanied by a distinct phenotype marked by septal defects in newborn pups, massive cardiac hypertrophy in the surviving mice, and decreased cardiac function. Comparative analysis of mice from different experiments in which we deleted endocardial VEGFR-2, or endothelial VEGFR-1, as well as from mice lacking only VEGFR-1 tyrosine kinase domain, or mice expressing AAV9-VEGF-B_186_ but no VEGFR-2, indicates that the expansion of the VEGFB-iECs requires VEGFR-2 activity. In addition, we demonstrate that AAV-VEGF-B-mediated gene delivery to the adult heart causes a significant increase in VEGFB-iEC transcripts but only minimal expansion of VEGFB-iECs in the immediate subendocardial region. Furthermore, our comparison of the paracrine and autocrine models revealed significant cell-specific differences in the relative expression levels and decay rates of the VEGF-B_167_ and VEGF-B_186_ isoforms. These results should be relevant for the development of VEGF-B gene therapy for cardiovascular diseases.

One of the characteristic markers that define the VEGFB-iECs is *Plvap*,^49^ which is constitutively expressed only in the endocardium and few venous cells in the normal heart. *Plvap* is upregulated by VEGF-A signal transduction,^33,50,51^ in agreement with our finding that both transgenic and viral vector-mediated expressions of VEGF-B result in increased signaling by endogenous VEGF-A via VEGFR-2.^11^ Plvap forms multimers that form the spokes of the wheel-patterned fenestrations that characterize endothelia with high permeability in, for example, kidney glomerulus, choroid plexus, adrenal gland, and in several pathological conditions.^51^ A previous study by Li et al reported increased Plvap expression in the ischemic mouse and human heart and described it as a novel endothelial-specific marker of cardiac neovasculogenesis.^42^

The second most common marker of VEGFB-iECs is *Cd24a*, a glycosyl-phosphatidylinositol–linked surface glycoprotein, which acts as a ligand for P-selectin, a leukocyte adhesion receptor on activated ECs.^52^ *Cd24* silencing has been reported to decrease human umbilical vein EC migration and downregulated the expression of VEGF-A via inhibiting the phosphorylation and nuclear translocation of STAT3.^53^

*Esm1* expression marks VEGFB-iECs and the tip cells of angiogenic vessel sprouts, which have been shown to play a role in angiogenesis, inflammation, and vascular permeability. *Esm1* is required for optimal response to VEGF-A stimuli and VEGF-A-induced vascular permeability.^54^ Lineage tracing experiments have shown that the tip cells in angiogenic sprouts are derived from venous ECs, from which Esm1-positive ECs migrate into nearby vessels where they join with ECs of arterial identity.^54–56^ In our model, the expanded VEGFB-iECs showed only capillary markers, thus resembling angiogenic capillary EC populations.^57^

The *Foxf1* transcription factor, another VEGFB-iEC marker, has also a critical role in embryonic vasculature development and in vascular sprouting.^58,59^ EC deletion of *Foxf1* reduced EC proliferation, increased apoptosis, inhibited VEGF-A signaling, and decreased expression of EC genes that are critical for vascular development, such as *Vegfr-1*, *Vegfr-2*, and *Pecam1*.^58^ The *Exoc3l2* marker gene is involved in the targeting of exocytic vesicles to the cell surface.^60^ Interestingly, upregulation of Exoc3l2 was reported in response to VEGF-A stimulation of primary cultures of human ECs, and *Exoc3l2* silencing showed inhibition of VEGFR-2 phosphorylation and VEGF-A-directed migration of cultured ECs.^60^

*Col13*, a transmembrane collagen,^61,62^ has been concluded to enhance angiogenesis through a mechanism involving β1-integrins and the JNK pathway.^63^ Here, we confirmed the selective expression of Col13a1 only in the VEGFB-iECs by immunofluorescent staining, whereas the steady-state endothelium in the WT heart had little or no expression of Col13a1. It, thus, seems that several of the VEGFB-iEC markers represent transcripts that are regulated by VEGF-A during angiogenic processes, yet their assessment in the context of VEGF-A stimulation of cardiac neovascularization is made difficult by the toxicity of angiogenesis-inducing levels of VEGF-A.

The VEGFB-iECs were not affected during pregnancy, and they were only increased by 4.3% in WT mice subjected to LAD ligation. Interestingly, recent analyses of transcriptomic changes in ischemic human hearts and in mice after MI^64–67^ do not indicate significant upregulation of VEGFB-iEC marker transcripts. Thus, the unique ability of VEGF-B to expand the VEGFB-iEC population likely underlies the protective effects of VEGF-B in the ischemic heart.

Coronary vessel ECs arise from 2 distinct sources: the sinus venosus and the endocardium.^68^ The onset of endocardial contribution to coronary vessel formation has been debated recently. Some studies reported that a second wave of coronary ECs arises from the endocardium postnatally,^69^ while other studies challenged the idea of postnatal contribution and proposed prenatal contribution instead.^37,70^ Concerning this, the results of Tang et al^38^ are in agreement with a study showing that chemokine signaling mediated by the Cxcl12-Cxcr4 ligand-receptor pair is responsible for endocardium-derived EC migration and their contribution to coronary arteries.^37^ Our study shows that VEGF-B expression in cardiomyocytes or cardiac ECs boosts the endocardium-derived supply of VEGFB-iECs during the early postnatal period. We conclude that VEGFB-iECs are capillary-like ECs that integrate into coronary vessels and, thus, increase perfusion in the subendocardial myocardium.

In contrast to previous reports,^39^ we detected VEGFR-2 protein in segments of the adult endocardium. We have previously shown that VEGFR-2 protein is barely detectable in arteries^14^ that have high laminar blood flow–induced shear stress, which is known to upregulate *Klf-2* that, in turn, inhibits *Kdr* (VEGFR-2) transcription.^71^ Thus, our present study suggests that VEGFR-2 expression is dynamically regulated by the shear stress in endocardial segments between the cardiac trabeculae. The VEGFB-iECs in adult mice showed increased expression of *Kdr*, which should render them more susceptible to the angiogenic effect caused by VEGF-B-mediated displacement of VEGF-A from VEGFR-1 to VEGFR-2. Accordingly, these VEGFR-2-expressing endocardial cells and the few established VEGFB-iECs in WT mice could preferentially respond to VEGF-B signaling, leading to an increase of VEGFB-iECs in the adult subendocardium.

We also found that the mechanism of VEGF-B isoform regulation differs between cardiomyocytes and ECs. In particular, the VEGF-B_167_ protein was below our detection threshold in aP2-VEGF-B transgenic hearts, despite comparable levels of transcripts encoding the 2 isoforms in the same samples. This indicates that autocrine VEGF-B secretion by ECs results in lower levels of VEGF-B_167_ than its paracrine secretion from cardiomyocytes. Our data shows that similar levels of the isoform-specific RNAs encoded by the AAV vectors also result in more abundant expression of VEGF-B_186_ than VEGF-B_167_ protein in the heart, yet both proteins increased proliferation of cardiac ECs with similar time-dependent kinetics. Thus, VEGF-B_186_ induced more proliferation of ECs than VEGF-B_167_, presumably because VEGF-B_186_ has a higher concentration. However, a comparison of VEGF-B isoforms through probing of VEGFR-2 downstream signaling did not reveal any significant differences between the groups at 2 weeks after AAV delivery. This indicates cell type-specific posttranscriptional regulation of VEGF-B isoform expression in the heart. VEGF-B_167_ contains a heparan sulfate-binding domain sequestering it largely to the cell surface,^17^ which increases its cell surface concentration, resulting in a more rapid but apparently shorter-lived stimulation of ECs than with VEGF-B_186_. A likely reason for such difference is that unlike VEGF-B_167_, secreted VEGF-B_186_ escapes cell surface binding because it does not bind NRP-1 or heparan sulfate until it is cleaved by, as yet unknown protease(s), available in the EC microenvironment.

Cardiomyocytes produce VEGF-B, which binds VEGFR-1 and NRP-1 on the surface of ECs, inducing a shift of endogenous VEGF-A to VEGFR-2.^11,72–74^ VEGF-A signaling was shown to induce angiogenic sprouting in the early postnatal retina, where tip cell migration depends on a gradient of the heparan sulfate-binding VEGF-A_165_ isoform, whereas the VEGF-A_121_ isoform that does not bind to heparan sulfate and, therefore, does not form a concentration gradient promoted only EC proliferation in a concentration-dependent manner.^25^ Consistent with these phenotypes, the cardiac blood vessel density in the subendocardial area, where the vascular effects of paracrine VEGF-B signaling were most prominent, was lower in autocrine aP2-VEGF-B mice than in paracrine αMHC-VEGF-B mice. However, because of increased EC proliferation in the autocrine model, the vessels were larger in aP2-VEGF-B hearts than in the WT or αMHC-VEGF-B hearts. We hypothesize that despite retained cell proliferation in the VEGF-B-producing ECs, the cardiac septal defects in the autocrine aP2-VEGF-B mouse model arise from the lack of a VEGF-B gradient for angiogenic sprouting and EC migration. Because of the resulting angiocrine effect on other cardiac cells, the aP2-VEGF-B mice then develop a massive postnatal heart size with a scattered pattern of expanded VEGFB-iECs-positive vessels. We, thus, posit that the mismatch between cardiomyocyte growth and the vascular growth pattern leads to septal defects and pathological cardiac phenotype.

Interestingly, circulating VEGF-B levels have been found to be inversely correlated with ongoing left ventricular remodeling in patients with heart failure.^75,76^ Although numerous reports have indicated that increasing VEGF-B in the heart has translational potential for treatment of cardiac ischemia and heart failure, one should proceed to clinical studies with caution,^18–21,77^ as one study has reported ventricular arrhythmias in pigs subjected to VEGF-B_186_ adenoviral gene transfer before MI,^20^ while constitutive overexpression of human VEGF-B_167_ in cardiomyocytes caused mitochondrial lipotoxicity and subsequent increased death of adult transgenic mice.^24^ Despite these concerns, VEGF-B holds therapeutic potential due to its ability to redirect endogenous levels of VEGF-A to VEGFR-2 without inducing vascular leakage. By understanding the molecular and cellular mechanisms of VEGF-B action via increased endogenous VEGF-A-VEGFR-2 signaling, VEGF-B could eventually be safely tested as a time- and concentration-controlled therapeutic modality in cardiac ischemia. VEGF-B could be especially beneficial to combat age-related health issues as murine studies with minimal increase of VEGF-A signaling have suggested that counteracting age-related VEGF-A signaling insufficiency promotes healthy aging and extends life span.^78^

## ARTICLE INFORMATION

### Acknowledgments

The authors thank Mari Jokinen, Maria Arrano de Kivikko, Tapio Tainola, Päivi Leinikka, Tanja Laakkonen, and Jarmo Koponen for excellent technical assistance and Sinem Karaman, Eva Domenech Moreno, Pirjo Laakkonen, Emilia A. Korhonen, Chad S. Weldy, Olli Ritvos, Emma Viitala, and Risto Kerkelä for scientific discussions. They also thank the Biomedicum Imaging Unit, the FIMM Digital Microscopy and Molecular Pathology Unit, the Biomedicum Flow Cytometry Unit, and the Laboratory Animal Center of the University of Helsinki for professional services. The authors thank HelVi/AAV unit of the University of Helsinki for providing AAVs.

### Author Contributions

I. Sultan, M. Ramste, P. Peletier, R. Kivelä, and K. Alitalo designed the study. I. Sultan, M. Ramste, P. Peletier, K.A. Hemanthakumar, D. Ramanujam, A. Tirronen, Y. von Wright, and S. Antila performed experiments and acquired the data. I. Sultan analyzed the data and prepared the figures. I. Sultan and K. Alitalo interpreted the data. K. Alitalo supervised the study and provided funding. I. Sultan, R. Kivelä, and K. Alitalo wrote the article. I. Sultan and P. Peletier performed all experiments to address reviewers’ comments. I. Sultan, P. Peletier, and K. Alitalo wrote the reviewers’ response letter. L. Eklund contributed to the generation of autocrine VEGF-B (vascular endothelial growth factor B) expression in cardiac endothelium transgenic mouse lines and provided Col13a1 antibodies. E. Mervaala contributed to the left anterior descending coronary artery ligation experiments and to the transverse aortic constriction experiments in the revision phase. P. Saharinen contributed to vascular leakage experiments in the revision phase. Magnetic resonance imaging acquisition was performed in the laboratory of S. Ylä-Herttuala. Validation of VEGF-B expression in different cardiac cell types was contributed by D. Rananujam and S. Engelhardt. All authors have seen, commented, and accepted the article.

### Sources of Funding

This work was supported by the European Union’s Horizon 2020 Research and Innovation Programme under Grant Agreement 874708 (K. Alitalo, Theralymph), the Sigrid Jusélius Foundation (to K. Alitalo), the Novo Nordisk Foundation (to K. Alitalo, grant NNF16OC0023554), the Hospital District of Helsinki and Uusimaa Research Grant (to K. Alitalo, grant TYH2022202), the Jane and Aatos Erkko Foundation (to K. Alitalo), and the Research Council of Finland (to K. Alitalo, grant 314498). I. Sultan received support from the University of Helsinki Research Foundation, the Aarne Koskelo Foundation, the Finnish Foundation for Cardiovascular Research, the Paavo Nurmi Foundation, the Aarne and Aili Turunen Foundation, the Einar och Karin Stroems stiftelse (Finska Läkaresällskapet), the Paulo Foundation, the Orion Research Foundation sr, the Biomedicum Helsinki Foundation, the Ida Montin Foundation, and the Maud Kuistila Memorial Foundation. M. Ramste received support from the Paavo Nurmi Foundation, the Finnish Foundation for Cardiovascular Research, the Biomedicum Helsinki Foundation, and the Emil Aaltonen Foundation. S. Antila received support from the Paavo Nurmi Foundation and the Päivikki and Sakari Sohlberg Foundation. P. Saharinen received support from European Union's Horizon 2020 research and innovation programme under grant agreement number 773076, the Sigrid Jusélius Foundation, and the Academy of Finland center of Excellence Program (grant 346134). E. Mervaala received support from the Finnish Foundation for Cardiovascular Research. S. Engelhardt was supported by the Deutsche Forschungsgemeinschaft (DFG; grant TRR 267) and the Federal Ministry of Education and Research (BMBF) in the framework of the Cluster4futureprogram (CNATM-Cluster for Nucleic Acid Therapeutics Munich). R. Kivelä received support from the Sigrid Jusélius Foundation and the Finnish Foundation for Cardiovascular Research.

### Disclosures

None.

### Supplemental Material

Supplemental Detailed Methods

Uncropped Western Blots

ARRIVE Checklist

Sciscore Report

Figures S1–S16

Tables S1–S4

Major Resources Table

## Supplementary Material
