# Supplementary material for: Contribution of VEGF-B-Induced Endocardial Endothelial Cell Lineage in Physiological Versus Pathological Cardiac Hypertrophy
Source: Circ Res. 2024 Apr 24;134(11):1465–82. doi: 10.1161/CIRCRESAHA.123.324136 (PMC11542978; doi:10.1161/CIRCRESAHA.123.324136)

Uncropped Western Blot images

* Blots that are placed next to each other show detections of different protein targets from the same gel.

* The red rectangle indicates the part of the blot that is presented in the manuscript. The orange rectangle indicates the part that has been used only for quantifications.

Main Figures:

Full unedited gel for Fig.1 (D)

Left panel, **β-actin (45 kDa) (1:10000, #4967, Cell Signaling).** Right panel, **VEGF-B (21 and 32 kDa) (1:1000, #AF590, R&D Systems)**.

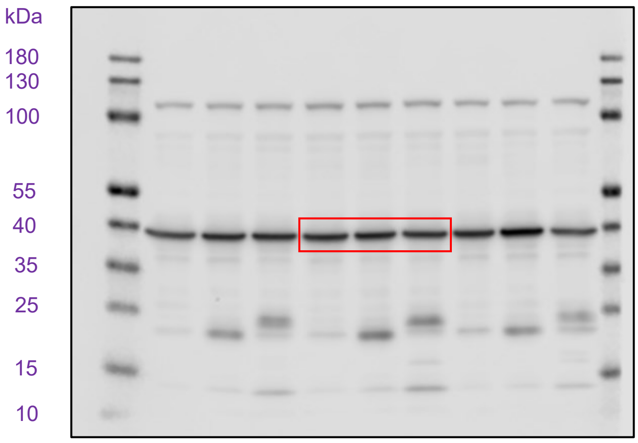

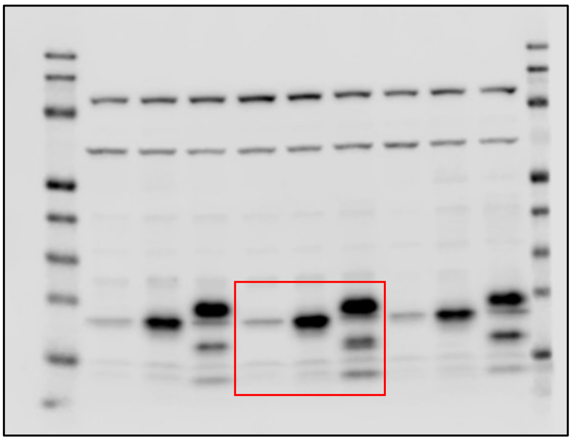

Full unedited gel for Fig.1 (D)

Left panel, **β-actin (45 kDa) (1:10000, #4967, Cell Signaling).** Right panel, **VEGF-B (21 and 32 kDa) (1:1000, #AF590, R&D Systems)**.

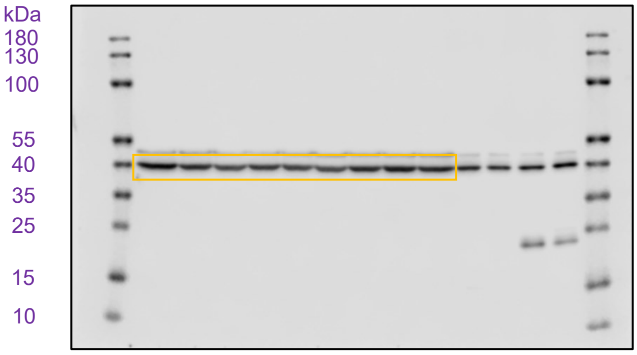

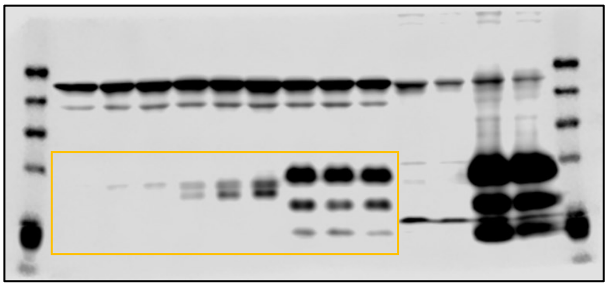

Full unedited gel for Fig.1 (D)

Left panel, **β-actin (45 kDa) (1:10000, #4967, Cell Signaling).** Right panel, **VEGF-B (32 kDa) (1:1000, #AF590 + #AF751, R&D Systems)**.

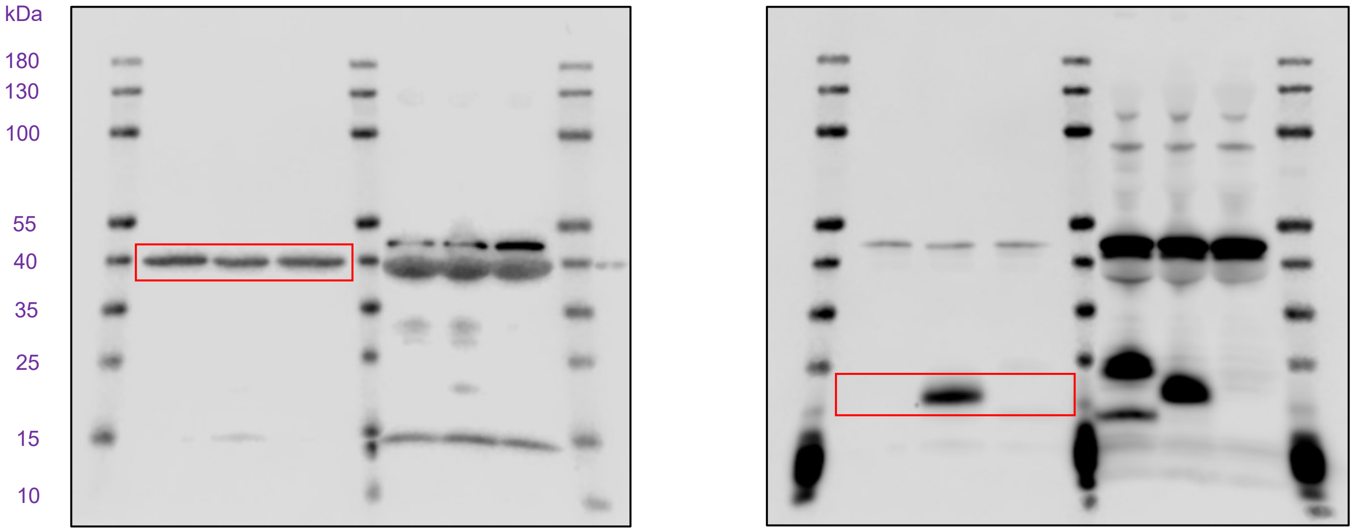

Full unedited gel for Fig.4 (E)

Left panel, **HSC70 (70 kDa) (1:10000, #SC-7298, Santa Cruz Biotechnology)** and **VEGF-B (21 and 32 kDa) (1:1000, #AF751, R&D Systems)**. Right panel, **VEGFR-1 (100 kDa) (1:1000, #AF471, R&D Systems).**

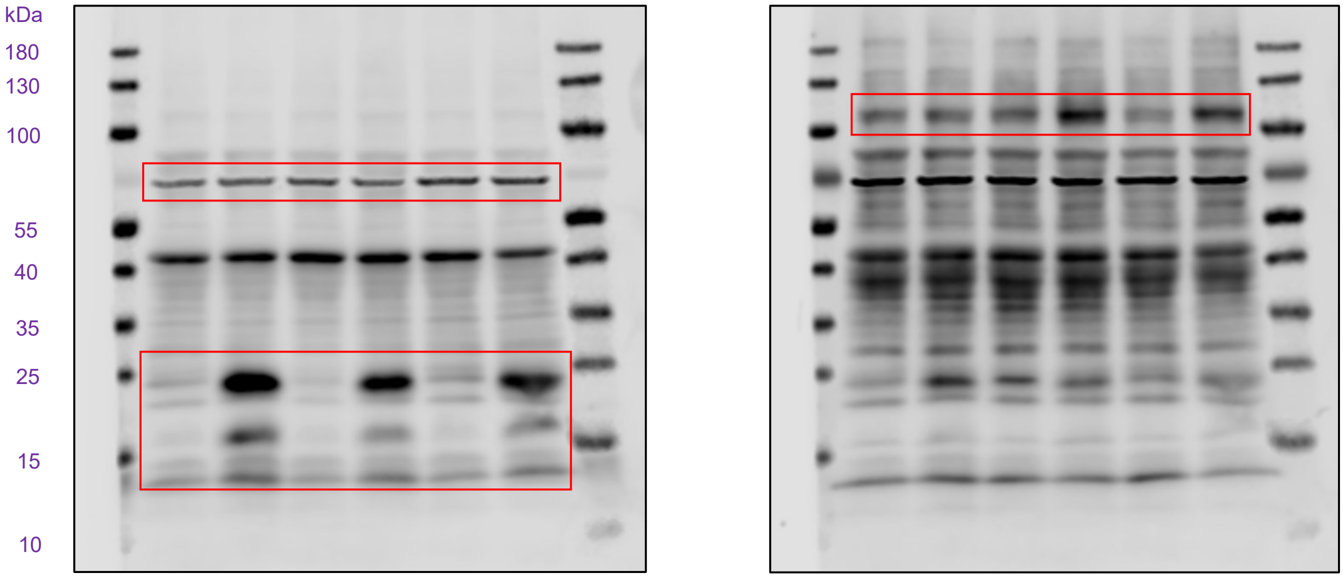

Full unedited gel for Fig.4 (E)

Left panel, **HSC70 (70 kDa) (1:10000, #SC-7298, Santa Cruz Biotechnology)**. Right panel, **VEGFR-2 (230 kDa) (1:1000, #AF644, R&D Systems).**

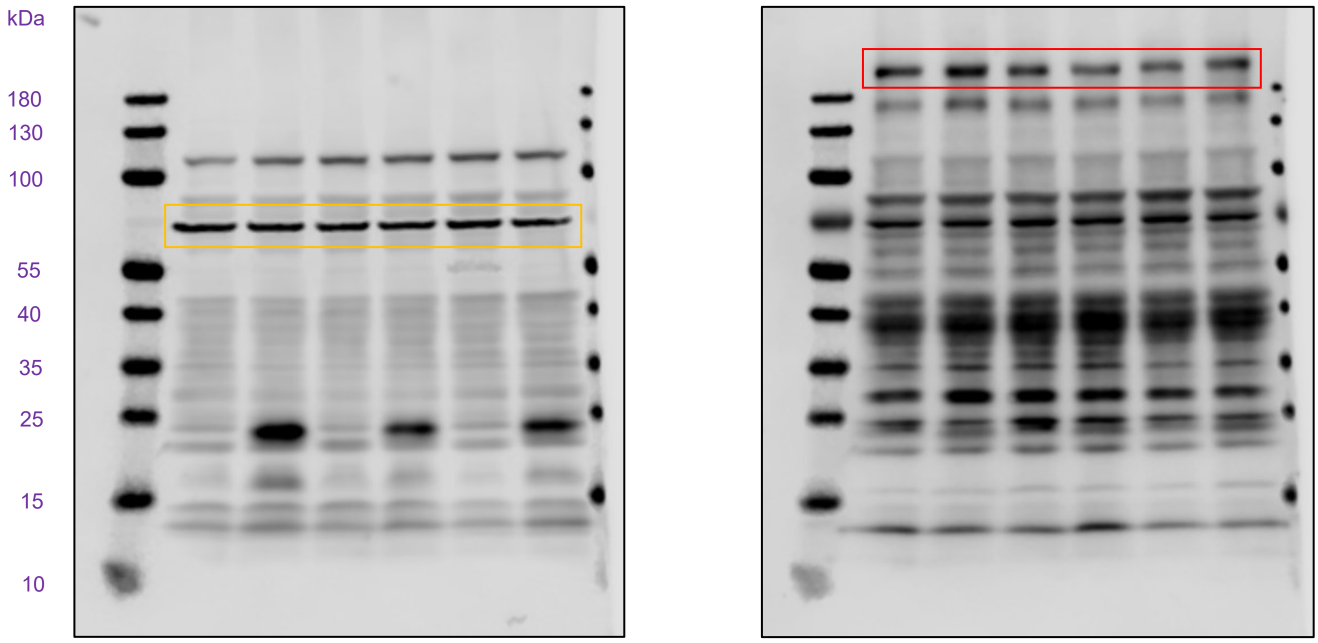

Supplementary results

Full unedited gel for Fig.12 (D)

VEGF-B (21 and 32 kDa) (1:1000, #AF590, R&D Systems).

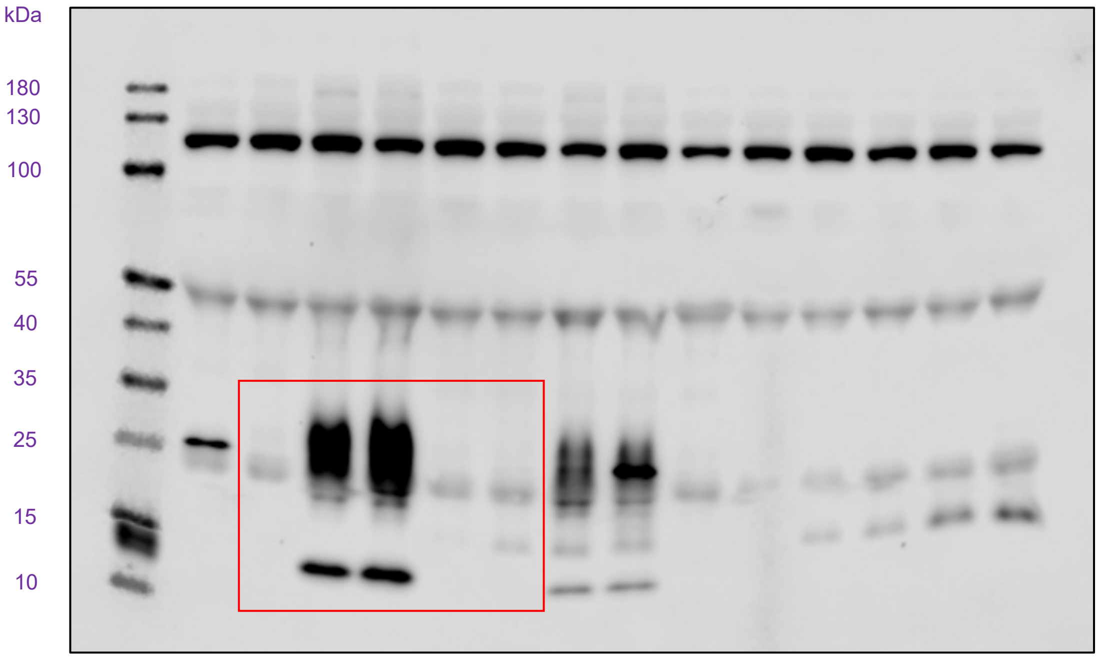

Full unedited gel for Fig.12 (E)

VEGF-B (21 and 32 kDa) (1:1000, #AF590, R&D Systems).

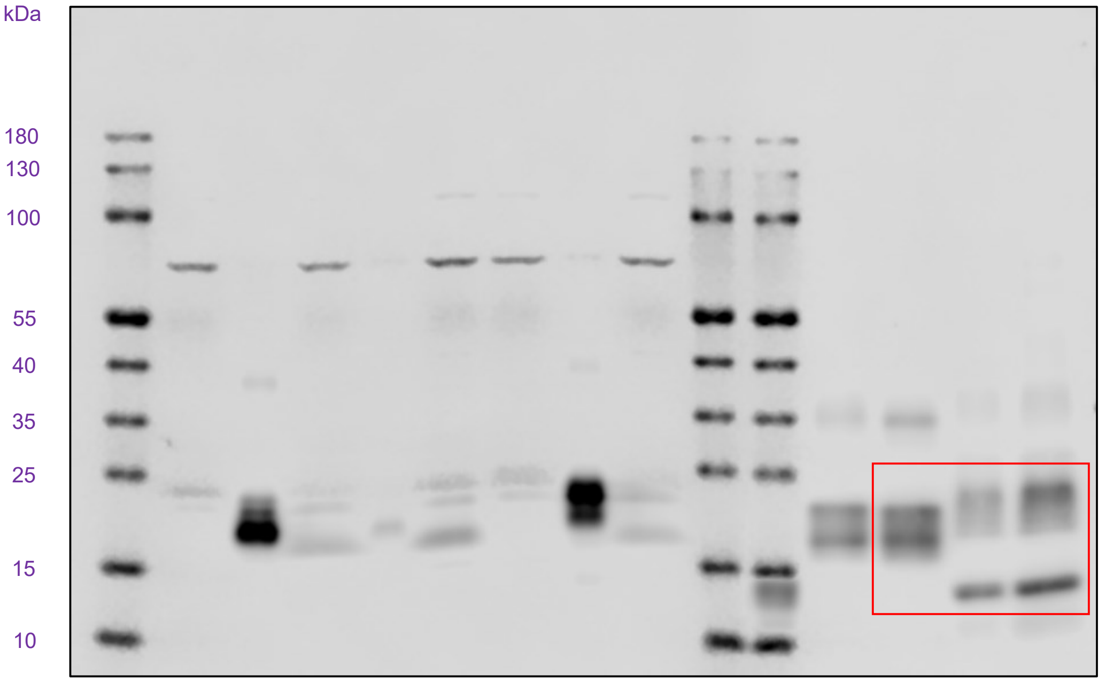

Full unedited gel for Fig.12 (H)

HSC70 (70 kDa) (1:10000, #SC-7298, Santa Cruz Biotechnology) and **VEGF-B (21 and 32 kDa) (1:1000, #AF590, R&D Systems).**

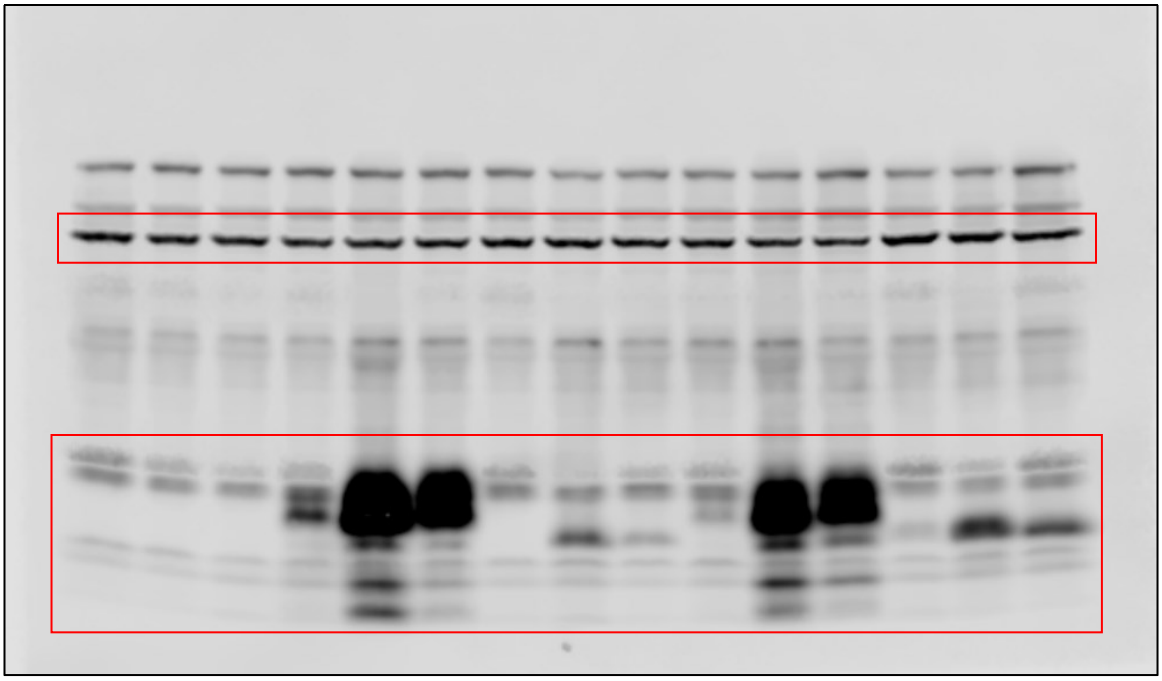

Full unedited gel for Fig.14 (A)

Left panel, **p-VEGFR-1 (Y1213) (130 kDa) (1:1000, #AF4170, R&D Systems).** Right panel, **VEGFR-1 (100 and 180 kDa) (1:1000, #AF471, R&D Systems).**

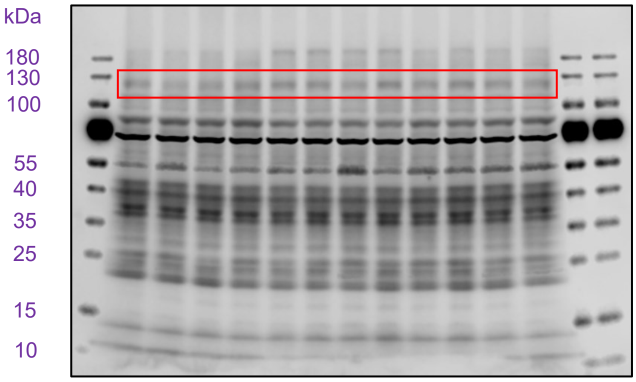

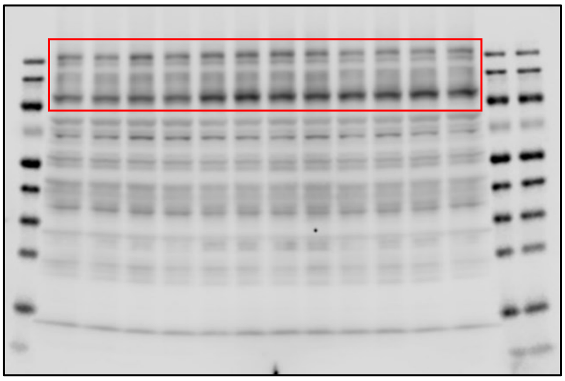

Full unedited gel for Fig.14 (A)

Left panel, **VEGFR-2 (150 and 230 kDa) (1:1000, #AF644, R&D Systems).** Right panel, **HSC70 (70 kDa) (1:10000, #SC-7298, Santa Cruz Biotechnology).**

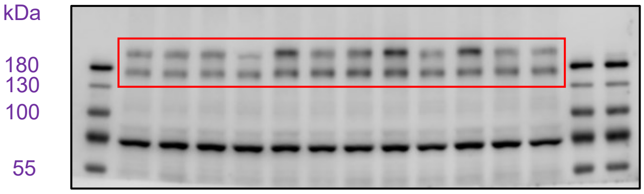

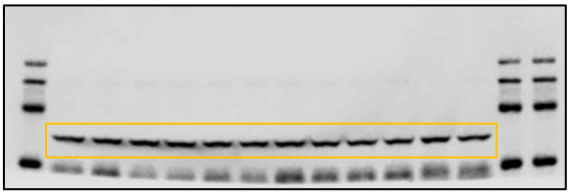

Full unedited gel for Fig.14 (A)

Left panel, **HSC70 (70 kDa) (1:10000, #SC-7298, Santa Cruz Biotechnology).** Right panel, **NRP-1 (120 kDa) (1:1000, #AF566, R&D Systems).**

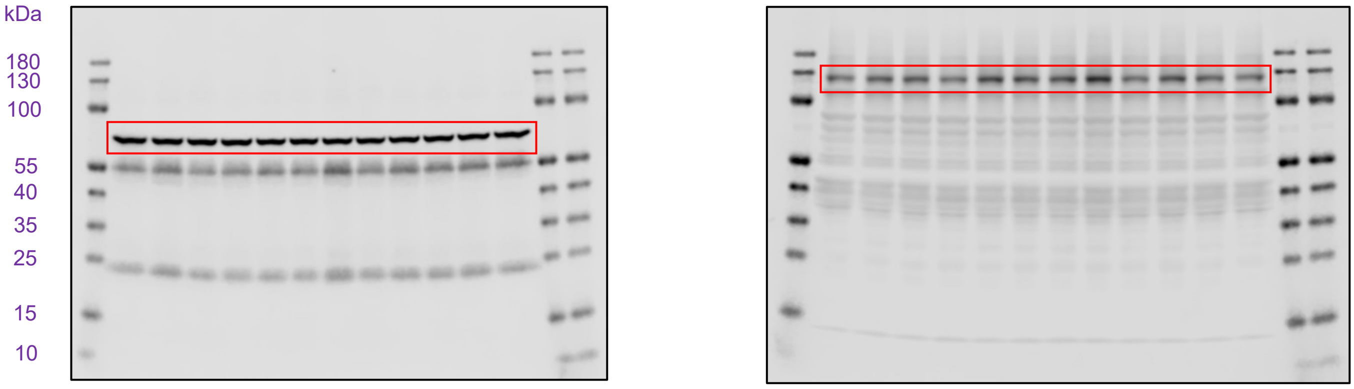

Full unedited gel for Fig.14 (A)

Left panel, **p-AKT (Ser473) (60 kDa) (1:1000, #9271, Cell Signaling).** Right panel, **AKT (60 kDa) (1:1000, #9272, Cell Signaling).**

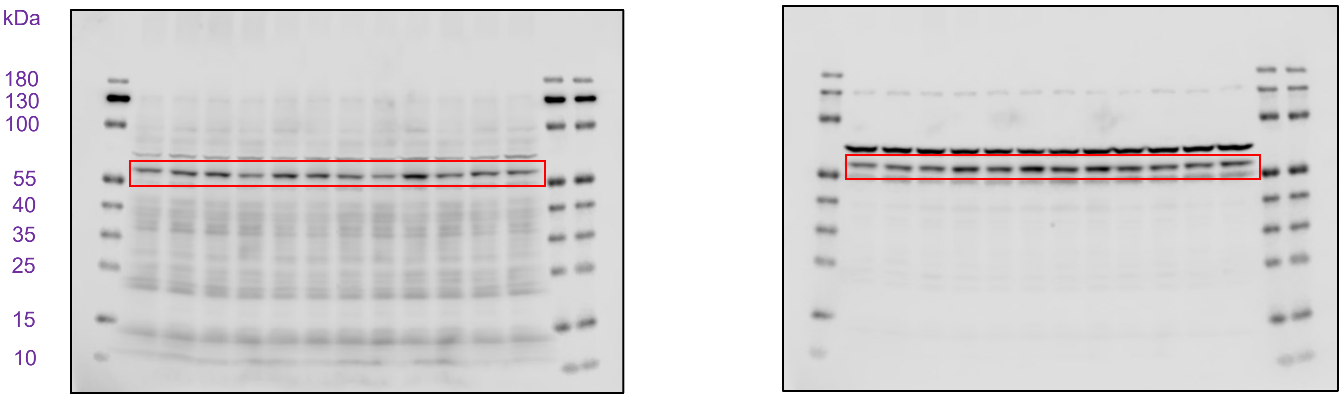

Full unedited gel for Fig.14 (A)

Left panel, **p-p44/42 MAPK (Erk1/2) (Thr202/Tyr204) (42 and 44 kDa) (1:1000, #9101, Cell Signaling).** Right panel, **p44/42 MAPK (Erk1/2) (42 and 44 kDa) (1:1000, #9102, Cell Signaling).**

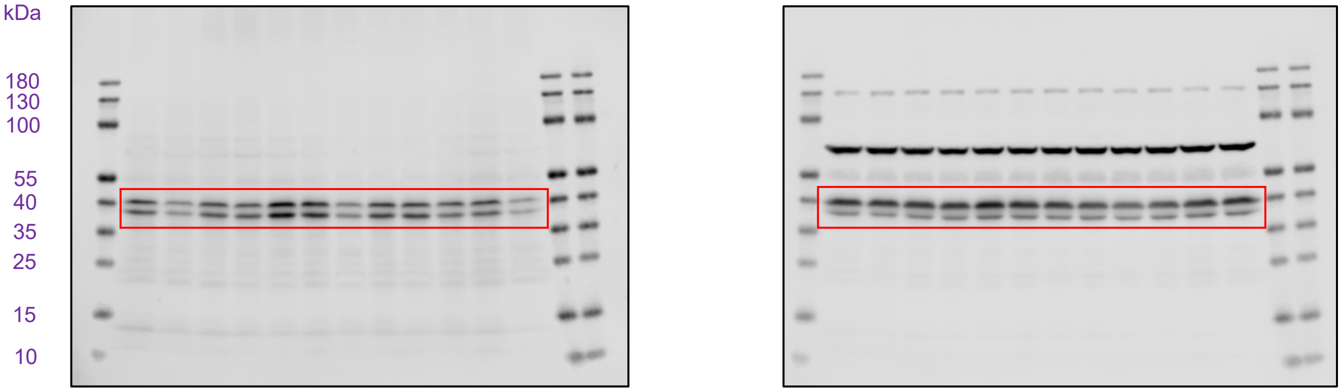

Reviewer questions

Full unedited gel for Fig.2 (A)

HSC70 (70 kDa) (1:10000, #SC-7298, Santa Cruz Biotechnology) and **VEGF-B (21 and 32 kDa) (1:1000, #AF751, R&D Systems)**.

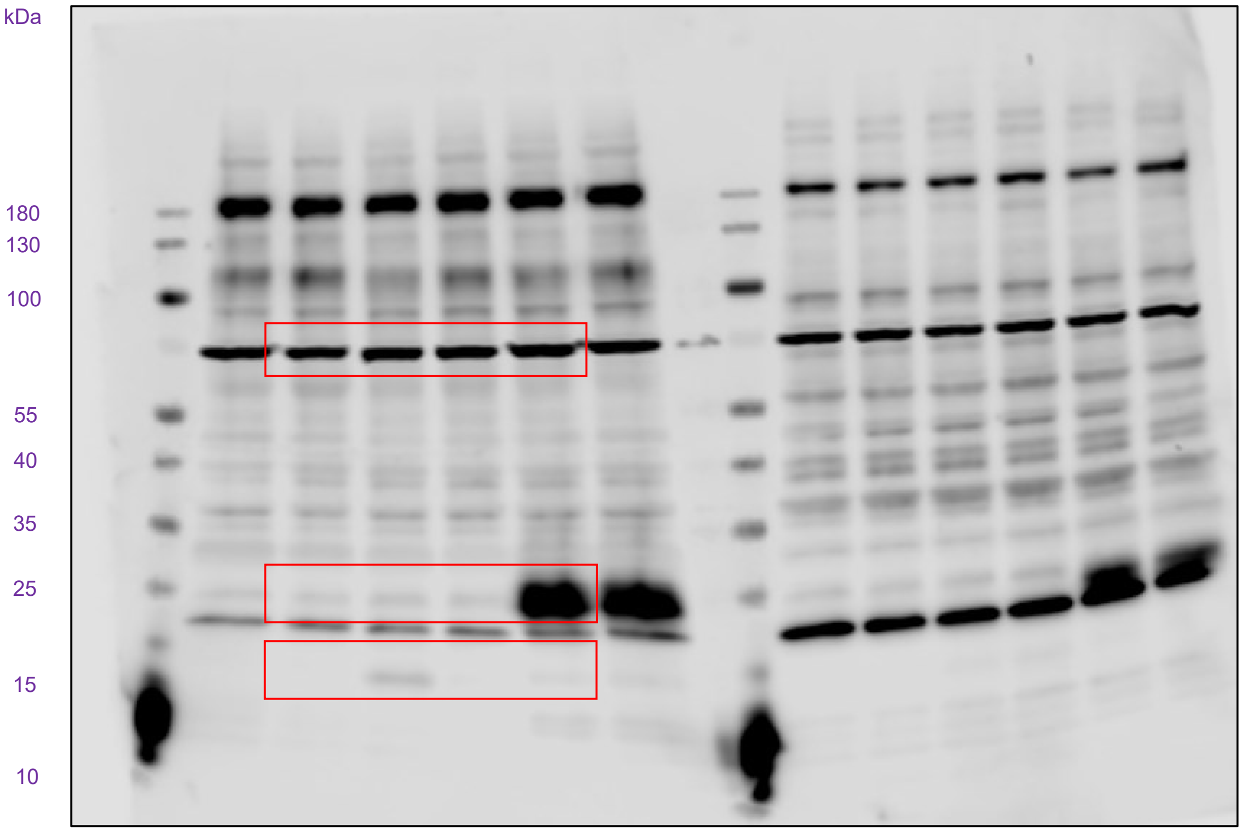

Full unedited gel for Fig.6 (B)

HSC70 (70 kDa) (1:10000, #SC-7298, Santa Cruz Biotechnology) and **VEGF-B (21 and 32 kDa) (1:1000, #AF590, R&D Systems)**.

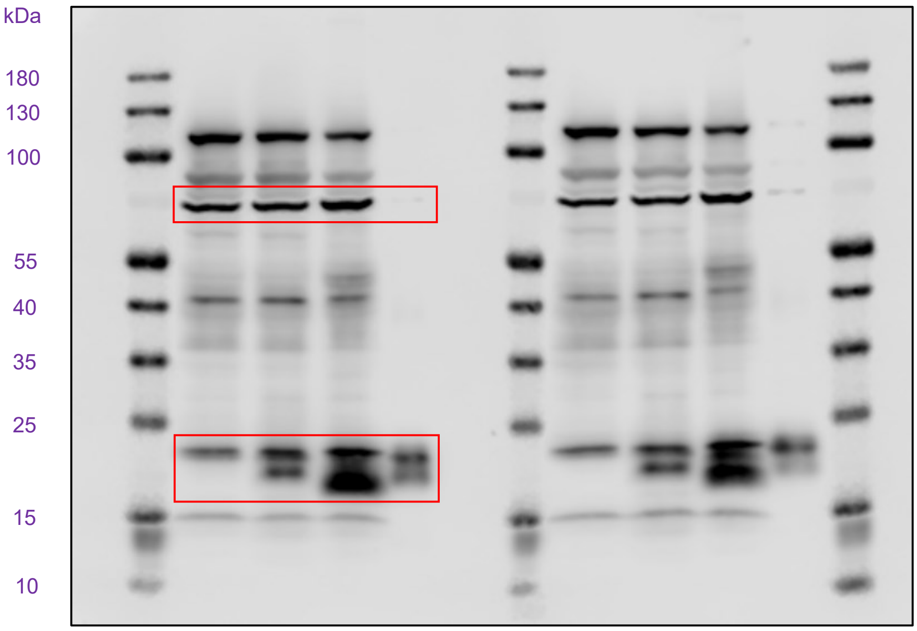

Full unedited gel for Fig.14

Left panel, **β-actin (45 kDa) (1:10000, #4967, Cell Signaling).** Right panel, **VEGF-B (21 and 32 kDa) (1:1000, #AF590 + #AF751, R&D Systems)**.

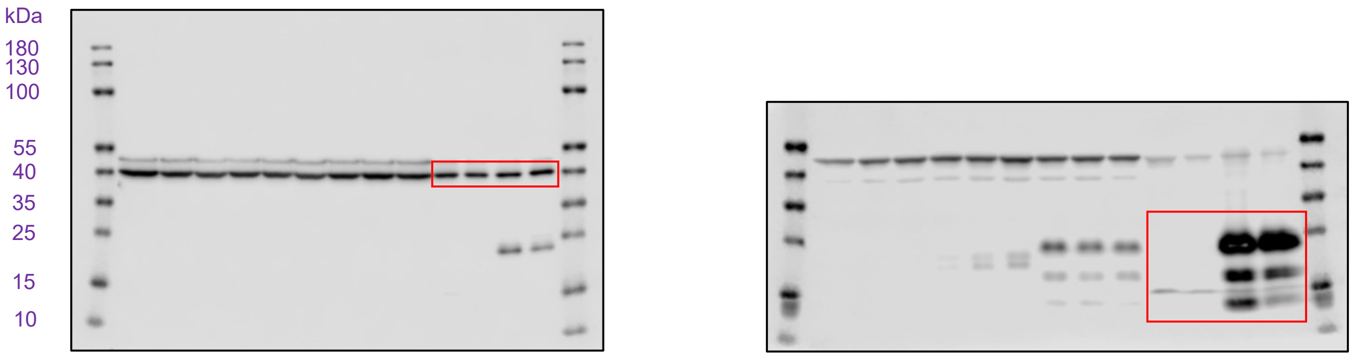

Full unedited gel for Fig.19 (A)

Left panel, **HSC70 (70 kDa) (1:10000, #SC-7298, Santa Cruz Biotechnology).** Right panel, **GFP (44, 48.9, 48.9, and 49.2 kDa) (1:1000, #ab5450, Abcam).**

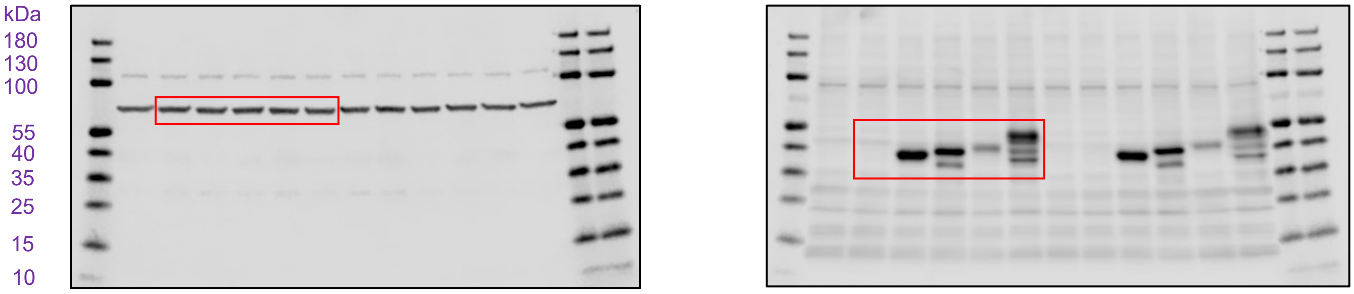

Full unedited gel for Fig.19 (B)

GFP (48.9, 48.9, 49.2, and 44 kDa) (1:1000, #ab5450, Abcam).

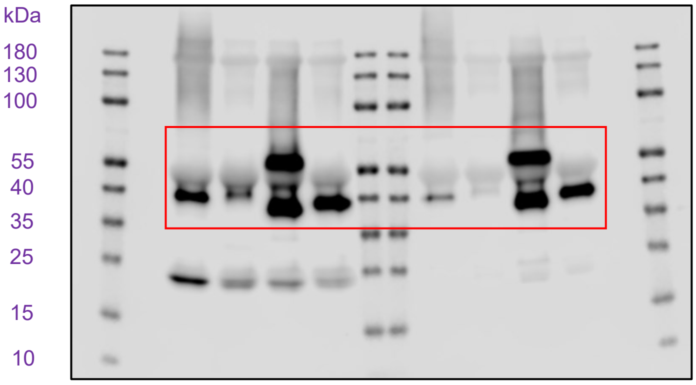

Full unedited gel for Fig.19 (C)

HSC70 (70 kDa) (1:10000, #SC-7298, Santa Cruz Biotechnology) and **GFP (48.9, 48.9, 49.2, and 44 kDa) (1:1000, #ab5450, Abcam).**

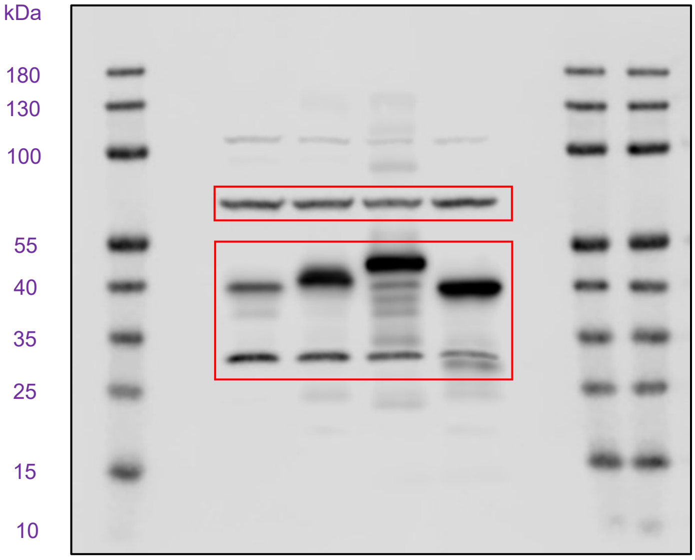

Full unedited gel for Fig.20 (A)

Left panel, **VEGF-B (21 kDa) (1:1000, #AF590, R&D Systems)**. Right panel, **HSC70 (70 kDa) (1:10000, #SC-7298, Santa Cruz Biotechnology)**.

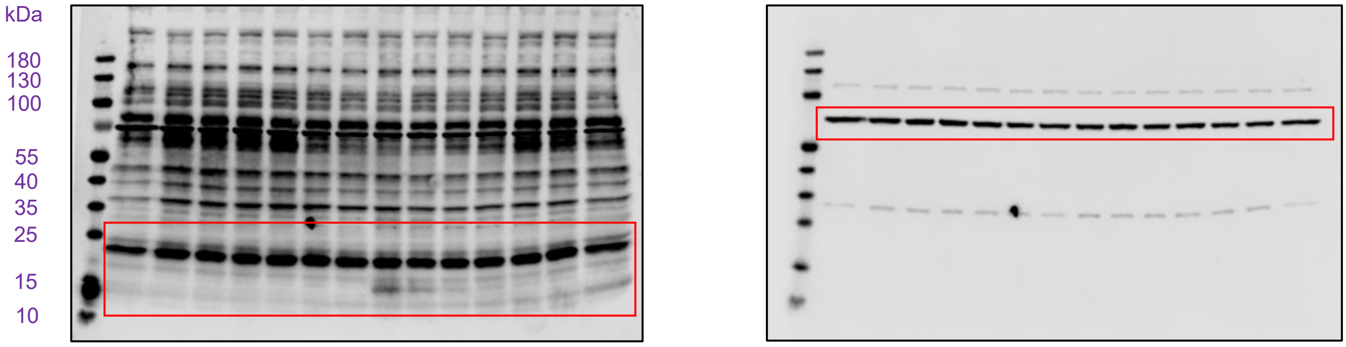

Full unedited gel for Fig.20 (A)

Left panel, **VEGF-B (32 kDa) (1:1000, #AF590, R&D Systems)**. Right panel, **HSC70 (70 kDa) (1:10000, #SC-7298, Santa Cruz Biotechnology)**.

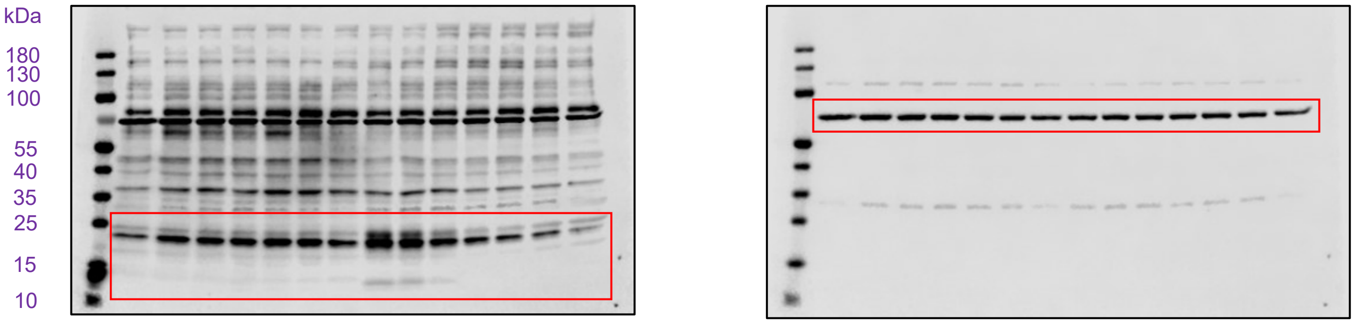

Full unedited gel for Fig.20 (B)

Left panel, **VEGF-B (32 kDa) (1:1000, #AF590, R&D Systems)**. Right panel, **HSC70 (70 kDa) (1:10000, #SC-7298, Santa Cruz Biotechnology)**.

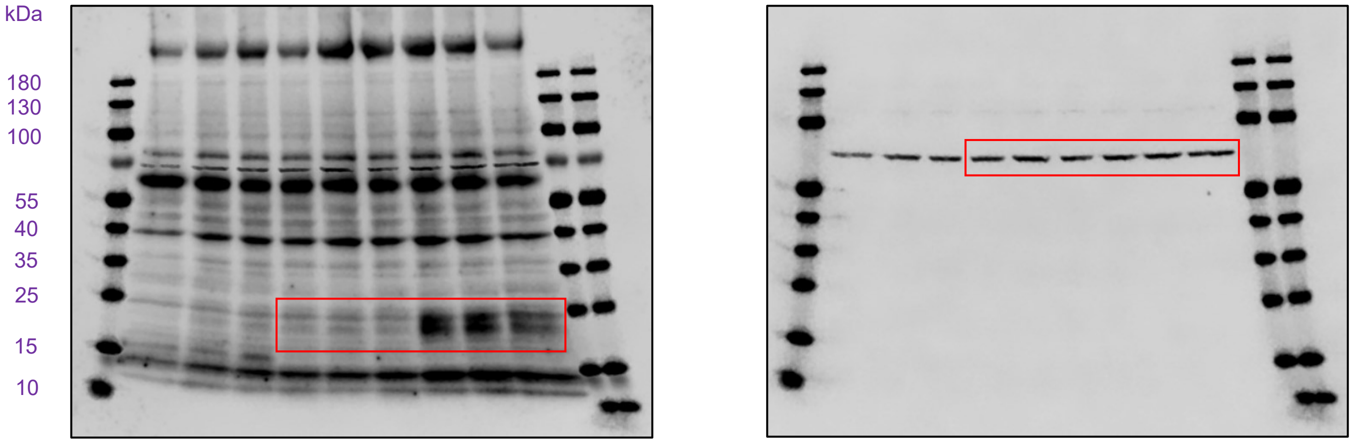

Full unedited gel for Fig.20 (B)

Left panel, **VEGF-B (21 kDa) (1:1000, #AF590, R&D Systems)**. Right panel, **HSC70 (70 kDa) (1:10000, #SC-7298, Santa Cruz Biotechnology)**.

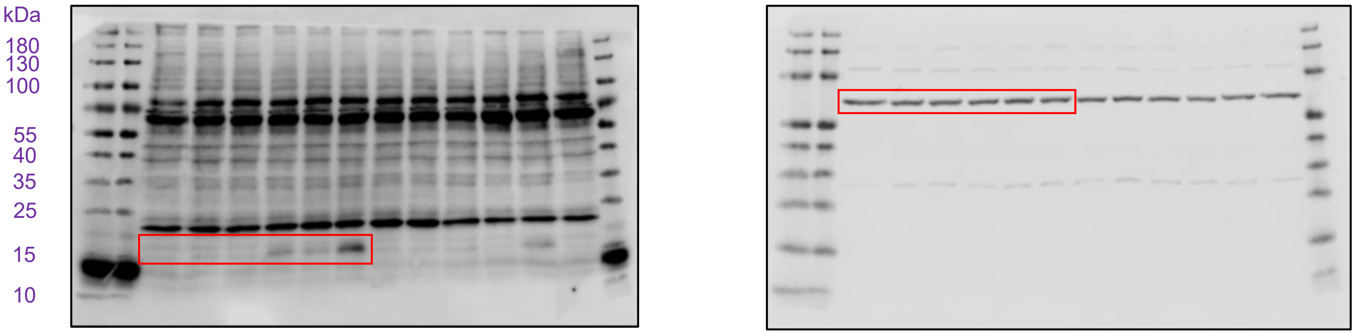

Full unedited gel for Fig.24 (B)

Left panel, **NRP-1 (120 kDa) (1:1000, #AF566, R&D Systems)**. Right panel, **β-actin (45 kDa) (1:10000, #4967, Cell Signaling)**.

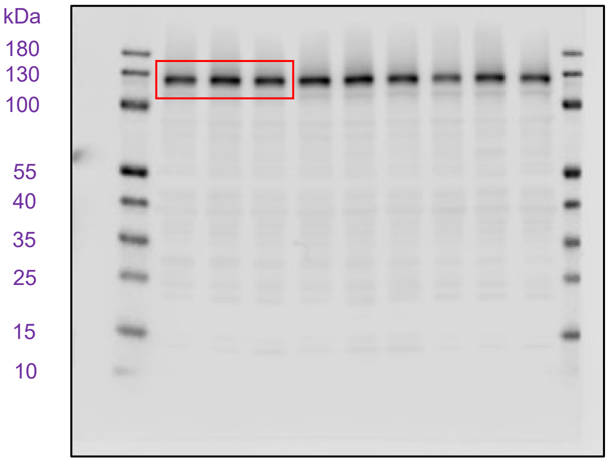

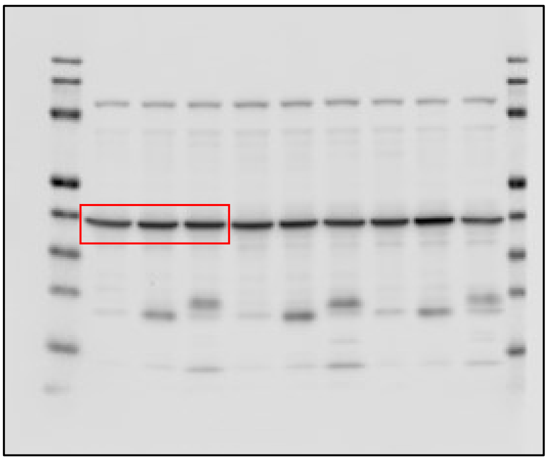

Supplement: Supplementary file 5 [file res-134-1465-s005.pdf]
