# Supplementary material for: Contribution of VEGF-B-Induced Endocardial Endothelial Cell Lineage in Physiological Versus Pathological Cardiac Hypertrophy
Source: Circ Res. 2024 Apr 24;134(11):1465–82. doi: 10.1161/CIRCRESAHA.123.324136 (PMC11542978; doi:10.1161/CIRCRESAHA.123.324136)
Supplement: Supplementary file 6 [file res-134-1465-s006.pdf]

Major Resources Table

Antibodies

Target antigen	Source	Catalog #	Working concentration / dilution	Persistent ID / URL
FABP4 (rabbit anti-human)	Abcam	#ab13979	1/200	RRID:AB_1951817 / https://www.abcam.com/en-fi/products/primary-antibodies/fabp4-antibody-ab13979
Plvap (rat anti-mouse)	BD Biosciences	#553849	1/150	RRID:AB_395086 / https://www.bdbiosciences.com/en-eu/products/reagents/western-blotting-and-molecular-reagents/western-blot-reagents/purified-rat-anti-mouse-panendothelial-cell-antigen.553849
Plvap (rabbit anti-mouse)	Cell Signaling	#82489	1/100	N/A / https://www.cellsignal.com/products/primary-antibodies/plvap-antibody/82489
Podocalyxin (goat anti-mouse)	R&D Systems	#AF1556	1/200	RRID:AB_354858 / https://www.rndsystems.com/products/mouse-podocalyxin-antibody_af1556
Dystrophin (mouse anti-mouse)	Leica Biosystems	#NCL-DYS2	1/200	RRID:AB_442081 / https://shop.leicabiosystems.com/ihc-ish/ihc-primary-antibodies/pid-dystrophin-antibodies
Cd31 (rat anti-mouse)	BD Biosciences	#553370	1/100	RRID:AB_394816 / https://www.bdbiosciences.com/en-us/products/reagents/flow-cytometry-reagents/research-reagents/single-color-antibodies-ruo/purified-rat-anti-mouse-cd31.553370
Col13a1 (rabbit anti-human)	Atlas Antibodies	#HPA050392	1/75	RRID:AB_2681110 / https://www.atlasantibodies.com/products/primary-antibodies/triple-a-polyclonals/anti-col13a1-antibody-hpa050392/
Actin α-Smooth Muscle-Cy3 conjugated (mouse anti-mouse)	Sigma-Aldrich	#C6198	1/1000	RRID:AB_476856 / https://www.sigmaaldrich.com/FI/en/product/sigma/c6198
Nr2f2 (mouse anti-human)	R&D Systems	#PP-H7147-00	1/150	RRID:AB_2155627 / https://www.rndsystems.com/products/human-coup-tf-ii-nr2f2-antibody-h7147_pp-h7147-00
Dll4 (goat anti-mouse)	R&D Systems	#AF1389	1/100	RRID:AB_354770 / https://www.rndsystems.com/products/mouse-dll4-antibody_af1389
CD45 (rabbit anti-mouse)	Abcam	#ab10558	1/250	RRID:AB_442810 / https://www.abcam.com/en-fi/products/primary-antibodies/cd45-antibody-ab10558
CD206 (goat anti-mouse)	R&D Systems	#AF2535	1/1000	RRID:AB_2063012 / https://www.rndsystems.com/products/mouse-mmr-cd206-antibody_af2535
CD24 (rat anti-mouse)	Invitrogen	#14-0242-82	1/500	RRID:AB_467170 / https://www.thermofisher.com/antibody/product/CD24-Antibody-clone-M1-69-Monoclonal/14-0242-82
RFP (goat)	Rockland	#200-101-379	1/250	RRID:AB_2744552 / https://www.rockland.com/categories/primary-antibodies/rfp-antibody-200-101-379/
VEGF-B (goat anti-mouse)	R&D Systems	#AF590	1/250 (IHC) / 1/1000 (WB)	RRID:AB_355463 / https://www.rndsystems.com/products/mouse-vegf-b-167-186-antibody_af590
VEGF-B (goat anti-human)	R&D Systems	#AF751	1/1000	RRID:AB_355571 / https://www.rndsystems.com/products/human-vegf-b-167-186-antibody_af751
VEGFR-1 (goat anti-mouse)	R&D Systems	#AF471	1/1000	RRID:AB_355379 / https://www.rndsystems.com/products/mouse-vegfr1-flt-1-antibody_af471

VEGFR-2<br>(goat anti-mouse)	R&D Systems	#AF644	1/250 (IHC) / 1/1000 (WB)	RRID:AB_355500 / https://www.rndsystems.com/products/mouse-vegfr2-kdr-flk-1-antibody_af644
phospho-VEGFR-1 (Y1213)<br>(rabbit anti-human)	R&D Systems	#AF4170	1/1000	RRID:AB_884545 / https://rndsystems.com/products/human-phospho-vegfr1-flt-1-y1213-antibody_af4170
NRP-1<br>(goat anti-mouse/rat)	R&D Systems	#AF566	1/1000	RRID:AB_355445 / https://www.rndsystems.com/products/mouse-rat-neuropilin-1-antibody_af566
phospho-AKT (Ser473)<br>(rabbit anti-mouse)	Cell Signaling	#9271	1/1000	RRID:AB_329825 / https://www.cellsignal.com/products/primary-antibodies/phospho-akt-ser473-antibody/9271
AKT<br>(rabbit anti-mouse)	Cell Signaling	#9272	1/1000	RRID:AB_329827 / https://www.cellsignal.com/products/primary-antibodies/akt-antibody/9272
phospho-p44/42 MAPK (Erk1/2) (Thr202/Tyr204)<br>(rabbit anti-mouse)	Cell Signaling	#9101	1/1000	RRID:AB_331646 / https://www.cellsignal.com/products/primary-antibodies/phospho-p44-42-mapk-erk1-2-thr202-tyr204-antibody/9101
p44/42 MAPK (Erk1/2)<br>(rabbit anti-mouse)	Cell Signaling	#9102	1/1000	RRID:AB_330744 / https://www.cellsignal.com/products/primary-antibodies/p44-42-mapk-erk1-2-antibody/9102
β-actin<br>(rabbit anti-mouse)	Cell Signaling	#4967	1/10000	RRID:AB_330288 / https://www.cellsignal.com/products/primary-antibodies/b-actin-antibody/4967
HSC70<br>(mouse anti-mouse)	Santa Cruz Biotechnology	#SC-7298	1/10000	RRID:AB_627761 / https://www.scbt.com/p/hsc-70-antibody-b-6
VEGF-B_{186} biotinylated<br>(goat anti-mouse)	R&D Systems	#BAF767	1/500	RRID:AB_2213298 / https://rndsystems.com/products/mouse-vegf-b-186-biotinylated-antibody_baf767
CD16/CD32, clone 2.4G2<br>(rat anti-mouse)	BD Biosciences	#BDB553142	1/100	RRID:AB_394656 / https://www.bdbiosciences.com/en-fi/products/reagents/flow-cytometry-reagents/research-reagents/single-color-antibodies-ruo/purified-rat-anti-mouse-cd16-cd32-mouse-bd-fc-block.553142
CD45-FITC, clone 30-F11<br>(rat anti-mouse)	STEMCELL Technologies	#60030FI.1	1/100	RRID:AB_312972 / https://www.stemcell.com/products/anti-mouse-cd45-antibody-clone-30-f11.html
PDGFR-PECy7, clone APA5<br>(rat anti-mouse)	Invitrogen	#25-1401-82	1/100	RRID:AB_2573400 / https://www.thermofisher.com/antibody/product/CD140a-PDGFRA-Antibody-clone-APA5-Monoclonal/25-1401-82
Cd105-PE, clone MJ7/18<br>(rat anti-mouse)	Invitrogen	#12-1051-82	1/100	RRID:AB_657524 / https://www.thermofisher.com/antibody/product/CD105-Endoglin-Antibody-clone-MJ7-18-Monoclonal/12-1051-82
Alexa Fluor 488<br>(donkey anti-goat)	Invitrogen	#A-11055	1/500	RRID:AB_2534102 / https://www.thermofisher.com/antibody/product/Donkey-anti-Goat-IgG-H-L-Cross-Adsorbed-Secondary-Antibody-Polyclonal/A-11055
Alexa Fluor 488<br>(donkey anti-rabbit)	Invitrogen	#A-21206	1/500	RRID:AB_2535792 / https://www.thermofisher.com/antibody/product/Donkey-anti-Rabbit-IgG-H-L-Highly-Cross-Adsorbed-Secondary-Antibody-Polyclonal/A-21206
Alexa Fluor 488<br>(donkey anti-mouse)	Invitrogen	#A-21202	1/500	RRID:AB_141607 / https://www.thermofisher.com/antibody/product/Donkey-anti-Mouse-IgG-H-L-Highly-Cross-Adsorbed-Secondary-Antibody-Polyclonal/A-21202
Alexa Fluor 488<br>(donkey anti-rat)	Invitrogen	#A-21208	1/500	RRID:AB_2535794 / https://www.thermofisher.com/antibody/product/Donkey-anti-Rat-IgG-H-L-Highly-Cross-Adsorbed-Secondary-Antibody-Polyclonal/A-21208
Alexa Fluor 594<br>(donkey anti-rabbit)	Invitrogen	#A-21207	1/500	RRID:AB_141637 / https://www.thermofisher.com/antibody/product/Donkey-anti-Rabbit-IgG-H-L-

				Highly-Cross-Adsorbed-Secondary-Antibody-Polyclonal/A-21207
Alexa Fluor 594 (donkey anti-rat)	Invitrogen	#A-21209	1/500	RRID:AB_2535795 / https://www.thermofisher.com/antibody/product/Donkey-anti-Rat-IgG-H-L-Highly-Cross-Adsorbed-Secondary-Antibody-Polyclonal/A-21209
Alexa Fluor 594 (donkey anti-mouse)	Invitrogen	#A-21203	1/500	RRID:AB_141633 / https://www.thermofisher.com/antibody/product/Donkey-anti-Mouse-IgG-H-L-Highly-Cross-Adsorbed-Secondary-Antibody-Polyclonal/A-21203
Alexa Fluor 594 (donkey anti-goat)	Invitrogen	#A-11058	1/500	RRID:AB_2534105 / https://www.thermofisher.com/antibody/product/Donkey-anti-Goat-IgG-H-L-Cross-Adsorbed-Secondary-Antibody-Polyclonal/A-11058
Alexa Fluor 647 (donkey anti-goat)	Invitrogen	#A-21447	1/500	RRID:AB_2535864 / https://thermofisher.com/antibody/product/Donkey-anti-Goat-IgG-H-L-Cross-Adsorbed-Secondary-Antibody-Polyclonal/A-21447
Alexa Fluor 647 (donkey anti-rat)	Invitrogen	#A78947	1/500	RRID:AB_2910635 / https://www.thermofisher.com/antibody/product/Donkey-anti-Rat-IgG-H-L-Highly-Cross-Adsorbed-Secondary-Antibody-Polyclonal/A78947
HRP (rabbit anti-goat)	Dako	#P0449	1/2000	RRID:AB_2617143 / https://www.agilent.com/en/product/specific-proteins/elisa-kits-accessories/rabbit-anti-goat-immunoglobulins-hrp-affinity-isolated-2717114
HRP (swine anti-rabbit)	Dako	#P0217	1/2000	RRID:AB_2728719 / https://www.agilent.com/en/product/specific-proteins/elisa-kits-accessories/swine-anti-rabbit-immunoglobulins-hrp-solid-phase-absorbed-2717119
IRDye 680RD (donkey anti-mouse)	LI-COR Biosciences	#925-68072	1/10000	RRID:AB_2814908 / https://www.licor.com/bio/reagents/irdye-680rd-donkey-anti-mouse-igg-secondary-antibody
IRDye 680RD (donkey anti-rabbit)	LI-COR Biosciences	#926-68073	1/10000	RRID AB_2716687 / https://www.licor.com/bio/reagents/irdye-680rd-donkey-anti-rabbit-igg-secondary-antibody

Animals

**Species**	**Source**	**Background Strain**	**Sex**	**Persistent ID / URL**
Mouse: WT (AAV9)	Janvier labs	C57BL/6JRj	Female	https://janvier-labs.com/en/fiche_produit/2_c57bl-6jrj_mouse/
Mouse: aP2-VEGF-B	(Robciuc et al., 2016)[1]	C57BL/6JRj	Both	N/A
Mouse: αMHC-VEGF-B	(Bry et al., 2010)[2]	C57BL/6JRj	Both	N/A
Mouse: Cdh5-CreER^{T2}	(Okabe et al., 2014)[3]	C57BL/6JRj	Both	N/A
Mouse: VEGFR-1TK$^{-/-}$	(Hiratsuka et al., 1998)[4]	C57BL/6JRj	Both	N/A
Mouse: VEGFR-1$^{fl/fl}$	(Ambati et al., 2006)[5]	C57BL/6JRj	Both	N/A
Mouse: VEGFR-2$^{fl/fl}$	(Hooper at al., 2009)[6]	C57BL/6JRj	Both	N/A
Mouse: Rosa26$^{LSL-TdTomato}$	Jackson laboratory #021875	C57BL/6JRj	Both	https://www.jax.org/strain/021875
Mouse: BmxCreER^{T2}	(Ehling et al., 2013)[7]	C57BL/6JRj	Both	N/A
Rat: αMHC-VEGF-B	(Bry et al., 2010)[2]	HsdBrl:WH Wistar	Both	N/A

Other (Reagents)

Description	Source	Catalog #	Persistent ID / URL
Ketamine	MSD Animal Health	#511485	https://www.oloapteekki.fi/ketaminol-vet-50-mg-ml-20-ml-inj-liuos-511485
Xylazine	Bayer	#148999	https://www.oloapteekki.fi/rompun-vet-20-mg-ml-25-ml-inj-liuos-148999
OCT	HistoLab	#45830	https://histolab.e-line.nu/en/Products/Consumables/Reagents___Fixatives/Monteringsmedel/Frysklister_OCT_Cryomount_100_ml?id=45830
4% PFA	HistoLab	#HL96753.1000	https://histolab.e-line.nu/sv/Fixeringsvatska_PFA_4___i_fosfatbuffert_(njurbiopsi)_1L?id=HL96753.1000
β-mercaptoethanol	Sigma-Aldrich	#M3148	https://www.sigmaaldrich.com/FI/en/product/sigma/m3148
Agarose	NIPPON Genetics Europe	#AG02	https://www.nippongenetics.eu/en/products/electrophoresis-dna-rna/agarose-tablets/agarose/agarose-100g
Low melting point agarose	ThermoScientific	#R0801	https://www.thermofisher.com/order/catalog/product/R0801
Tamoxifen	Sigma-Aldrich	#T5648	https://www.sigmaaldrich.com/FI/en/product/sigma/t5648
Corn oil	Sigma-Aldrich	#C8267	https://www.sigmaaldrich.com/FI/en/product/sigma/c8267
LE-lectin	Vector labs	#FL-1171-1	https://vectorlabs.com/products/fluorescein-lycopersicon-esculentum-tomato
4-OH tamoxifen	Sigma-Aldrich	#579002	https://www.sigmaaldrich.com/FI/en/product/mm/579002
Heparin	Sigma-Aldrich	#H3393-100KU	https://www.sigmaaldrich.com/FI/en/product/sial/h3393
EdU	Invitrogen	#A10044	https://www.thermofisher.com/order/catalog/product/A10044
KCl	Sigma-Aldrich	#P9541	https://www.sigmaaldrich.com/FI/en/product/sigma/p9541
Isopentane	Honeywell	#59070	https://lab.honeywell.com/shop/2-methylbutane-59070
Liquid nitrogen	Woikoski	N/A	N/A
PBS tablets	Medicago	#09-9400-100	https://www.fishersci.fi/shop/products/phosphate-buffered-saline-tablets-ph-7-4/11330439
Ethanol	Berner	N/A	N/A
Phosphotungstic acid	Sigma-Aldrich	#79690	https://www.sigmaaldrich.com/FI/en/product/sial/79690
HCl	Sigma-Aldrich	#258148	https://www.sigmaaldrich.com/FI/en/product/sigald/258148
NaCl	Honeywell	#31434	https://lab.honeywell.com/shop/sodium-chloride-31434
KH_2PO_4	Sigma-Aldrich	#P5379	https://www.sigmaaldrich.com/FI/en/product/sial/p5379
Na_2HPO_4	Fisher BioReagents	#BP332-1	https://www.fishersci.com/shop/products/sodium-phosphate-dibasic-anhydrous-white-granules-powder-fisher-bioreagents/BP332500
$MgSO_4$	Sigma-Aldrich	#434183	https://www.sigmaaldrich.com/FI/en/product/aldrich/434183
$NaHCO_3$	Sigma-Aldrich	#S8875	https://www.sigmaaldrich.com/FI/en/product/sigald/s8875
$KHCO_3$	Sigma-Aldrich	#237205	https://www.sigmaaldrich.com/FI/en/product/sigald/237205
HEPES	Fisher BioReagents	#BP3101	https://www.fishersci.com/shop/products/hepes-fine-white-crystals-molecular-biology-fisher-bioreagents/BP3101
Taurine	Sigma Aldrich	#T8691	https://www.sigmaaldrich.com/FI/en/product/sigma/t8691
Tris	Sigma-Aldrich	#T1503	https://www.sigmaaldrich.com/FI/en/product/sigma/t1503
EDTA	Sigma-Aldrich	#E5134	https://www.sigmaaldrich.com/FI/en/product/sigma/e5134
Tween20	Fisher BioReagents	#BP337-100	https://fishersci.com/shop/products/tween-20-fisher-bioreagents/BP337100
BSA	Sigma-Aldrich	#A9647	https://www.sigmaaldrich.com/FI/en/product/sigma/a9647
Donkey serum	Biowest	#S2170	https://biowest.net/s2170-donkey-serum/

Triton X-100	Fisher bioreagents	#BP151-500	https://www.fishersci.ca/shop/products/triton-x-100-electrophoresis-fisher-bioreagents-2/bp151500
NP-40	Sigma-Aldrich	#74385	https://www.sigmaaldrich.com/FI/en/product/sigma/74385
Na-deoxycholate	Sigma-Aldrich	#30970	https://www.sigmaaldrich.com/FI/en/product/sigma/30970
Glycerol	Fisher bioreagents	#BP229-1	https://www.fishersci.com/shop/products/glycerol-molecular-biology-fisher-bioreagents/BP2291
$MgCl_2$	Sigma-Aldrich	#31413	https://www.sigmaaldrich.com/FI/en/product/sial/31413
EGTA	Thermo Scientific Chemicals	#A16086	https://www.thermofisher.com/order/catalog/product/A16086.18
$Na_4P_2O_7$	Sigma-Aldrich	#P8010	https://www.sigmaaldrich.com/FI/en/product/aldrich/p8010
NaF	Honeywell	#71522	https://lab.honeywell.com/shop/sodium-fluoride-71522
NaOH	Fisher Chemical	#S318-1	https://www.fishersci.com/shop/products/sodium-hydroxide-pellets-certified-acs-fisher-chemical-7/S3181
Collagenase type II	Worthington	#CLS-2	https://www.worthington-biochem.com/products/collagenase
Trisure reagent	Bioline	#BIO-38032	https://www.bioline.com/mwdownloads/download/link/id/953/trisure_pro-
Hoechst nuclear dye	Invitrogen	#H3570	https://www.thermofisher.com/order/catalog/product/H3570
Prolong Gold Mounting Medium	Invitrogen	#P36930	https://www.thermofisher.com/order/catalog/product/P36930
Masson's Trichrome	Sigma-Aldrich	#HT15-1KT	https://www.sigmaaldrich.com/FI/en/product/sigma/ht15
Vectashield	Vector Laboratories	#NC9265087	https://www.fishersci.com/shop/products/vectra-shield-mounting-medium/NC9265087
Streptavidin-HRP	R&D Systems	#DY998	https://www.rndsystems.com/products/streptavidin-hrp_dy998
TMB	Sigma-Aldrich	#T4444	https://www.sigmaaldrich.com/FI/en/product/sigma/t4444
Recombinant mouse VEGF-B_{167} protein	R&D Systems	#2595-VE	https://www.rndsystems.com/products/recombinant-mouse-vegf-b-167-protein_2595-ve
Recombinant mouse VEGF-B_{186} protein	R&D Systems	#767-VE	https://www.rndsystems.com/products/recombinant-mouse-vegf-b-186-protein_767-ve
Recombinant mouse VEGF-B_{186} protein (ELISA)	Novus Biologicals	#767-VE/CF	https://www.novusbio.com/products/recombinant-mouse-vegf-b-186-protein-cf_767-ve-cf
EASYpack protease inhibitors	Roche	#04693132001	https://www.sigmaaldrich.com/FI/en/product/roche/04693132001
EASYpack phosphor-protease inhibitors	Roche	#04906837001	https://www.sigmaaldrich.com/FI/en/product/roche/phossro
SuperSignal West Femto Maximum Sensitivity Substrate	Thermo Scientifc	#34096	https://www.thermofisher.com/order/catalog/product/34096
Protein Ladder	Thermo Scientific	#26616	https://www.thermofisher.com/order/catalog/product/26616

Other (Critical commercial components / Assays)

Description	Source	Catalog #	Persistent ID / URL
Zirconium oxide bead tubes	Next Advance Inc.	#MB2ZO15	N/A
Nucleospin RNA II Kit	Macherey-Nagel	#740984	https://www.mn-net.com/nucleospin-rna-plus-mini-kit-for-rna-purification-with-dna-removal-column-740984.50
High-Capacity cDNA Reverse Transcription Kit	Applied Biosystems	#10400745	https://www.thermofisher.com/order/catalog/product/4368814
FastStart Universal SYBR Green Master kit	Roche	#4913914001	https://www.sigmaaldrich.com/FI/en/product/roche/fsusgmmro
KAPA2G Fast Hotstart genotyping Mix	Roche	#2GFHSGKB	https://www.sigmaaldrich.com/FI/en/product/roche/2gfhsgkb
Weigert´s iron hematoxylin kit	Sigma-Aldrich	#1.15973.0002	https://www.sigmaaldrich.com/FI/en/product/mm/115973
Bicinchoninic acid (BCA) protein assay	Thermo Scientific	#23225	https://www.thermofisher.com/order/catalog/product/23225
Novex WedgeWell 4–20% TRIS-Glycine gel	Invitrogen	#XP04205BOX	https://www.thermofisher.com/order/catalog/product/XP04205BOX
ImmobilonFL PVDF membranes	Merck Millipore	#IPFL00010	https://www.merckmillipore.com/FI/en/product/Immobilon-FL-PVDF-Membrane,MM_NF-IPFL00010?ReferrerURL=https%3A%2F%2Fwww.google.com%2F
Monarch Genomic DNA Purification Kit	New England BioLabs	#T3010L	https://www.neb.com/en/products/t3010-monarch-genomic-dna-purification-kit#Product%20Information
Click-iT™ EdU Cell Proliferation Kit for Imaging	Invitrogen / Invitrogen	#C10337 / #C10339	https://www.thermofisher.com/order/catalog/product/C10337 / https://www.thermofisher.com/order/catalog/product/C10339

Other (Resources / Tools)

Description	Source	Persistent ID / URL
Tissue-tek VIP5 Jr.	Sakura	https://www.gmi-inc.com/product/sakura-tissue-tek-vip-5-vacuum-infiltration-processor/
Nanodrop ND-1000 Spectrophotometer	Thermo Fisher Scientific	RRID:SCR_016517
C1000 thermal cycler	BIO-RAD	RRID:SCR_019688
CryoStar NX70 HOMVPD	Thermo Fisher Scientific	https://www.fishersci.se/shop/products/cryostar-nx70-cryostat/p-4529962
Leica VT1000 S vibrating blade microtome	Leica Biosystems	RRID:SCR_016495
Leica Stellaris 8 FALCON/DLS	Leica Biosystems	RRID:SCR_024664 /
Leica TCS SP8XI	Leica Biosystems	https://www.leica-microsystems.com/products/confocal-microscopes/p/leica-tcs-sp8-x/
LI-COR Odyssey Fc	LI-COR Biosciences	RRID:SCR_023227
Bruker SkyScan 1272	Bruker	https://www.bruker.com/en/products-and-solutions/diffractometers-and-x-ray-microscopes/3d-x-ray-microscopes/skyscan-1272.html
LUNA automated cell counter	Logos Biosystems	https://logosbio.com/luna-ll/
Illumina NovaSeq 6000 system S2 flow cell	Illumina, Inc.	RRID:SCR_016387
EnSight Multimode plate reader	PerkinElmer	https://www.revvity.com/fi-en/product/ensight-instrument-hh34000000
S3e cell sorter	BIO-RAD	RRID:SCR_019710
Vevo 2100 Ultrasound system	FujiFilm VisualSonics Inc.	RRID:SCR_015816

Other (Software)

Description	Source	Persistent ID / URL
ImageStudio Lite (Version 5.2.5)	LI-COR Biosciences	RRID:SCR_013715 / https://www.licor.com/bio/image-studio/
NRecon (1.7.1.0)	Micro Photonics Inc.	N/A / https://www.microphotonics.com/micro-ct-systems/nrecon-reconstruction-software/
MeshLab (2022.02 software)	ISTI - CNR	N/A / https://www.meshlab.net/
Matlab	MathWorks	RRID:SCR_001622 / http://www.mathworks.com/products/matlab/
Rstudio (Seurat v4.4.0 R package)	Posit, PBC	RRID:SCR_000432 / https://posit.co/download/rstudio-desktop/

Excel	Microsoft	RRID:SCR_016137 / https://www.microsoft.com/en-gb/
Photoshop	Adobe	RRID:SCR_014199 / https://www.adobe.com/products/photoshop
Illustrator	Adobe	RRID:SCR_010279 / http://www.adobe.com/products/illustrator
PRISM (10.2.0)	GraphPad	RRID:SCR_002798 / https://www.graphpad.com/
Imaris	Oxford Instruments	RRID:SCR_007370 / https://imaris.oxinst.com/packages
Zen Blue	ZEISS	RRID:SCR_013672 / https://www.zeiss.com/microscopy/en/products/software/zeiss-zen
ImageJ	NIH	RRID:SCR_003070 / https://imagej.net/software/imagej/

References

1. Robciuc MR, Kivelä R, Williams IM, de Boer JF, van Dijk TH, Elamaa H, Tigistu-Sahle F, Molotkov D, Leppänen VM, Käkelä R, Eklund L, Wasserman DH, Groen AK, Alitalo K. VEGFB/VEGFR1-Induced Expansion of Adipose Vasculature Counteracts Obesity and Related Metabolic Complications. *Cell Metab*. 2016;23:712–724.

2. Bry M, Kivelä R, Holopainen T, Anisimov A, Tammela T, Soronen J, Silvola J, Saraste A, Jeltsch M, Korpisalo P, Carmeliet P, Lemström KB, Shibuya M, Ylä-Herttuala S, Alhonen L, Mervaala E, Andersson LC, Knuuti J, Alitalo K. Vascular endothelial growth factor-B acts as a coronary growth factor in transgenic rats without inducing angiogenesis, vascular leak, or inflammation. *Circulation*. 2010;122:1725–1733.

3. Okabe K, Kobayashi S, Yamada T, Kurihara T, Tai-Nagara I, Miyamoto T, Mukouyama YS, Sato TN, Suda T, Ema M, Kubota Y. Neurons limit angiogenesis by titrating VEGF in retina. *Cell*. 2014;159:584–596.

4. Hiratsuka S, Minowa O, Kuno J, Noda T, Shibuya M. Flt-1 lacking the tyrosine kinase domain is sufficient for normal development and angiogenesis in mice. *Proc Natl Acad Sci U S A*. 1998;95:9349–9354.

5. Ambati BK, Nozaki M, Singh N, Takeda A, Jani PD, Suthar T, Albuquerque RJ, Richter E, Sakurai E, Newcomb MT, Kleinman ME, Caldwell RB, Lin Q, Ogura Y, Orecchia A, Samuelson DA, Agnew DW, St Leger J, Green WR, Mahasreshti PJ, Curiel DT, Kwan D, Marsh H, Ikeda S, Leiper LJ, Collinson JM, Bogdanovich S, Khurana TS, Shibuya M, Baldwin ME, Ferrara N, Gerber HP, De Falco S, Witta J, Baffi JZ, Raisler BJ, Ambati J. Corneal avascularity is due to soluble VEGF receptor-1. *Nature*. 2006;443:993–997.

6. Hooper AT, Butler JM, Nolan DJ, Kranz A, Iida K, Kobayashi M, Kopp HG, Shido K, Petit I, Yanger K, James D, Witte L, Zhu Z, Wu Y, Pytowski B, Rosenwaks Z, Mittal V, Sato TN, Rafii S. Engraftment and reconstitution of hematopoiesis is dependent on VEGFR2-mediated regeneration of sinusoidal endothelial cells. *Cell Stem Cell*. 2009;4:263–274.

7. Ehling M, Adams S, Benedito R, Adams RH. Notch controls retinal blood vessel maturation and quiescence. *Development*. 2013;140:3051–3061.
