# Supplementary material for: Contribution of VEGF-B-Induced Endocardial Endothelial Cell Lineage in Physiological Versus Pathological Cardiac Hypertrophy
Source: Circ Res. 2024 Apr 24;134(11):1465–82. doi: 10.1161/CIRCRESAHA.123.324136 (PMC11542978; doi:10.1161/CIRCRESAHA.123.324136)
Supplement: Supplementary file 8 [file res-134-1465-s008.pdf]

Contribution of VEGF-B-induced endocardial endothelial cell lineage in physiological versus pathological cardiac hypertrophy
(Supplemental material)

Ibrahim Sultan[1, 2], MSc, Markus Ramste#[1, 2], MD, PhD, Pim Peletier#[1, 2], MSc, Karthik Amudhala Hemanthakumar[1, 2], PhD, Deepak Ramanujam[3, 8], PhD, Annakaisa Tirronen[4], PhD, Ylva von Wright[1,2], MSci, Salli Antila[1, 2], MD, Pipsa Saharinen[1,2], PhD, Lauri Eklund[5], PhD, Eero Mervaala[6], MD, PhD, Seppo Ylä-Herttuala[4], MD, PhD, Stefan Engelhardt[3], MD, PhD, Riikka Kivelä[1, 7], PhD, and Kari Alitalo*[1, 2], MD, PhD

[1]Wihuri Research Institute and [2]Translational Cancer Medicine Program, Faculty of Medicine, Biomedicum Helsinki, University of Helsinki, Finland

[3]Institute for Pharmacology and Toxicology, Technical University of Munich, and DZHK partner site Munich Heart Alliance, Munich, Germany

[4]A.I. Virtanen Institute, University of Eastern Finland, Kuopio, Finland

[5]Oulu Center for Cell-Matrix Research, Faculty of Biochemistry and Molecular Medicine, Biocenter Oulu, University of Oulu, Oulu, Finland

[6]Department of Pharmacology, Faculty of Medicine, University of Helsinki, Finland

[7]Stem Cells and Metabolism Research Program, Faculty of Medicine, University of Helsinki, and Faculty of Sport and Health Sciences, University of Jyväskylä, Finland

[8]RNATICS GmbH, Planegg-Martinsried, Germany.

#Equal contribution
*To whom correspondence should be addressed
Wihuri Research Institute
Haartmaninkatu 8, 00290 Helsinki, Finland
Tel: +358 2941 25510 Email: kari.alitalo@helsinki.fi

Short title: VEGF-B induced coronary endothelial cell lineage

Methods

For a detailed list of reagents, equipment, and softwares used throughout the study, please check the major resources table.

Mouse and rat models. Mice and rats were maintained in a specific pathogen free, temperature-controlled environment with 12-h light/dark cycle and *ad libitum* diet. All procedures used in animal experiments were approved by the National Animal Experiment Board according to the regulations of the European Union and Finnish national legislation. We used aP2-VEGF-B mice[1] as a model of cardiac autocrine VEGF-B signaling as the FABP4 (aP2) promoter is expressed in cardiac, but not in peripheral blood endothelial cells (ECs), thus avoiding the potentially toxic effects of high levels of VEGF-B in the systemic vasculature. The previously described αMHC-VEGF-B[2], Cdh5-CreER^{T2}[3], VEGFR-1TK$^{-/-}$[4], VEGFR-1$^{fl/fl}$[5], VEGFR-2$^{fl/fl}$[6], Rosa26$^{LSL-TdTomato}$ (Jackson Laboratory, stock no. 021875), and BmxCreER^{T2}[7] mouse lines and αMHC-VEGF-B[2] rats were used for the experiments. All mouse strains were maintained in the C57BL/6JRj background and the αMHC-VEGF-B rats were maintained in HsdBrl:WH Wistar background. For adeno-associated virus vector (AAV) gene transfer, we used weight and age matched (≥10-week-old) WT C57BL/6JRj female mice that were purchased from Janvier labs. Upon arrival, the mice were left for a minimum of one week to acclimatize prior to commencement of the experiment. All observed findings were validated in both male and female mice. We established that sex was not a confounder nor an effect modifier. Accordingly, only female data was included from AAV experiments to allow comparison with data from pregnancy experiments. All single-cell RNA sequencing (scRNA-seq) datasets were acquired from female mice to allow comparison and integration of different datasets in the study. The numbers of mice used in each experiment are indicated in each figure legend. Quantifications were performed in a blinded manner. In AAV experiments, we excluded occasional mice that did not express the vector based on cardiac lysates RT-qPCR. We also investigated αMHC-VEGF-B rats in which we made findings comparable to the αMHC-VEGF-B mice, including increased expression of VEGF-iEC marker transcripts in the adult heart and decreased VEGF-B_{167} protein from heart lysates in comparison to VEGF-B_{186}.

Tissue collection and animal sacrifice. Mice and rats were terminally anesthetized using intraperitoneal (i.p.) injection of a 3:1 mixture of ketamine (50mg/ml) and xylazine (20mg/ml). We used a total volume of 100 µls per mouse and 1ml per rat. Blood was collected from the left ventricle (LV) for serum/plasma isolation. For mice, a puncture was made in the right atrium, followed by injection of 200 µls of 40 mM KCl to the LV to stop heartbeats, followed by tissue isolation and gravimetry. Using a scalpel, the hearts were transversally divided into two equal parts. The upper part was embedded in OCT (Catalogue #45830, Histolab) and snap-frozen in 2-methylbutane containing 2% pentane cooled in liquid nitrogen. The lower half of the apex was snap-frozen in liquid nitrogen for biochemical and molecular analyses. For mouse phenotyping, approximately 50 mg tissues of interest were collected and snap-frozen in liquid nitrogen for molecular analyses. For immunohistochemical analysis of mouse and rat tissues, the tissues were isolated and fixed using 4% paraformaldehyde (PFA) in PBS overnight at 4°C, with the exception of adipose tissue, which was fixed for two days at 4°C. For paraffin embedding, the tissues were further processed using Tissue-tek VIP5 Jr. (Sakura). The hearts of mouse pups were washed with PBS and fixed for 60 min in 4% PFA at 4°C, washes with PBS and incubated overnight in 30% sucrose in PBS, then embedded in OCT blocks on dry ice and cryo-sectioned using CryoStar NX70 HOMVPD cryotome.

Isolation of cardiac cell types for RT-qPCR validation of VEGF-B expression. Cardiac ECs, cardiac fibroblasts (CFs), and CMCs were isolated as described previously[8]. The hearts were harvested and coronary arteries were perfused briefly with buffer A (113 mM NaCl, 4.7 mM KCl, 0.6 mM KH_2PO_4, 0.6 mM Na_2HPO_4, 1.2 mM $MgSO_4$, 12 mM $NaHCO_3$, 10 mM $KHCO_3$, 10 mM HEPES and 30 mM taurine) to remove blood in a retrograde manner by cannulating the aorta; then collagenase type II (Worthington) was added to dissociate the ventricular cells. The dissociated cells were allowed to sediment at 37°C for 10 min to allow CMCs to settle down as a pellet, leaving behind a non-myocyte rich supernatant, which was treated with rat anti-mouse CD16/CD32 antibodies (1:100, #BDB553142, clone 2.4G2, BD Biosciences) at 4°C for 20 min before being incubated with fluorophore-conjugated antibodies at 4°C for 30 min. The following antibodies were used: anti-CD45-FITC (1:100, #60030FI.1, clone 30-F11, STEMCELL Technologies), anti-PDGFR-PECy7 (1:100, #25-1401-82, clone APA5, Invitrogen), and anti-CD105-PE (1:100, #12-1051-82, clone MJ7/18, Invitrogen). Cell suspensions were triturated through a 40 µm Nylon cell strainer (Falcon) and then subjected to flow cytometry-based sorting using the Biorad S3e cell sorter.

RNA isolation and quantitative real-time PCR (RT-qPCR). Tissues were homogenized with Trisure reagent (#BIO-38032, Bioline) in zirconium oxide bead tubes (#MB2ZO15, Next Advance Inc.) and RNA was isolated according to manufacturer's instructions using Nucleospin RNA II Kit (#740984, Macherey-Nagel). For RNA isolation from cells, the manufacturer's instructions were followed, except that β-mercaptoethanol was added to the RA1 lysis buffer. The quality of the RNA was determined using the Nanodrop ND-1000 instrument (Thermo Fisher Scientific), and 1 µg of total RNA was used for cDNA synthesis using the High-Capacity cDNA Reverse Transcription Kit (#10400745, Applied Biosystems). RT-qPCR was performed using FastStart Universal SYBR Green Master kit (#4913914001, Roche) and BIO-RAD C1000 thermal cycler according to the standardized protocol. The Eq. 2–ΔΔCt method was used to quantify relative gene expression levels. Technical duplicates Cq values were averaged for each sample and normalized to the *Hprt* housekeeping gene. Expression levels of mRNA are presented as fold change (control group=1). All used primers are listed in **Supplemental table 4.**

Genotyping and PCR. Ear clips were isolated from the animals and subsequently boiled for 5 min in 50 mM NaOH, followed by spinning down at 13 000 rpm and using 1µl of the supernatant for PCR reaction using protocol of KAPA2G Fast Hotstart genotyping Mix (#kk5621, Kapa Biosystems). To check for the presence of either VEGF-B isoform-specific transcripts in the hearts of the aP2-VEGF-B, αMHC-VEGF-B and WT mice, separate PCR reactions were run on cardiac lysates cDNA using primers that amplify both mVEGF-B isoforms or the hVEGF-B isoforms. KAPA2G Fast Hotstart genotyping Mix (#kk5621, Kapa Biosystems) was used for amplification of the PCR products. The PCR reaction products were subjected to 3% agarose gel electrophoresis to visualize the products. All used primers are listed in **Supplemental table 4**.

Immunohistochemistry. Adult hearts that were OCT embedded without fixation were sectioned at 7 µm thickness and were left on glass slides to air dry for 20 min. The sections were then fixed using 4% PFA in PBS for 15 min at room temperature (RT), followed by washes in PBS. The pre-fixed hearts from pups were sectioned at 10 µm thickness followed by PBS washing. Paraffin-embedded tissues were sectioned at 5 µm, deparaffinized and rehydrated. Antigen retrieval was then performed using microwave boiling of the sections in

(10 mM Tris, 1 mM EDTA, and 0.05% Tween20 in PBS, pH 9.0) for 15 min total, followed by washing in PBS.

Blocking of sections was performed using immunomix (0.5%BSA, 5%donkey serum and 0.1%triton in PBS) for 60 min at RT. The sections were then incubated overnight with primary antibodies in immunomix at 4°C. The following primary antibodies were used: goat anti-mouse VEGF-B (1:250, #AF590, R&D Systems), rabbit anti-mouse FABP4 (1:200, #ab13979, Abcam), rat anti-mouse Plvap (1:150, #553849, BD Biosciences), goat anti-mouse podocalyxin (1:200, #AF1556, R&D Systems), mouse anti-mouse Dystrophin (1:200, #NCL-DYS2, Leica Biosystems), rat anti-mouse Cd31 (1:100, #553370, BD Biosciences), rabbit anti-mouse Cd45 (1:250, #10558, Abcam), goat anti-mouse Cd206 (1:1000, #AF2535, R&D Systems), rabbit anti-human Col13a1 (1:75, #HPA050392, Atlas Antibodies), mouse anti-mouse Actin α-Smooth Muscle-Cy3 conjugated (1:1000, #C6198, Sigma-Aldrich), mouse anti-human Nr2f2 (1:150, #PP-H7147-00, R&D Systems), goat anti-mouse Dll4 (1:100, # AF1389, R&D Systems), and goat anti-mouse VEGFR-2 (1:250, #AF644, R&D Systems). The following day, the sections were rinsed with PBS, then incubated with the secondary antibody and Hoechst nuclear dye (#H3570, Invitrogen) in immunomix for 60 min at RT, followed by PBS washes and mounting using prolong gold mounting medium (#P36930, Invitrogen). The following secondary antibodies from Invitrogen were used at 1:500 dilution: Alexa-fluor488 donkey anti-goat (#A-11055), Alexa-fluor488 donkey anti-rabbit (#A-21206), Alexa-fluor488 donkey anti-mouse (#A-21202), Alexa-fluor488 donkey anti-rat (#A-21208), Alexa-fluor594 donkey anti-rabbit (#A-21207), Alexa-fluor594 donkey anti-rat (#A-21209), Alexa-fluor594 donkey anti-mouse (#A-21203), Alexa-fluor647 donkey anti-goat (#A-21447), and Alexa-fluor647 donkey anti-rat (#A78947). For validation of antibody specificity, only the secondary antibody was used as a control in order to distinguish genuine target staining from background. All immunohistochemical experiments included known negative and positive internal staining controls to validate the specificity. Masson trichrome staining was performed on cardiac cryosections using instructions provided by the manufacturer (#HT15-1KT, Sigma-Aldrich). Counterstaining was performed using Weigert´s iron hematoxylin kit (#1.15973.0002, Sigma-Aldrich). The immunohistochemical stainings shown in the figures are the most representative of all the images captured from all samples acquired from the mouse cohorts used in the specified experiment.

Whole mount staining and confocal microscopy. The hearts were perfused with PBS followed by isolation and fixation overnight in 4% PFA at 4°C. On the following day, the hearts were rinsed and embedded in low melting point agarose and sectioned at 200 µm thickness using Leica VT1000 S vibrating blade microtome. Blocking of cardiac thick sections was performed for 1 h at RT using immunomix, followed by incubation with the primary antibodies o.n. at 4°C. On the following day, the heart sections were rinsed for 2 h in PBS, followed by addition of secondary antibodies and Hoechst (#H3570, Invitrogen) o.n. at 4°C. The samples were finally washed for 2 h in PBS and mounted in Vectashield (Vector Laboratories NC9265087). Imaging was performed in the Biomedicum Imaging Unit (BIU) of the University of Helsinki, using Leica Stellaris 8 FALCON/DLS or Leica TCS SP8Xl. Images were acquired with optimal z-stack step size. All images were acquired using sequential scanning and the value of 2 for averaging. The following primary antibodies were used for staining: goat anti-mouse podocalyxin (1:200, #AF1556, R&D Systems), rat anti-mouse Cd24 (1:500, #14-0242-82, Invitrogen), rabbit anti-mouse Plvap (1:100, #82489, Cell Signaling), goat anti-RFP (1:250, # 200-101-379, Rockland) and the following secondary antibodies were: Alexa-fluor488 donkey anti-goat (#A-11055), Alexa-fluor488 donkey anti-rabbit (#A-21206), Alexa-fluor594

donkey anti-goat (#A-11058), and Alexa-fluor594 donkey anti-rat (#A-21209). For validation of antibody specificity, only the secondary antibody was used as a control in order to distinguish genuine target staining from background. All immunohistochemical experiments included known negative and positive internal staining controls to validate the specificity. The images presented in the figures are the most representative images or tile scans that were acquired from the mouse cohorts used in each experiment.

Protein isolation and Western blotting analysis. Hearts were lysed in RIPA buffer (50 mM Tris pH 7.4, 1% NP-40, 0.25% Na-deoxycholate, 150 mM NaCl, 1mM EDTA). ECs were lysed in PLCLB buffer (50 mM HEPES, 150 mM NaCl, 10% glycerol, 1% TritonX-100, 1.5 mM $MgCl_2$, 1 mM EGTA, 10 mM $Na_4P_2O_7$, 100 mM NaF). Both buffers were supplemented with EASYpack protease and phospho-protease inhibitors (#04693132001 and #04906837001, Roche). Bicinchoninic acid (BCA) protein assay (#23225, Thermo Scientific) was used to determine the total protein content. The reaction was detected using EnSight Multimode plate reader. 25 µg from the heart lysates, and 10 µg from ECs were loaded to Novex WedgeWell 4–20% TRIS-Glycine gel (#XP04205BOX, Invitrogen), electrophoresed, and blotted to ImmobilonFL PVDF membranes (#IPFL00010, Merck Millipore) and ran in reduced conditions. We used PageRuler™ Prestained Protein Ladder (#26616, Thermo Scientific) to assist in size determination of the proteins of interest. However, we noted that in some cases correlation with the ladder exhibited slight shift from the literature-established sizes of the detected proteins. Detection of proteins was performed using the following primary antibodies: goat anti-mouse VEGF-B (1:1000, #AF590, R&D Systems), goat anti-human VEGF-B (1:1000, #AF751, R&D Systems), goat anti-mouse VEGFR-1 (1:1000, #AF471, R&D Systems), rabbit anti-human pVEGFR-1 (Y1213) (1:1000, #AF4170, R&D Systems), goat anti-mouse VEGFR-2 (1:1000, #AF644, R&D Systems), goat anti-mouse/rat Nrp-1 (1:1000, #AF566, R&D Systems), rabbit anti-mouse p-AKT (Ser473) (1:1000, #9271, Cell Signaling), rabbit anti-mouse AKT (1:1000, #9272, Cell Signaling), rabbit anti-mouse phospho-p44/42 MAPK (Erk1/2) (Thr202/Tyr204) (1:1000, #9101, Cell Signaling), rabbit anti-mouse p44/42 MAPK (Erk1/2) (1:1000, #9102, Cell Signaling), rabbit anti-mouse β-actin (1:10000, #4967, Cell Signaling), and mouse anti-HSC70 (1:10000, #SC-7298, Santa Cruz Biotechnology). The following secondary antibodies were used to probe the blots: HRP-labeled rabbit anti-goat (1:2000, #P0449, Dako) and HRP-labeled swine anti-rabbit (1:2000, #P0217, Dako). The blots were visualized with SuperSignal West Femto Maximum Sensitivity Substrate (#34096, ThermoFischer Scientific). HSC70 and β-actin were probed consecutively using IRDye 680RD donkey anti-mouse IgG (1:10000, #925-68024, LI-COR Biosciences) and IRDye 680RD donkey anti-rabbit IgG (1:10000, #926-68073, LI-COR Biosciences) and detected using LI-COR Odyssey Fc (LI-COR Biosciences). ImageStudio Lite (Version 5.2.5; LI-COR Biosciences) was used for densitometric analysis of the blots. Finally, all values were normalized to the sample loading control (HSC70/β-actin). The blots presented in the figures are representative of the results that are the most similar to the mean value. All uncropped blots are included in the supplemental material.

Micro-computed tomography (µCT). P0 hearts were obtained from the aP2-VEGF-B pups and their surviving WT littermates right after birth. The hearts were rinsed with PBS, then incubated with 5 units/ml heparin for 15 min to prevent blood clotting within the heart. The hearts were washed 3 times, 5 min each, with PBS, then fixed in 4% PFA for 4 h at RT with shaking, followed by washing with PBS 3 times for 5 min each and dehydrated for 2 h in each of 10%, 30%, 50%, and 70% ethanol in PBS. Finally, the hearts were stained with 0.3% phosphotungstic acid in 70% ethanol in PBS for 48 h then were immersed in 0.5 ml Eppendorf

tubes filled with 4% low melting point agarose. After agarose solidification, a few drops of 70% ethanol were added on top of the agarose to prevent cracking. Bruker SkyScan 1272 was used for scanning the hearts using 0.25 mm aluminum filter. Flat field correction was adjusted before scanning. Camera binning was set to 1x1 and image pixel (px) size to 2.2 µm. The rotation step was set to 0.2º within the total of 180º and frame averaging was set to 6. TIFF images were acquired in 16-bit depth and NRecon 1.7.1.0 software was used to reconstruct the scanned hearts. The hearts were post-aligned in relation to reference scan. Ring Artifact Correction was set as required. Smoothing and Beam Hardening Correction were not used. The images were reconstructed using MeshLab 2022.02 software.

Endothelial cell isolation and single-cell sequencing. Cardiac ECs were isolated from adult mice as described previously[9]. Hearts from 2-3 mice per condition/genotype were digested and pooled, and subsequently treated as 1 sample. The viability of the FAC-sorted cardiac ECs/cells was determined using an automated cell counter (Luna, Logos Biosystems). The cells were loaded into Chromium Next GEM Single Cell 3' Chip v3.1 (Dual index) (10x Genomics, Pleasanton, CA) and gel beads in emulsion (GEM) generation was performed aiming at 10 000 cell captures/sample. Subsequent cDNA purification, amplification (11 cycles), and library construction (sample index PCR 12 cycles for WT-EC and αMHC-VEGF-B-EC samples, 13 cycles for aP2-VEGF-B-SVF and αMHC-VEGF-B-SVF samples, and 14 cycles for all other samples) was performed as instructed. Sequencing of sample libraries was performed using the Illumina NovaSeq 6000 system S2 flow cell (Illumina) with the following read lengths: 28bp (Read 1), 10bp (i7 Index), 10 bp (i5 Index), and 90 bp (Read 2) resulting in 41 594 mean reads/cell for the WT-EC sample, 41 216 mean reads/cell for the aP2-VEGF-B-EC sample, 38 608 mean reads/cell for the αMHC-VEGF-B-EC sample, 53 568 mean reads/cell for the WT-SVF sample, 45 987 mean reads/cell for the aP2-VEGF-B-SVF sample, 46 834 mean reads/cell for the αMHC-VEGF-B-SVF sample, 55 247 mean reads/cell for the WT pregnant sample, 70 868 mean reads/cell for the αMHC-VEGF-B pregnant sample, 34 496 mean reads/cell for the WT post-delivery sample, 38 566 mean reads/cell for the αMHC-VEGF-B post-delivery sample, 53 785 mean reads/cell for the WT MI sample, and 53 372 mean reads/cell for the αMHC-VEGF-B MI sample. Cellranger 3' RNAseq pipeline analysis of the sequencing data was run using 10x Genomics Cell Ranger v3.1.0 count and aggr pipelines. Reads were aligned against mouse genome mm10 (refdata-cellranger-mm10-3.0.0). Seurat v4.4.0 R package was used for quality control, filtering, and analysis of data. Filtering was performed based on number of detected genes, percentage of mitochondrial genes, and number of counts per gene. With the exception of cardiac TG SVF samples, all datasets were filtered to exclude cells with less than 500 or more than 4 000 detected genes, and cells with more than 5% of mitochondrial genes. Cardiac TG SVF filtering parameters were set to exclude cells with less than 500 or more than 6 000 detected genes, and cells with more than 10% of mitochondrial genes. Dataset gene normalization was performed using the "logNormalize" method on a log scale of 10 000. Cell cycle scores were calculated using the published scoring table of cell cycle genes[10]. The top 2 000 variably expressed genes were used for scaling of datasets. Clustering was performed at a resolution of 0.5 for all datasets, except TG SVF (0.8 resolution), and Uniform Manifold Approximation and Projection (UMAP) function was used to visualize the data in two-dimensional space[11]. Identification of the clusters was done using the "FindConservedMarkers" function, followed by sub-clustering to exclude damaged cells and contaminating cell populations. Within each analysis, the integrated dataset was downsampled to analyze equal number of cells: 8381 cells for TG VEGF-B-EC dataset, 6851 cells for TG-VEGF-B-SVF dataset, 9902 cells for the pregnancy

dataset, and 8902 cells for the MI dataset. For EC comparisons in the TG SVF dataset, we subsetted ECs from the original Seurat object followed by downsampling to 5385 to ensure equal number of ECs across all 3 groups. After downsampling, the “FindMarkers” function was used to determine differential gene expression (DEG) between defined samples using the default Wilcoxon rank-sum test. Annotation of the clusters was performed based on earlier publications[12–15] and the panglaoDB database[16]. A variety of Seurat-embedded functions and ggplot2-based functions were used to generate plots for data visualization. The “DimPlot()” function was used to plot UMAPs presented in the study. The “EnhancedVolcano” function was used to plot DEG comparing TG versus WT samples. For generation of heatmaps we used the “DoHeatmap()” function, while dot plots were generated using the “DotPlot()” function. Both “DoHeatmap()” and “DotPlot()” functions use the log-normalized and scaled data stored in the Seurat object in the slot named “scale.data”. For generation of violin plots and feature plots we used the “VlnPlot()” and “FeaturePlot()” functions, respectively. All sequencing datasets are available from the GEO database under accession number GSE261561.

Gene deletion in adult mice. Cre-mediated gene deletion was induced by giving oral gavage tamoxifen (Sigma-Aldrich) dissolved in corn oil to mice at a single daily dose of 2 mg for 5 consecutive days.

***In vivo* labelling of vasculature using Lycopersicon esculentum (Tomato) Lectin.** Mice were anesthetized using 50 µls i.p injection of 3:1 mixture of ketamine (50mg/ml) and xylazine (20mg/ml), followed by administration of 100 µg of fluorescent LE-lectin (#FL-1171-1, Vector labs) by tail vein injection. The LE-lectin was allowed to circulate for 10 min, after which the mouse was sacrificed, heart collected, OCT embedded, and sectioned.

Lineage tracing and gene deletion in pups. BmxCreER^{T2}; Rosa26-tdTomato; αMHC-VEGF-B and BmxCreER^{T2}; Rosa26-tdTomato littermate pups were injected on postnatal day 1 (P1) or postnatal day 5 (P5) with 2.5 µls of 4-OH tamoxifen (#579002, Sigma-Aldrich, stock concentration = 25mg/ml) into the stomach (milk-line) to induce labelling for lineage tracing. The injections were administered twice with a 12 h interval. For gene deletion in BmxCreER^{T2}; Rosa26-tdTomato; αMHC-VEGF-B; VEGFR-2$^{fl/fl}$ and BmxCreER^{T2}; Rosa26-tdTomato; αMHC-VEGF-B; VEGFR-2$^{wt/wt}$ littermates, the pups were injected with 2.5 µls of 4-OH tamoxifen once a day at P1-P3.

Use of adeno-associated viral vectors. AAVs were produced as previously described[17]. Lab-generated AAV of serotype 9 (AAV9) encoding mVEGF-B$_{167}$, mVEGF-B$_{186}$, or having a scrambled control sequence downstream of CAG promoter was injected i.p. to adult mice at the dose of 2.2 x 10^{11}, 6.6 x 10^{11}, or 19.8 x 10^{11} AAV9 particles and the recipient mice were analyzed at the timepoints mentioned in the results. For quantification of AAV9 in mouse tissues, DNA was extracted using Monarch Genomic DNA Purification Kit (#T3010L, New England BioLabs) and subjected to qPCR detection of the woodchuck posttranscriptional regulatory element (WPRE), with normalization to the lowest value (Intestine).

5-Ethynyl-2’deoxyuridine (**EdU**) **labeling.** EdU was dissolved in sterile PBS and administered i.p. to the mice at a total daily dose of 40 µg/g of body weight delivered in two separate doses/day at 12 h intervals, for three consecutive days prior to mouse sacrifice. EdU detection was performed on cardiac cryosections according to manufacturer’s instructions using Click-iT™ EdU Cell Proliferation Kit for Imaging (#C10337, C10339).

Enzyme-linked immunosorbent assay (ELISA). To measure mouse VEGF-B_{186} levels in sera, ELISA was developed using R&D Systems proteins and antibodies[1]. The antibodies used for capture and detection, consecutively, were: mVEGF-B (1:250, #AF590) and biotinylated-mVEGF-B_{186} (1:500, #BAF767). For preparation of standard curve, recombinant VEGF-B_{186} protein (#767-VE/CF) was used at 25 ng/ml and seven 2-fold serial dilutions. Streptavidin-HRP (#890803, R&D Systems), and TMB (#T4444, Sigma) were used for signal detection and 1 M HCl to stop the reaction. Absorbance was measured at 450 nm.

Randomization. In AAV experiments, we used random-numbers table to assign groups. We ordered mice from Janvier mouse supplier that were age, weight and gender matched. Upon mouse arrival, the mice were assigned running numbers to give them mouse IDs. Mouse assignment to the mouse ID was randomly done. We used the running number to allocate the mice within an experimental AAV group, however, the AAV vector type assignment to the mouse group was done randomly. In experiments dealing with transgenic mice, we performed genotyping and allocated the mice to wildtype (WT) and transgenic (TG) groups. The allocation of experimental groups was performed randomly while taking in consideration the age and sex matching.

Blinding. Formulation of research plans and subsequent experiment documentation was performed by the first author and was supervised by the last author. Access to the research plans and experimental files revealing the allocated groups was provided to all authors and technicians who participated in data acquisition and analysis only after data acquisition and analysis was finalized. Throughout the whole study, all experimental analysis as quantifications and molecular analysis was performed blindly followed by assigning the obtained results from the analysis to the groups.

Inclusion and exclusion criteria. We set the initial criteria for inclusion and exclusion. In all experiments dealing with transgenic mice, we included age-matched wildtype (WT) littermates as the control group. We aimed to have matching numbers of mice in each experimental group. However, in the case that more mice were available from one group, we proceeded to include all the available mice. Mice that died because of the transgene or operation were not included in the analysis. In RT-qPCR, samples that showed to be outliers in terms of housekeeping genes expression were excluded from the analysis. Immunohistochemical stainings that failed initially and upon repetition were excluded from the quantifications analysis due to possible sample preparation problems. In lineage tracing experiments, mice that did not show expression of the reporter were excluded from the analysis. In gene deletion experiments, mice that did not show gene deletion were excluded from the analysis (most likely due to failure in tamoxifen administration). In AAV experiments, mice that did not show expression of the AAV-delivered gene at RNA and/or protein level in the target tissue, were excluded from the analysis. In some cases, we confirmed overexpression in the liver but could not confirm overexpression in the heart. In such case, we excluded heart from the analysis but still maintained the liver (Liver cells highly transfected by AAV).

References

1. Robciuc MR, Kivelä R, Williams IM, de Boer JF, van Dijk TH, Elamaa H, Tigistu-Sahle F, Molotkov D, Leppänen VM, Käkelä R, Eklund L, Wasserman DH, Groen AK, Alitalo K. VEGFB/VEGFR1-Induced Expansion of Adipose Vasculature Counteracts Obesity and Related Metabolic Complications. *Cell Metab*. 2016;23:712–724.

2. Bry M, Kivelä R, Holopainen T, Anisimov A, Tammela T, Soronen J, Silvola J, Saraste

A, Jeltsch M, Korpisalo P, Carmeliet P, Lemström KB, Shibuya M, Ylä-Herttuala S, Alhonen L, Mervaala E, Andersson LC, Knuuti J, Alitalo K. Vascular endothelial growth factor-B acts as a coronary growth factor in transgenic rats without inducing angiogenesis, vascular leak, or inflammation. *Circulation*. 2010;122:1725–1733.

3. Okabe K, Kobayashi S, Yamada T, Kurihara T, Tai-Nagara I, Miyamoto T, Mukouyama YS, Sato TN, Suda T, Ema M, Kubota Y. Neurons limit angiogenesis by titrating VEGF in retina. *Cell*. 2014;159:584–596.

4. Hiratsuka S, Minowa O, Kuno J, Noda T, Shibuya M. Flt-1 lacking the tyrosine kinase domain is sufficient for normal development and angiogenesis in mice. *Proc Natl Acad Sci U S A* [Internet]. 1998 [cited 2023 Dec 6];95:9349–9354.

5. Ambati BK, Nozaki M, Singh N, Takeda A, Jani PD, Suthar T, Albuquerque RJ, Richter E, Sakurai E, Newcomb MT, Kleinman ME, Caldwell RB, Lin Q, Ogura Y, Orecchia A, Samuelson DA, Agnew DW, St Leger J, Green WR, Mahasreshti PJ, Curiel DT, Kwan D, Marsh H, Ikeda S, Leiper LJ, Collinson JM, Bogdanovich S, Khurana TS, Shibuya M, Baldwin ME, Ferrara N, Gerber HP, De Falco S, Witta J, Baffi JZ, Raisler BJ, Ambati J. Corneal avascularity is due to soluble VEGF receptor-1. *Nature* [Internet]. 2006;443:993–997.

6. Hooper AT, Butler JM, Nolan DJ, Kranz A, Iida K, Kobayashi M, Kopp HG, Shido K, Petit I, Yanger K, James D, Witte L, Zhu Z, Wu Y, Pytowski B, Rosenwaks Z, Mittal V, Sato TN, Rafii S. Engraftment and reconstitution of hematopoiesis is dependent on VEGFR2-mediated regeneration of sinusoidal endothelial cells. *Cell Stem Cell*. 2009;4:263–274.

7. Ehling M, Adams S, Benedito R, Adams RH. Notch controls retinal blood vessel maturation and quiescence. *Development* [Internet]. 2013 [cited 2023 Jun 14];140:3051–3061.

8. Ramanujam D, Sassi Y, Laggerbauer B, Engelhardt S. Viral Vector-Based Targeting of miR-21 in Cardiac Nonmyocyte Cells Reduces Pathologic Remodeling of the Heart. *Mol Ther*. 2016;24:1939–1948.

9. Hemanthakumar KA, Fang S, Anisimov A, Mäyränpää MI, Mervaala E, Kivelä R. Cardiovascular disease risk factors induce mesenchymal features and senescence in mouse cardiac endothelial cells. *Elife*. 2021;10.

10. Kowalczyk MS, Tirosh I, Heckl D, Rao TN, Dixit A, Haas BJ, Schneider RK, Wagers AJ, Ebert BL, Regev A. Single-cell RNA-seq reveals changes in cell cycle and differentiation programs upon aging of hematopoietic stem cells. *Genome Res*. 2015;25:1860–1872.

11. Becht E, McInnes L, Healy J, Dutertre CA, Kwok IWH, Ng LG, Ginhoux F, Newell EW. Dimensionality reduction for visualizing single-cell data using UMAP. *Nat Biotechnol*. 2018;

12. Räsänen M, Sultan I, Paech J, Hemanthakumar KA, Yu W, He L, Tang J, Sun Y, Hlushchuk R, Huang X, Armstrong E, Khoma OZ, Mervaala E, Djonov V, Betsholtz C, Zhou B, Kivelä R, Alitalo K. VEGF-B Promotes Endocardium-Derived Coronary Vessel Development and Cardiac Regeneration. *Circulation*. 2020;

13. Karaman S, Paavonsalo S, Heinolainen K, Lackman MH, Ranta A, Hemanthakumar KA, Kubota Y, Alitalo K. Interplay of vascular endothelial growth factor receptors in organ-specific vessel maintenance. *J Exp Med*. 2022;219.

14. Kalucka J, de Rooij L, Goveia J, Rohlenova K, Dumas SJ, Meta E, Conchinha N V, Taverna F, Teuwen LA, Veys K, García-Caballero M, Khan S, Geldhof V, Sokol L,

Chen R, Treps L, Borri M, de Zeeuw P, Dubois C, Karakach TK, Falkenberg KD, Parys M, Yin X, Vinckier S, Du Y, Fenton RA, Schoonjans L, Dewerchin M, Eelen G, Thienpont B, Lin L, Bolund L, Li X, Luo Y, Carmeliet P. Single-Cell Transcriptome Atlas of Murine Endothelial Cells. *Cell*. 2020;180:764-779.e20.

15. A single-cell transcriptomic atlas characterizes ageing tissues in the mouse. *Nature*. 2020;583:590–595.

16. Franzén O, Gan LM, Björkegren JLM. PanglaoDB: a web server for exploration of mouse and human single-cell RNA sequencing data. *Database J Biol Databases Curation* [Internet]. 2019 [cited 2024 Mar 5];2019:46.

17. Anisimov A, Alitalo A, Korpisalo P, Soronen J, Kaijalainen S, Leppänen VM, Jeltsch M, Ylä-Herttuala S, Alitalo K. Activated forms of VEGF-C and VEGF-D provide improved vascular function in skeletal muscle. *Circ Res*. 2009;104:1302–1312.

Supplemental figure legends

Supplemental figure 1. (A) Gating and sorting strategy for isolation of cardiac fibroblasts (CFs) and endothelial cells (ECs) using flow cytometry. Hearts of WT and aP2-VEGF-B mice were proteolytically digested, myocytes were discarded, and the entire non-myocyte cell suspension was stained with fluorophore-conjugated antibodies against the marked epitopes: Cd45 (leukocytes), Cd105 (ECs) and PDGFRα (CFs and pericytes). **(B)** Flow cytometry charts. The first gate for live cells was based on FSC-Area/SSC-Area (left), followed by a gate for non-leukocytes based on SSC-height and Cd45 (middle). Subsequent single cell gates were for CFs (Cd45-Cd105- PDGFRα+) and ECs (Cd45-Cd105+PDGFRα-).

Supplemental figure 2. (A) Concentration of mVEGF-B_{186} (ng/ml) in sera from adult aP2-VEGF-B, αMHC-VEGF-B and WT mice (n= 3). Note that the αMHC-VEGF-B mice express human VEGF-B transgene, while the ELISA detects only the mouse VEGF-B_{186}. *P, Kruskal-Wallis ANOVA test with Dunn's correction. **(B)** Comparison of the weights of tissues isolated from adult aP2-VEGF-B mice and their WT littermates (n= 4-6). Spleens and kidneys were weighed from a separate experiment. *P, unpaired Mann Whitney t-test. **(C, D)** Representative immunohistochemical stainings of Cd31 and dystrophin in heart cryosections from adult aP2-VEGF-B mice and their WT littermates (n= 9 WT, 7 aP2). Note the increased vessel area fraction (%) and increased CMC size (μm^2) measured from the mid-myocardium. Scale bars 50 µm. *P, unpaired two-tailed t-test with Welch correction. **(E)** Percentages of aP2-VEGF-B mice obtained from WT/WT to WT/aP2 matings at E18, P0, and in 4-weeks old mice, and those obtained from 4-weeks old mice from WT/WT to WT/αMHC matings. **(F)** Heart weight to body weight ratio at the indicated postnatal days in aP2-VEGF-B and αMHC-VEGF-B pups versus their WT littermates (For aP2 weights; n= 4 WT_P0, 3 aP2_P0, 8 WT_P7, 3 aP2_P7, 3 WT_P14, 5 aP2_P14, 3 WT_P28, 3 aP2_P28, For αMHC weights; 4 WT_P0, 4 αMHC_P0, 3WT_P7, 5 αMHC_P7, 4WT_P14, 3 αMHC_P14, 3 WT_P28, 3 αMHC_P28). *P, unpaired Mann Whitney t-test. **(G)** Representative cardiac MRI images of adult aP2-VEGF-B mice and their WT littermates. **(H)** ECG values of aP2-VEGF-B and WT littermate mice (n= 6 WT, 4 aP2). Values are represented as means ± SD. *P, unpaired Mann Whitney t-test.

Supplemental figure 3. (A, B) Representative images from cardiac *ex vivo* µCT scanning of dead aP2-VEGF-B pups and surviving WT littermates at P0. Yellow arrows point to septal defects. Scale bars 1mm.

Supplemental figure 4. (A) A heatmap showing the expression levels of cell identity transcript markers used to annotate cell clusters in the VEGF-B TG mice. The red box indicates some of the VEGFB-iECs markers. The heatmap was generated using the Seurat-embedded function “DoHeatmap()”. The average expression is plotted using log-normalized and scaled data stored in the Seurat object under the slot “scale.data”. **(B)** Bar plot showing comparison of the percentage of each cell population across samples from aP2-VEGF-B, αMHC-VEGF-B and WT mice. **(C)** Violin plots showing overlaid expression of VEGFB-iECs markers in the aP2-VEGF-B, αMHC-VEGF-B and WT samples. The single-cell datasets were generated through pooling of 3 hearts into 1 sample per group.

Supplemental figure 5. (A) UMAP plot showing clusters from Seurat integrated analysis of cardiac stromovascular fractions from aP2-VEGF-B, αMHC-VEGF-B, and WT littermate mice. **(B)** A heatmap showing the expression levels of marker transcripts used to annotate the cell clusters. The heatmap was generated using the “DoHeatmap()” function embedded in Seurat package. The average expression was plotted using log-normalized and scaled data stored in the Seurat object under the slot “scale.data”. Gene expression is presented as an average per cluster. **(C)** Bar plot showing comparison of the percentages of each cell population across the samples. **(D)** Violin plots showing overlaid expression of selected VEGFB-iEC marker transcripts in the ECs from aP2-VEGF-B, αMHC-VEGF-B and WT hearts. Note that the ECs were downsampled to have an equal number of ECs per genotype in the comparison. (**E**) Volcano plots showing the highest DEGs across all cardiac fibroblasts (CFs) from aP2-VEGF-B mice versus WT mice or αMHC-VEGF-B mice versus WT mice, respectively. Volcano plots present the values obtained from Seurat integrated DEG analysis, where avg log2FC is presented on x-axis and log10 adjusted P-value is presented on y axis. The p-value was adjusted based on Bonferroni correction using all features in the dataset. Single-cell dataset was performed through pooling of 2 hearts per genotype and subsequently ran as 1 sample per genotype. Please note that VEGFB-iECs do not cluster separately due to presence of other cell types and the set clustering resolution value, which resulted in the clustering of VEGFB-iECs with capillary ECs.

Supplemental figure 6. Representative immunohistochemical stainings of podocalyxin and Cd45 **(A)** or Cd206 **(B)** in cardiac sections from adult aP2-VEGF-B, αMHC-VEGF-B, and WT littermate mice (n= 3-4), and the corresponding quantifications. *P, Kruskal-Wallis ANOVA test with Dunn’s correction. Scale bar 20µm. Quantification of inflammation markers **(C)** and cardiac stress markers **(D)** from heart lysates of aP2-VEGF-B, αMHC-VEGF-B, and WT adult littermate mice by RT-qPCR (n= 6 WT, 5 aP2, 6 αMHC). *P, Kruskal-Wallis ANOVA test with Dunn’s correction.

Supplemental figure 7. (A) Representative immunohistochemical staining of Cd24 and podocalyxin in thick vibratome sections showing the sub-endocardial region of aP2-VEGF-B, αMHC-VEGF-B and WT mice. Scale bars 10 µm. Yellow arrows point to Plvap and Cd24 positive ECs, while red arrows point to Cd24-positive cells within the vascular lumen which escaped vascular PBS perfusion. **(B)** Quantifications of Plvap-podocalyxin double positive vessels (n= 3), and Cd24-podocalyxin double positive vessels (n= 3 WT, 4 aP2, 5 αMHC) in sub-endocardial, mid-myocardial, and sub-epicardial regions of the heart. *P, Kruskal-Wallis ANOVA test. **(C)** Quantifications of vessel lumen areas in the sub-endocardial, mid-myocardial, and sub-epicardial regions of the heart from aP2-VEGF-B, αMHC-VEGF-B and WT mice, using podocalyxin staining (n= 3 WT, 7 aP2, 10 αMHC). *P, Kruskal-Wallis ANOVA test.

Supplemental figure 8. (A) Representative immunohistochemical staining of Col13a1, Plvap, and podocalyxin in the sub-endocardial region of WT and VEGF-B TG hearts. Scale bars 50 µm. **(B)** Representative images of Plvap-stained heart sections from αMHC-VEGF-B and WT littermate mice that were perfused with fluorescent LE-lectin. Scale bars 50 µm.

Supplemental figure 9. (A) Merged feature plots from Seurat integrated analysis of aP2-VEGF-B, αMHC-VEGF-B and WT FAC-sorted cardiac ECs, showing cells expressing the capillary marker *Car4*, arterial marker *Gja4*, and venous marker *Nr2f2*. **(B)** Representative immunohistochemical stainings of Plvap and podocalyxin with SMA**,** or Nr2f2 (Coup-tfII), in heart cryosections of adult aP2-VEGF-B, αMHC-VEGF-B and WT mice. Scale bars 50 µm. **(C)** Quantification of relative amounts of VEGFB-iECs markers from aP2-VEGF-B, αMHC-VEGF-B and WT tissue lysates by RT-qPCR (n= 4 WT, 3 aP2, 4 αMHC). *P, Kruskal-Wallis ANOVA test with Dunn's correction.

Supplemental figure 10. (A) Representative immunohistochemical stainings of Dll4, SMA, and Plvap in cardiac cryosections from adult aP2-VEGF-B, αMHC-VEGF-B and WT mice. Scale bars 50 µm. **(B)** Representative immunohistochemical staining of Vegfr-2 and Cd31 in cardiac cryosections from P1 αMHC-VEGF-B pups and their WT littermates. Scale bars 20 µm. Note magenta arrows pointing to VEGFR-2 positive segments of the endocardial cell layer. **(C)** Violin plot showing expression of *Kdr* in the scRNA seq dataset obtained from FAC-sorted cardiac ECs isolated from adult aP2-VEGF-B, αMHC-VEGF-B and WT mice. The single-cell datasets were generated through pooling of 3 hearts into 1 sample per group. **(D)** Representative immunohistochemical staining of Vegfr-2 and Cd31 in cardiac cryosections from adult WT hearts. Scale bars 20 µm. Note magenta arrows pointing to VEGFR-2 positive segments of the endocardial cell layer.

Supplemental figure 11. Representative immunohistochemical stainings of Plvap and RFP in 200 µm thick cardiac sections from 4-6 weeks old BmxCreER^{T2}; Rosa26-tdTomato; αMHC-VEGF-B; VEGFR-2$^{fl/fl}$ mice and their BmxCreER^{T2}; Rosa26-tdTomato; αMHC-VEGF-B; VEGFR-2$^{wt/wt}$ littermates that were administered tamoxifen on postnatal days P1-P3. Scale bar 100µm.

Supplemental figure 12. (A) A table showing the numbers of LAD operated and survived aP2-VEGF-B, αMHC-VEGF-B and WT littermate mice. **(B)** Quantification of mVEGF-B$_{167}$ and mVEGF-B$_{186}$ transcripts from heart lysates (n= 6) and sorted cardiac ECs (n= 2) of aP2-VEGF-B and WT littermate mice by RT-qPCR. *P, unpaired two-tailed t-test with Welch correction for heart lysate mVEGF-B$_{167}$ and *P, unpaired Mann Whitney t-test for the rest. **(C)** Agarose gel electrophoresis of both mouse and human VEGF-B$_{167}$ and VEGF-B$_{186}$ PCR products using heart lysate cDNA and primers that bind to VEGF-B exon 3 and exon 7 sequences. Numbers indicate isoform intensity signal normalized to the total signal from both isoforms. Note that the mVEGF-B$_{186}$ primers also recognize the human VEGF-B$_{186}$ isoform. **(D)** WB of plasma mVEGF-B from mice after 1 week treatment with AAV9-mVEGF-B$_{167}$, AAV9-mVEGF-B$_{186}$ and AAV9-scrambled control**. (E)** WB detection of 10 ng of both VEGF-B$_{167}$ and VEGF-B$_{186}$ recombinant polypeptides using the mVEGF-B antibody. **(F)** Schematic of the AAV9 VEGF-B experiment, and RT-qPCR of AAV9 vectors (WPRE) in tissue lysates containing both genomic DNA and episomal DNA (n= 4). **(G)** RT-qPCR of mouse VEGF-B isoforms in the indicated tissue lysates after 1 (n= 3), 2 (n= 3-4), and 4 (n= 2-4 for hearts, 4 for livers) weeks of treatment with the indicated AAV9s. *P, Kruskal-Wallis ANOVA test. **(H)** WB and quantifications of

mVEGF-B from heart lysates of mice treated with the indicated AAV9s at the indicated timepoints.

Supplemental figure 13. (A) Representative images showing detected EdU and immunohistochemical staining of podocalyxin in the sub-endocardium after 1, 2, and 4 weeks of treatment with AAV9 encoding the indicated VEGF-B isoforms. Scale bars 20 µm. **(B)** Quantification of EdU positive endothelial nuclei masked by vascular staining from the indicated heart regions in mice treated with the AAVs at 1 (n= 3), 2 (n= 3-4), and 4 (n= 2-4) weeks post AAV administration. *P, Kruskal-Wallis ANOVA test. **(C)** HW/BW analysis of mice treated with the AAVs. *P, Kruskal-Wallis ANOVA test. **(D)** Cardiac ejection fraction percentage obtained from echocardiography analysis of mice before the AAV administration and at 1 and 4 weeks after administration (n= 4). *P, two-way ANOVA with Dunnett's correction.

Supplemental figure 14. (A) Western blots of the indicated polypeptides and **(B)** signal quantification from heart lysates of WT mice treated with AAV9 encoding VEGF-B_{186}, VEGF-B_{167}, or a scrambled control for two weeks (n= 4). *P, Kruskal-Wallis ANOVA test.

Supplemental figure 15 Quantification of VEGFB-iECs marker transcripts from mice treated with AAVs encoding the indicated VEGF-B isoform in comparison to a scrambled control at 1 (n= 3) **(A)**, 2 (n= 3-4) **(B)**, and 4 (n= 2-4) **(C)** weeks (*P, Kruskal-Wallis ANOVA test), and from Cdh5-CreER^{T2}; VEGFR-1$^{fl/fl}$ mice two weeks after VEGFR-1 deletion **(D)** (n= 3 VEGFR-1$^{fl/fl}$ Cre-, 8 VEGFR-1$^{fl/fl}$ Cre+, *P, unpaired Mann Whitney t-test), plus **(E)** from mice lacking the VEGFR-1 tyrosine kinase domain (n= 3 VEGFR-1 TK$^{wt/wt}$, 4 VEGFR-1 TK$^{-/-}$, *P, unpaired Mann Whitney t-test).

Supplemental figure 16. (A) Representative immunohistochemical stainings of podocalyxin and Plvap in cardiac cryosections of adult WT mice treated with 19.8*10^11vp of AAV9-VEGF-B_{186} versus AAV9-scrambled control for two weeks. Scale bars 100µm. Note that the magenta arrows point to the small expanded population of VEGFB-iECs. **(B)** Experimental timeline of Cdh5-CreER^{T2} mediated deletion of VEGFR-2 and administration of AAV9 encoding VEGF-B_{186} or the scramble control vector. **(C)** HW/BW analysis of mice with or without EC deletion of VEGFR-2 and treated with the indicated AAVs (n= 6 AAV-Scrambled + VEGFR-2$^{fl/fl}$ Cre-, 3 AAV-VEGF-B_{186} + VEGFR-2$^{fl/fl}$ Cre-, 4-5 AAV-VEGF-B_{186} + VEGFR-2$^{fl/fl}$ Cre+). *P, unpaired Mann Whitney t-test. **(D)** Quantification of VEGFB-iEC marker transcripts from the groups analyzed in **(B)**. *P, Kruskal-Wallis ANOVA test.

Supplemental tables

Supplemental table 1. Echocardiography parameters from adult αMHC-VEGF-B and WT littermate mice 2 days after LAD ligation (n= 4 WT, 3 αMHC). For each genotype, echo parameters measured at two days post-MI were compared to the basal level values. Values are represented as means ± SD. *P for values that passed normality testing, two-way ANOVA (Fischer's LSD test). *P for values that did not pass normality testing, Wilcoxon matched-pairs signed rank test using Holm-Šídák method. Annotating with (,s) refers to end-systole, while (,d) refers to end-diastole. Thickness of the intraventricular septum (IVS), thickness of the left ventricle posterior wall (LVPW), left ventricular mass (LV mass), left ventricular volume (LV vol), left ventricular internal diameter (LVID), ejection fraction (EF), and fractional shortening (FS).

Supplemental table 2. Echocardiography parameters from WT mice treated with AAV9s encoding either of the two VEGF-B isoforms in comparison to a scrambled control at 1- and 4-weeks after AAV administration (n= 4). Within each treatment, echo parameters of 1 week and 4 weeks were compared to the basal level values. *P, two-way ANOVA with Dunnett's correction. Annotating with (,s) refers to end-systole, while (,d) refers to end-diastole. Thickness of the intraventricular septum (IVS), thickness of the left ventricle posterior wall (LVPW), left ventricular mass (LV mass), left ventricular volume (LV vol), left ventricular internal diameter (LVID), ejection fraction (EF), and fractional shortening (FS).

Supplemental table 3. Summary of the statistical tests used in the experiments presented in the main and supplemental figures.

Supplemental table 4. List of oligo-primers used for qPCR and PCR.

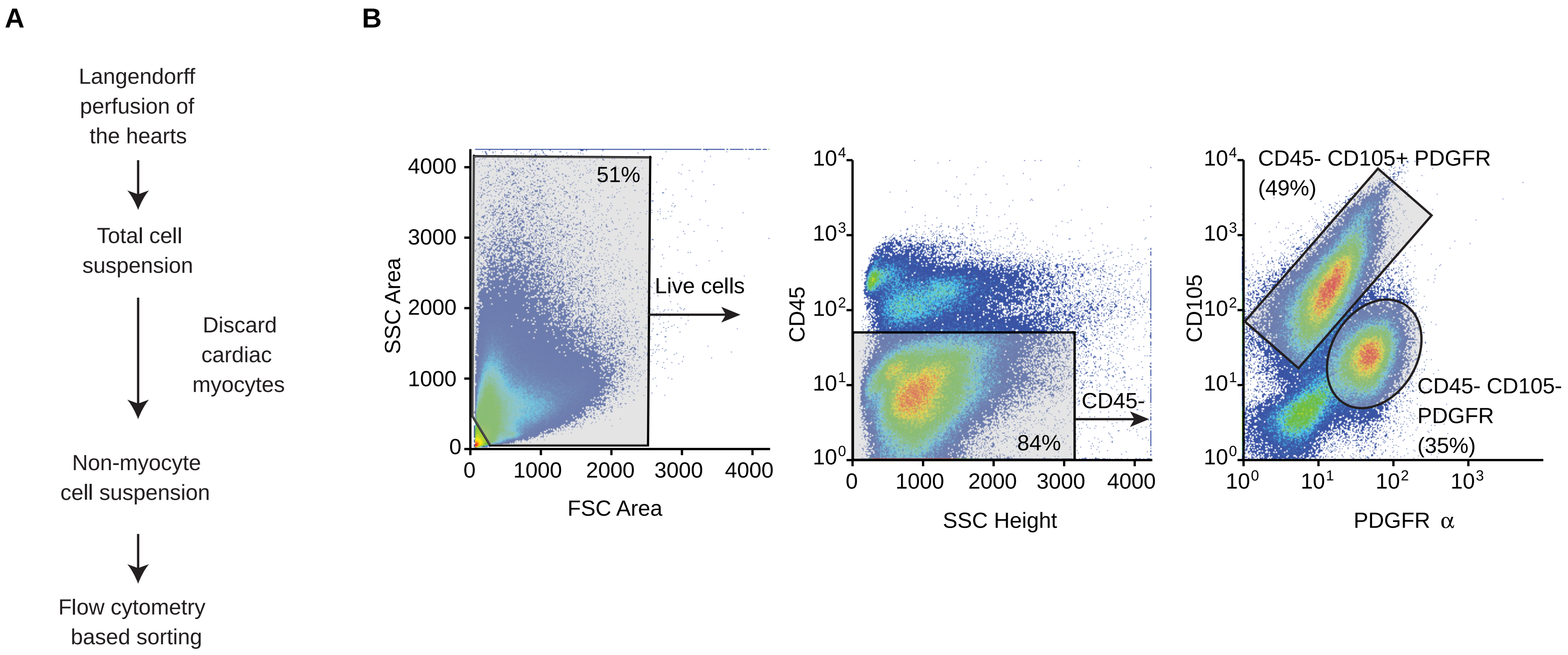

Supplemental figure 1

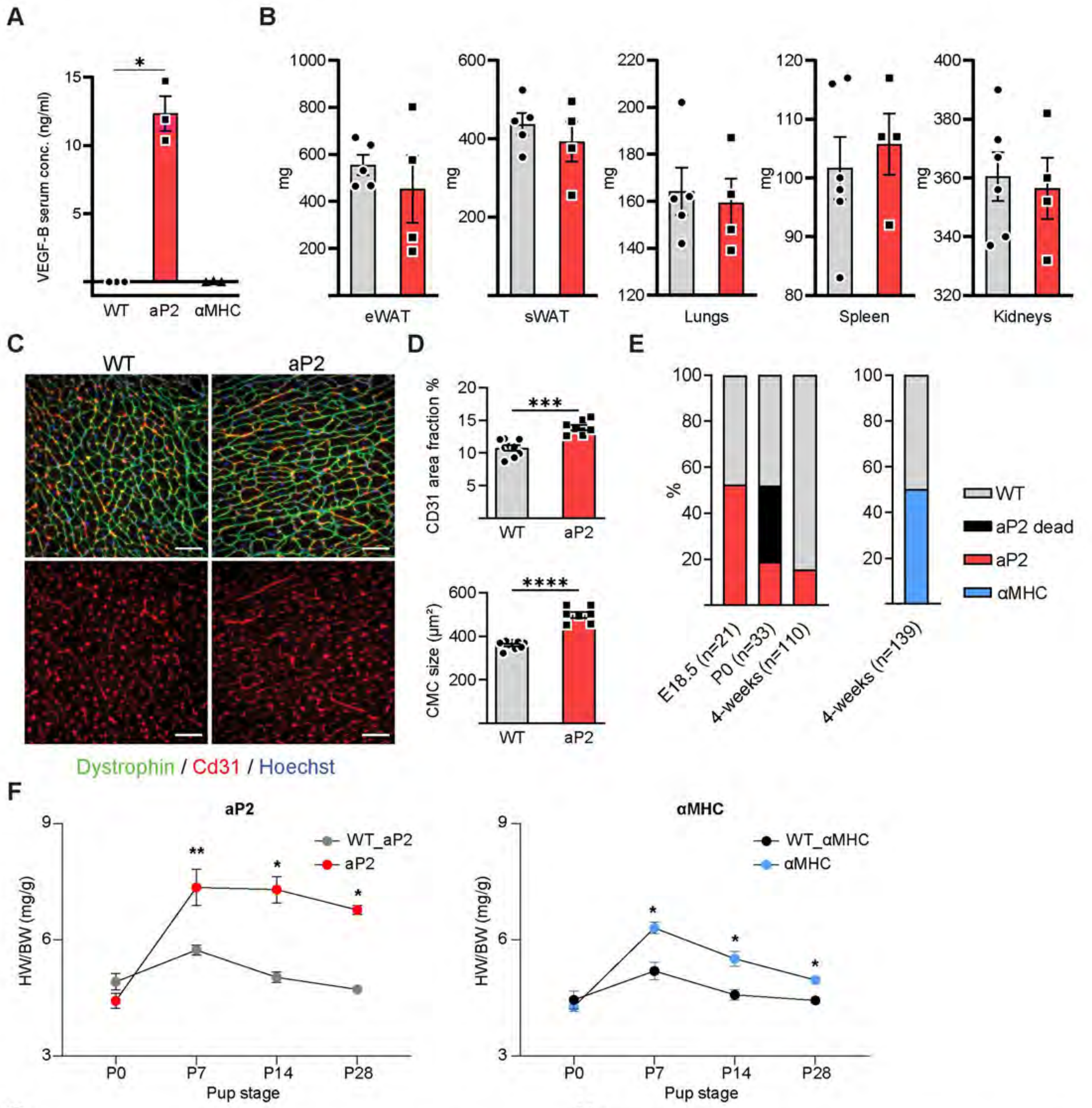

G

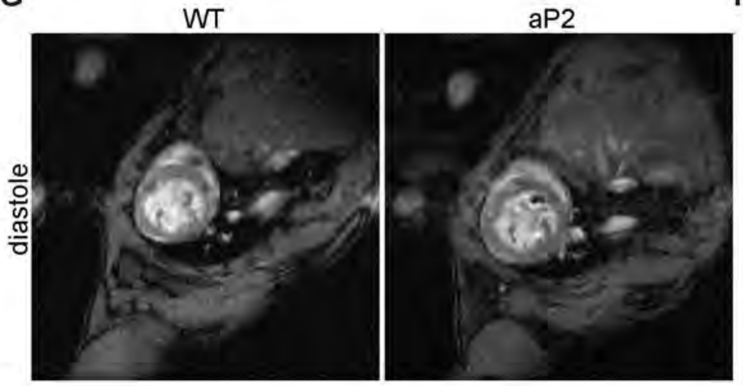

H

Parameter (unit)	WT	aP2
HR (bpm)	453.31 ± 48.82	422.16 ± 82.33
PQ (ms)	41.35 ± 2.5	46.56 ± 2.33*
QRS (ms)	10.06 ± 1.02	11.9 ± 1.16*
QTc (ms)	43.57 ± 3.14	45.66 ± 4.58
QRSp (ms)	15.02 ± 0.96	18.46 ± 2.31*
Amp P (mV)	1.01 ± 0.14	1.21 ± 0.07**
Amp R (mV)	8.56 ± 1.79	5.38 ± 2.46
Amp S (mV)	-3.67 ± 1.69	-2.84 ± 1.65

Supplemental figure 2

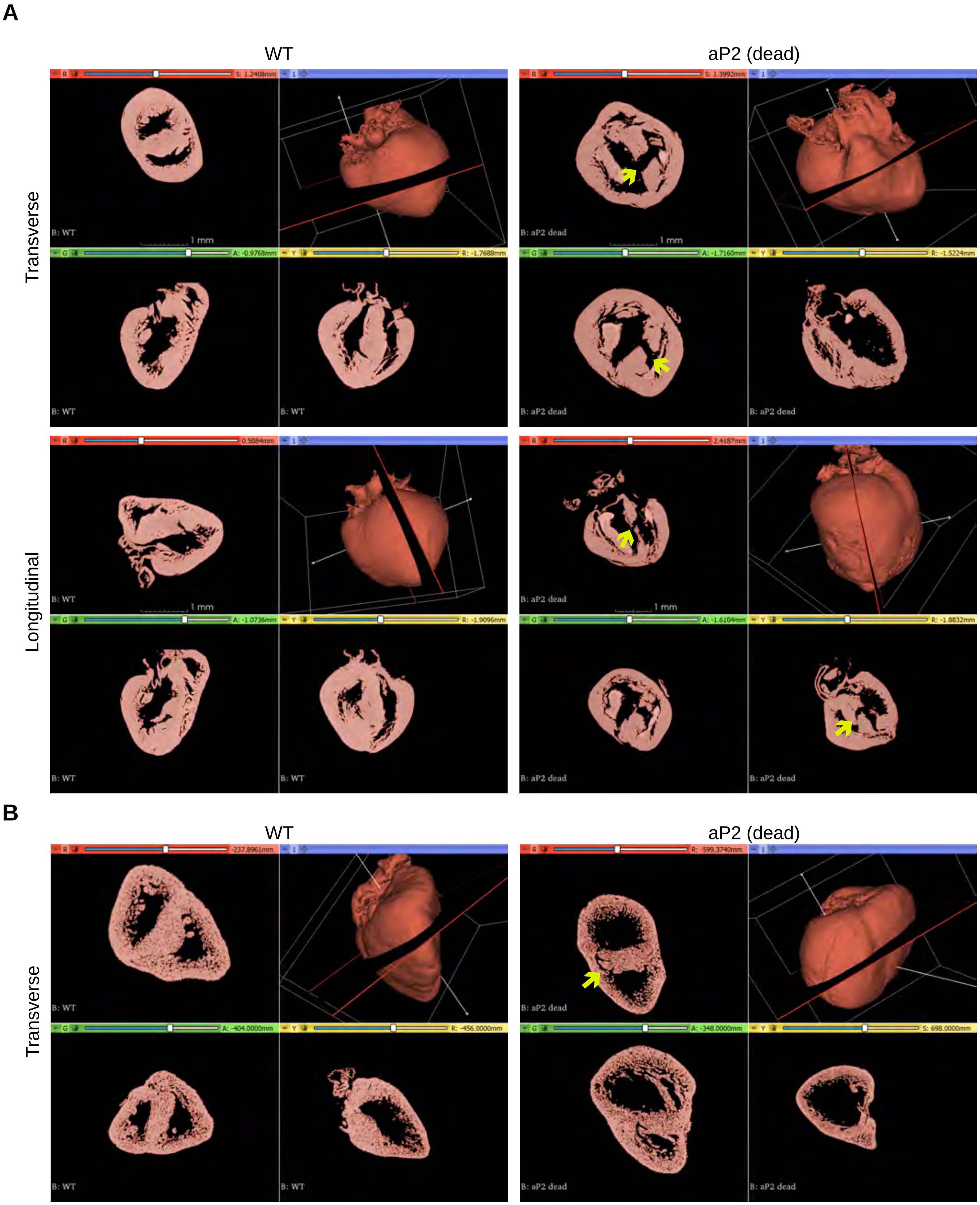

Supplemental figure 3

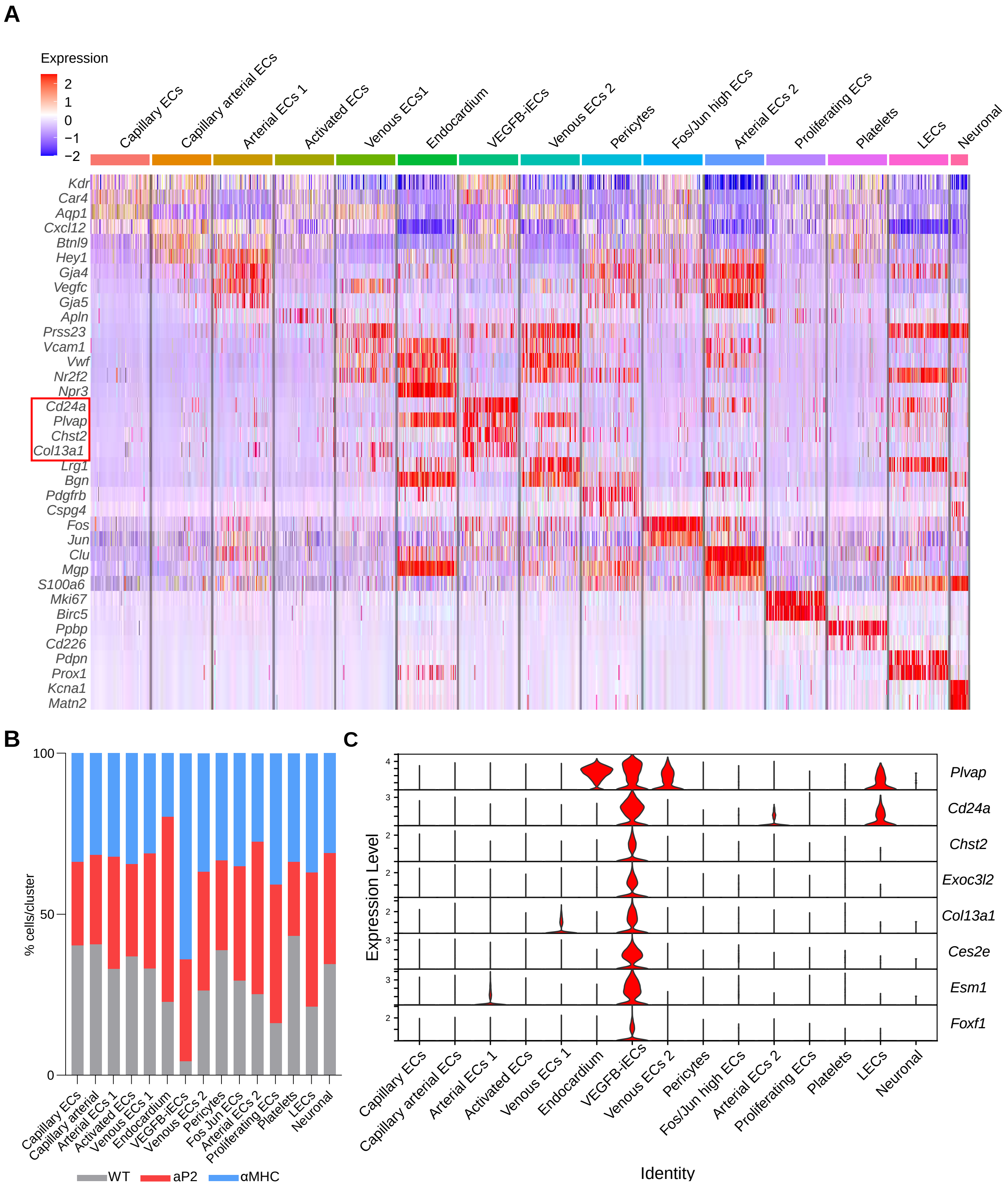

Supplemental figure 4

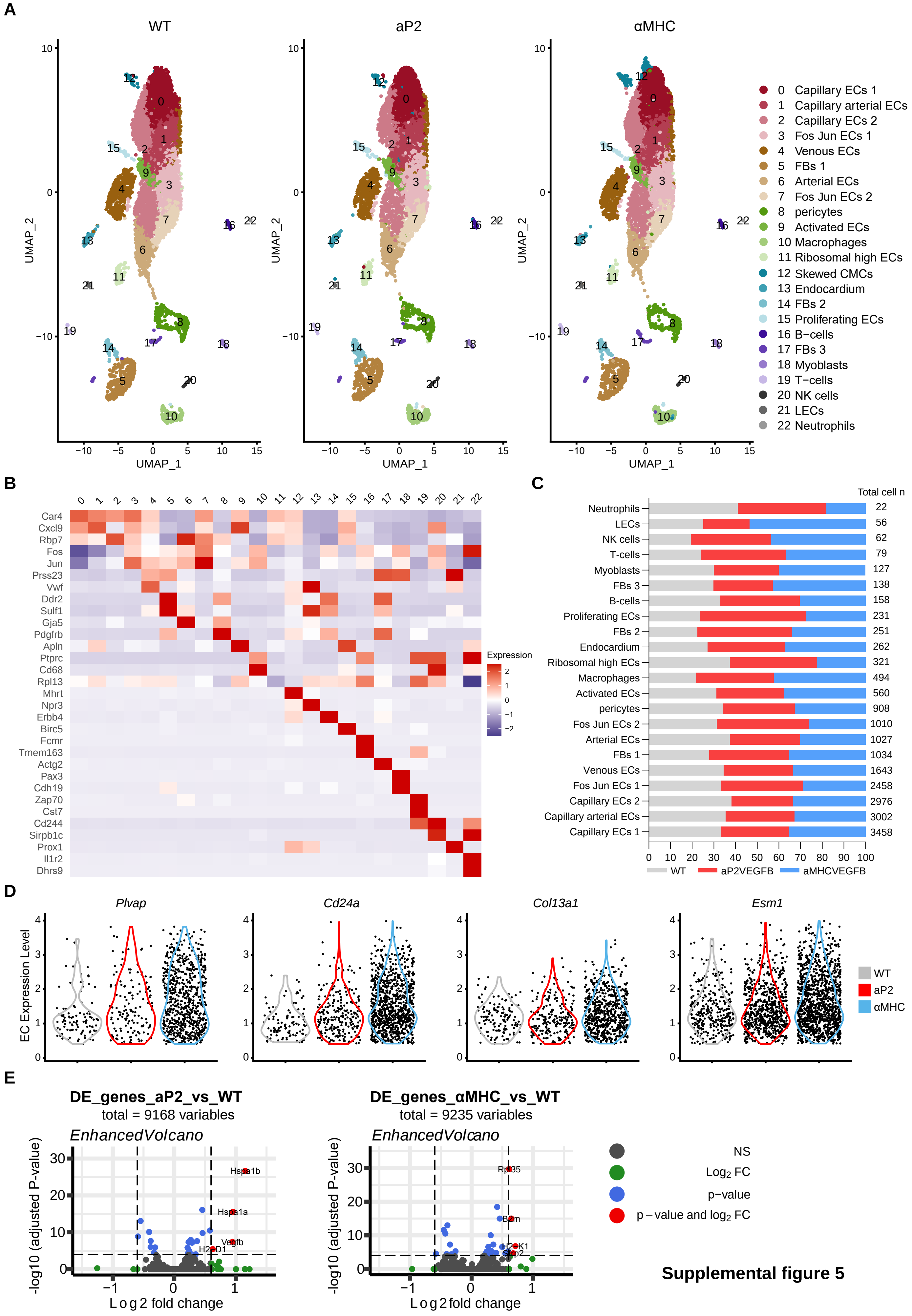

Supplemental figure 5

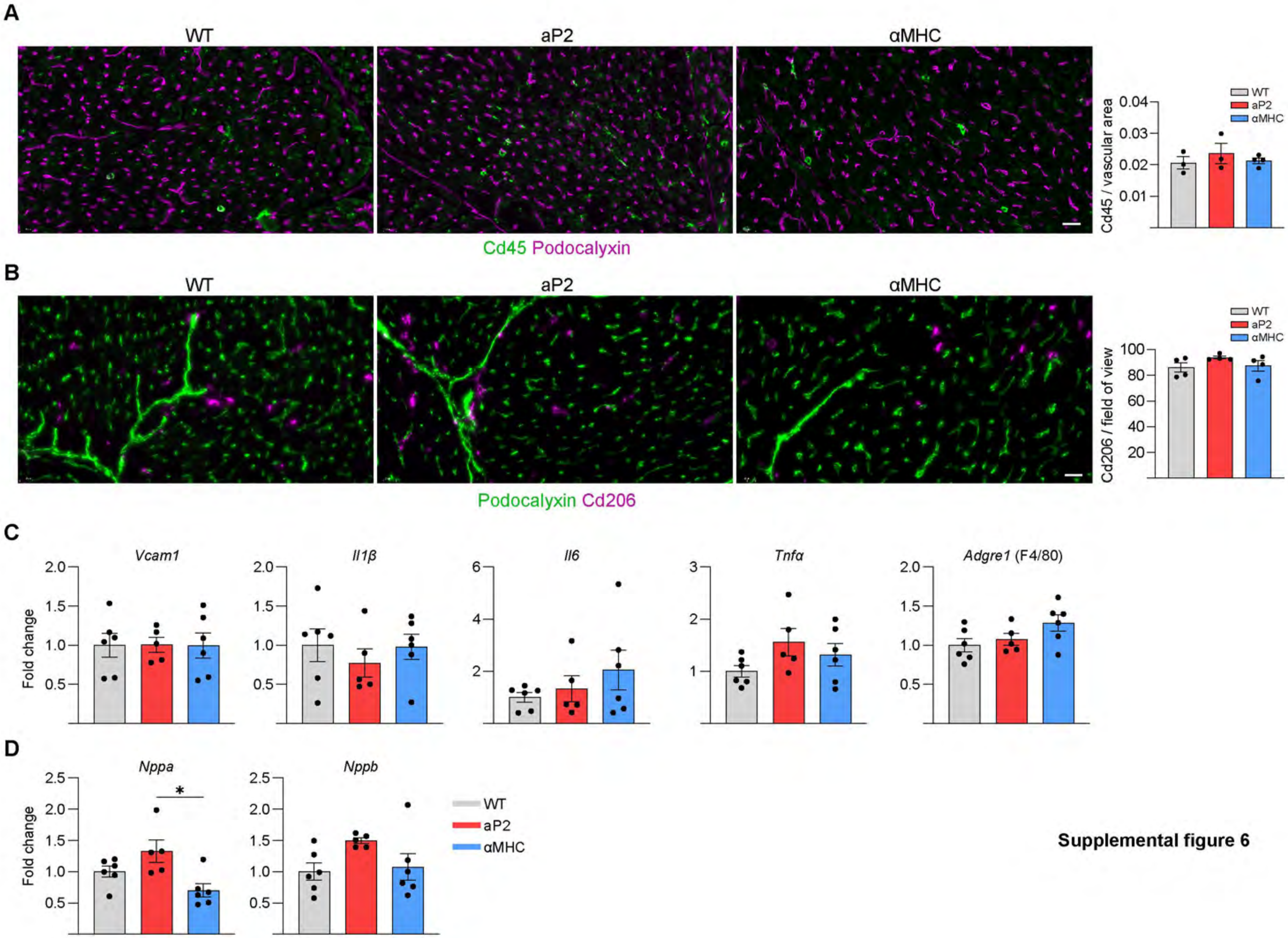

Supplemental figure 6

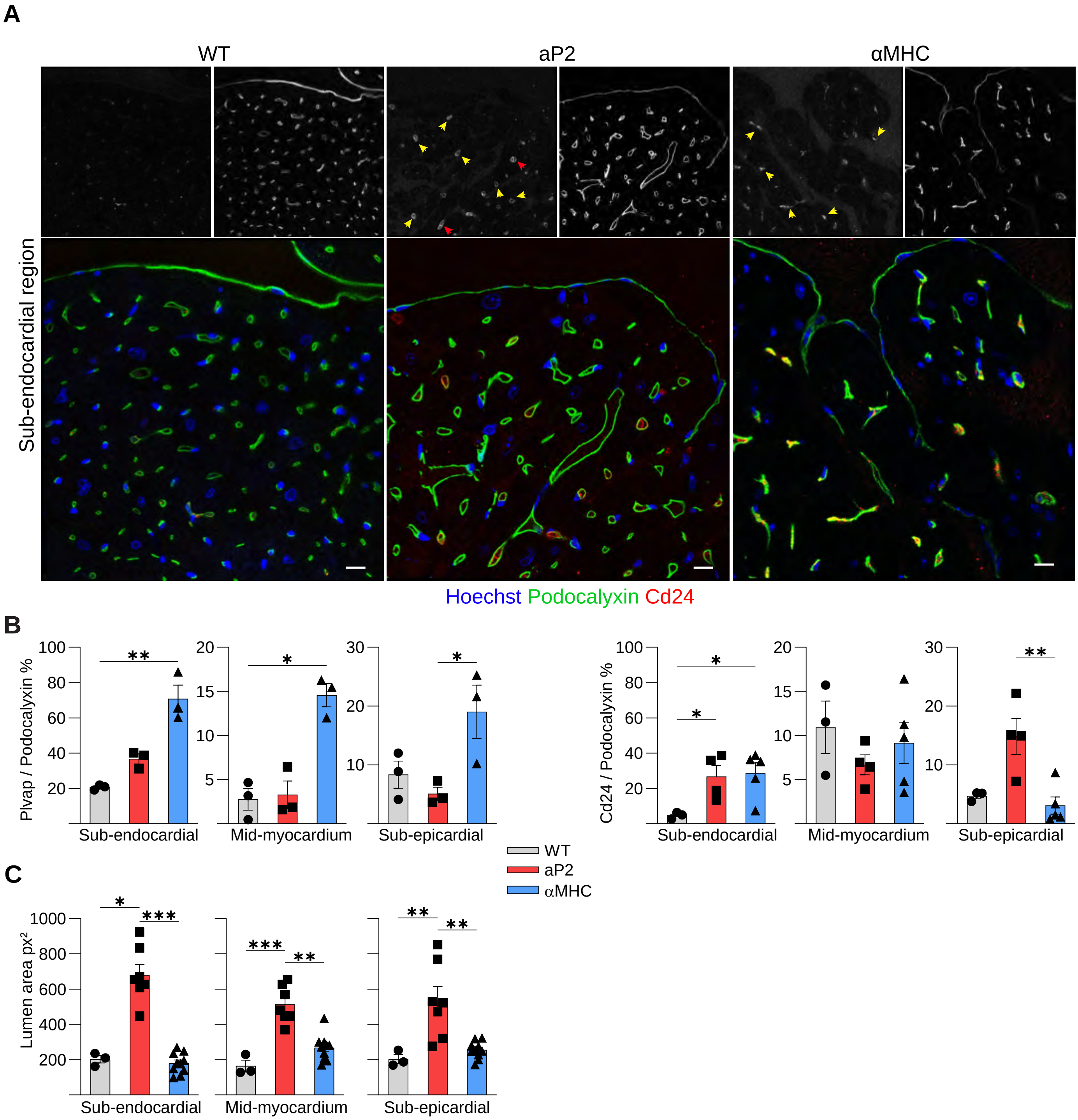

Supplemental figure 7

A

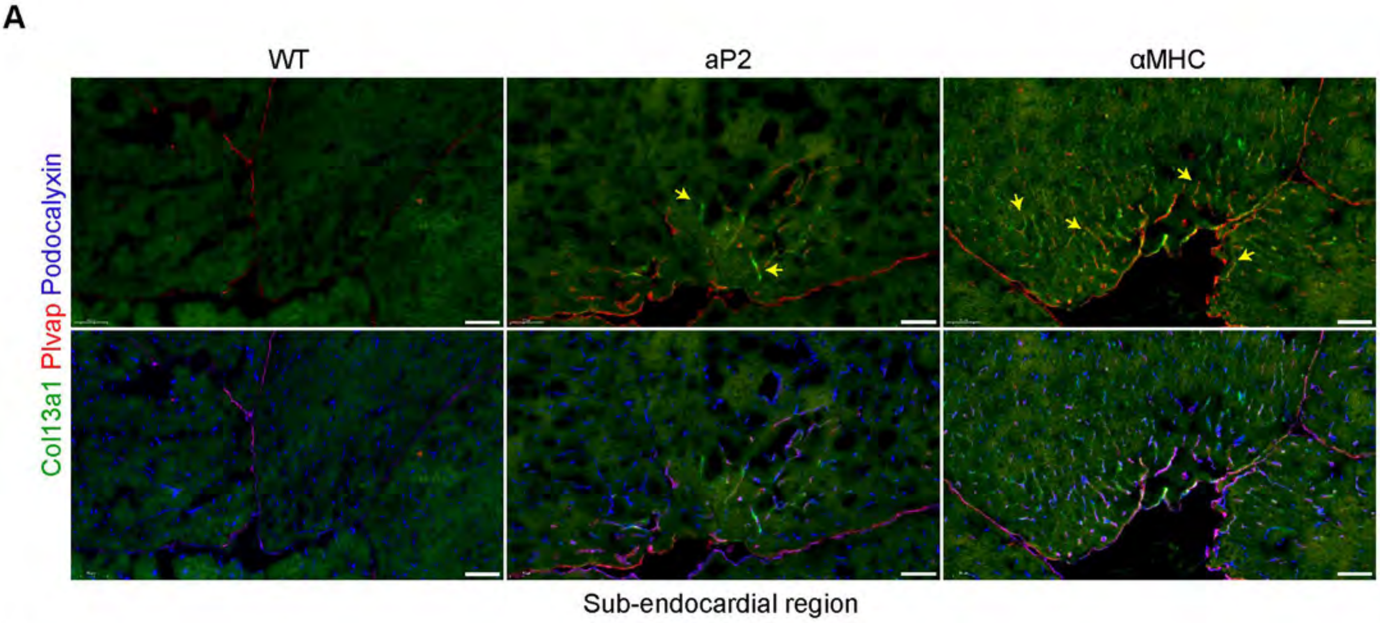

B

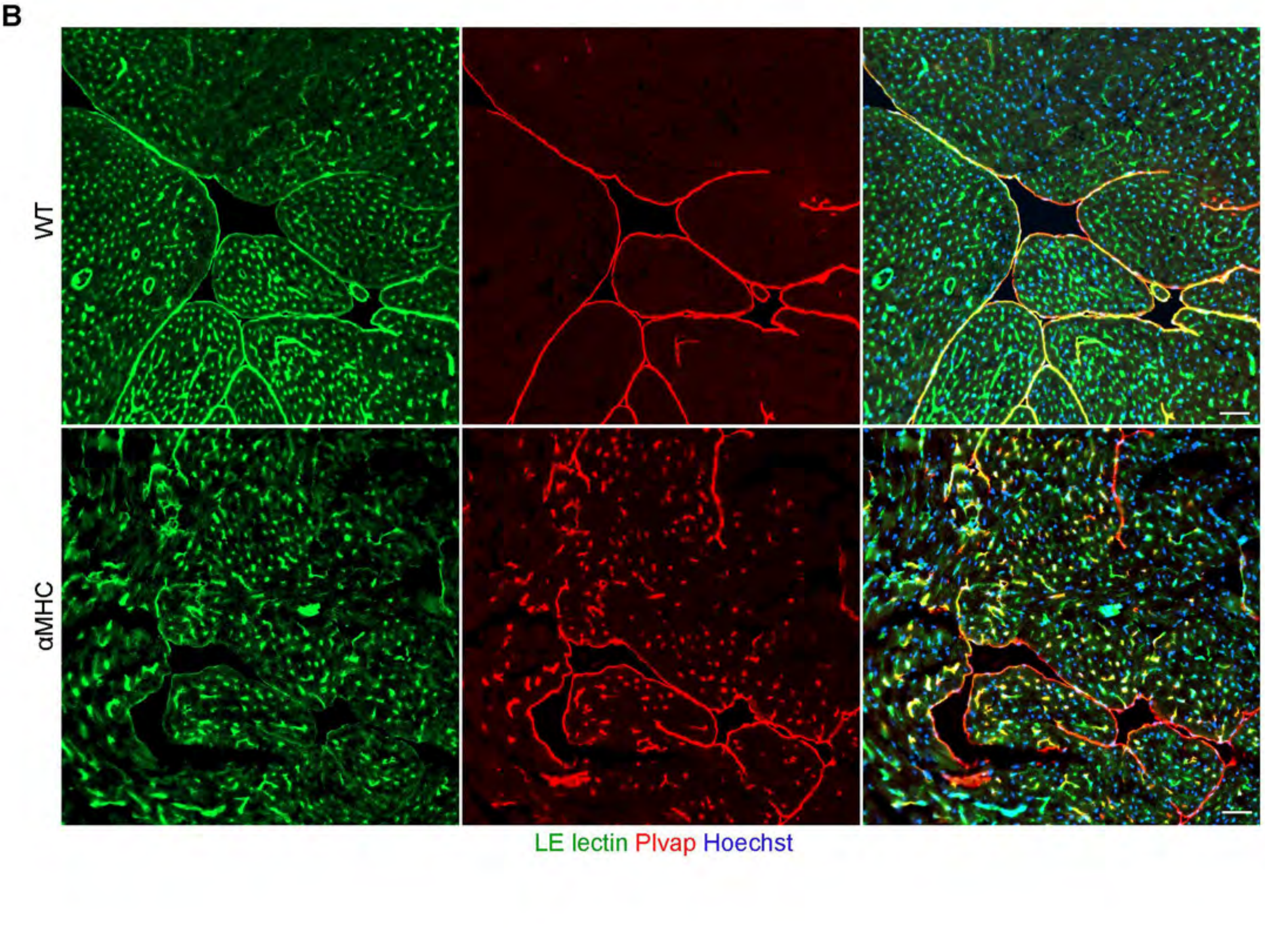

Supplemental figure 8

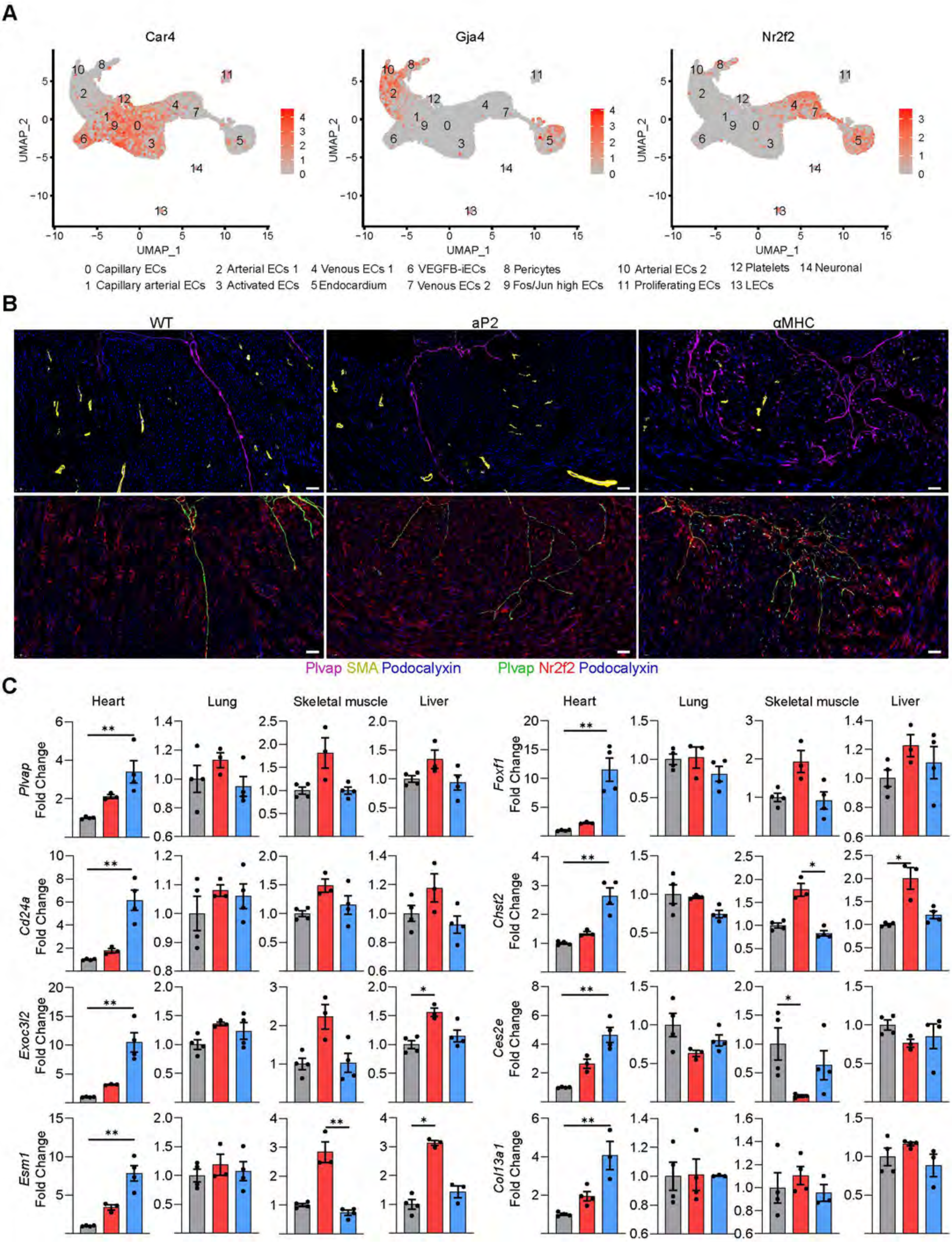

Supplemental figure 9

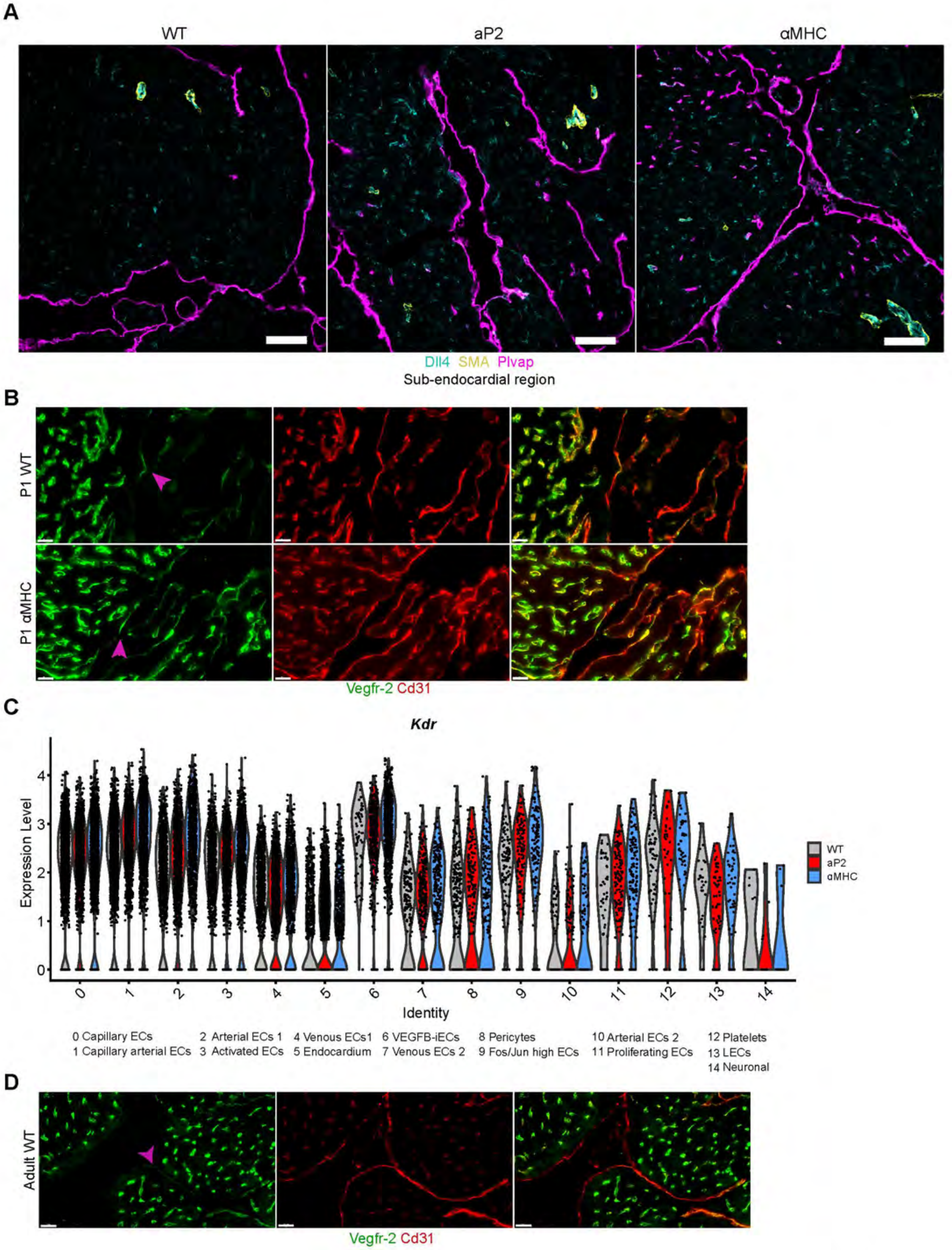

Supplemental figure 10

A

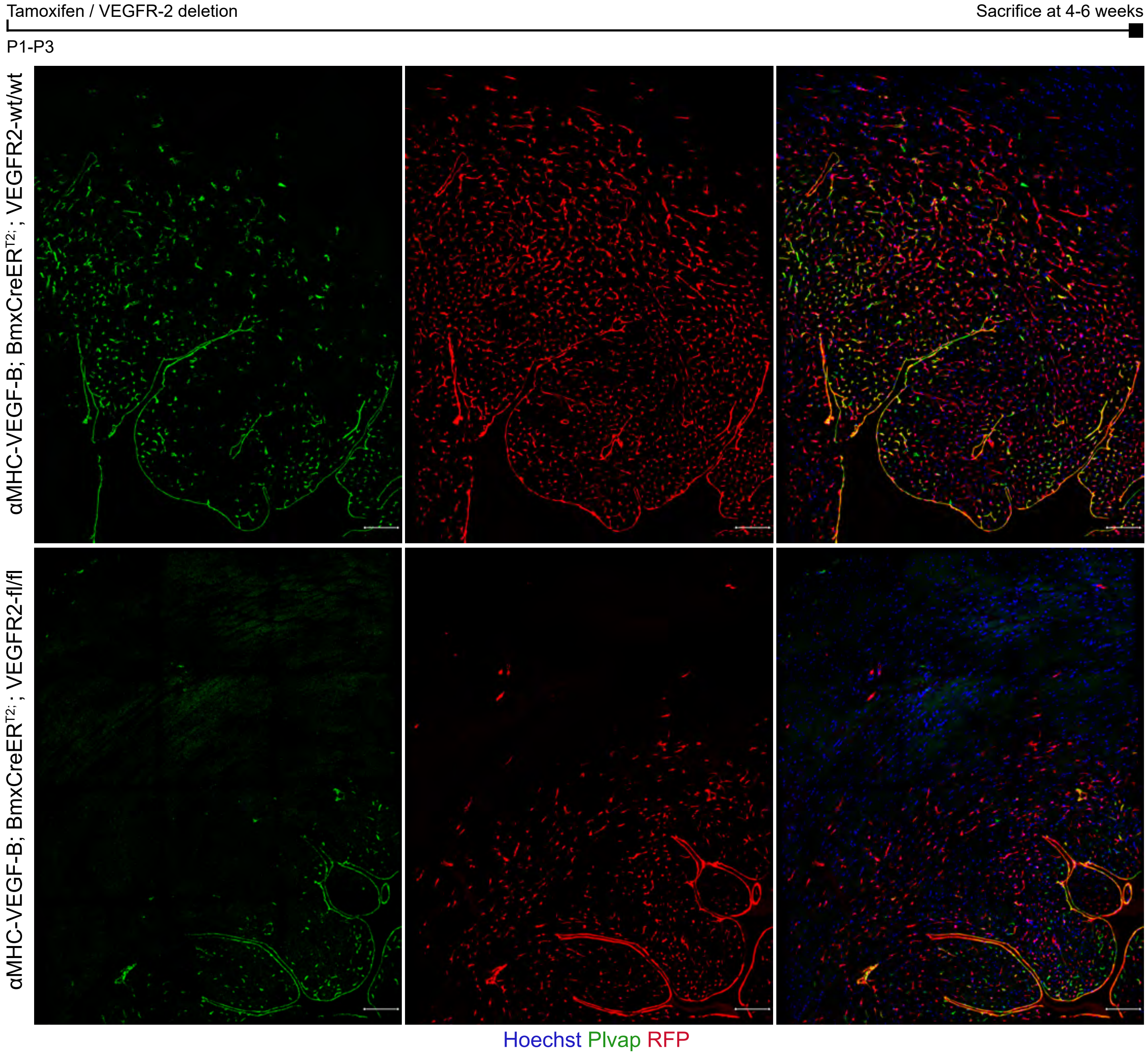

Supplemental figure 11

A

	Operated	Survived	Survival percentage
WT (αMHC-VEGF-B littermates)	8	6	75
αMHC-VEGF-B	8	6	75
WT (aP2-VEGF-B littermates)	5	4	80
aP2-VEGF-B	11	2	18.18

B

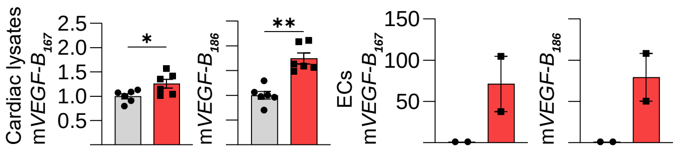

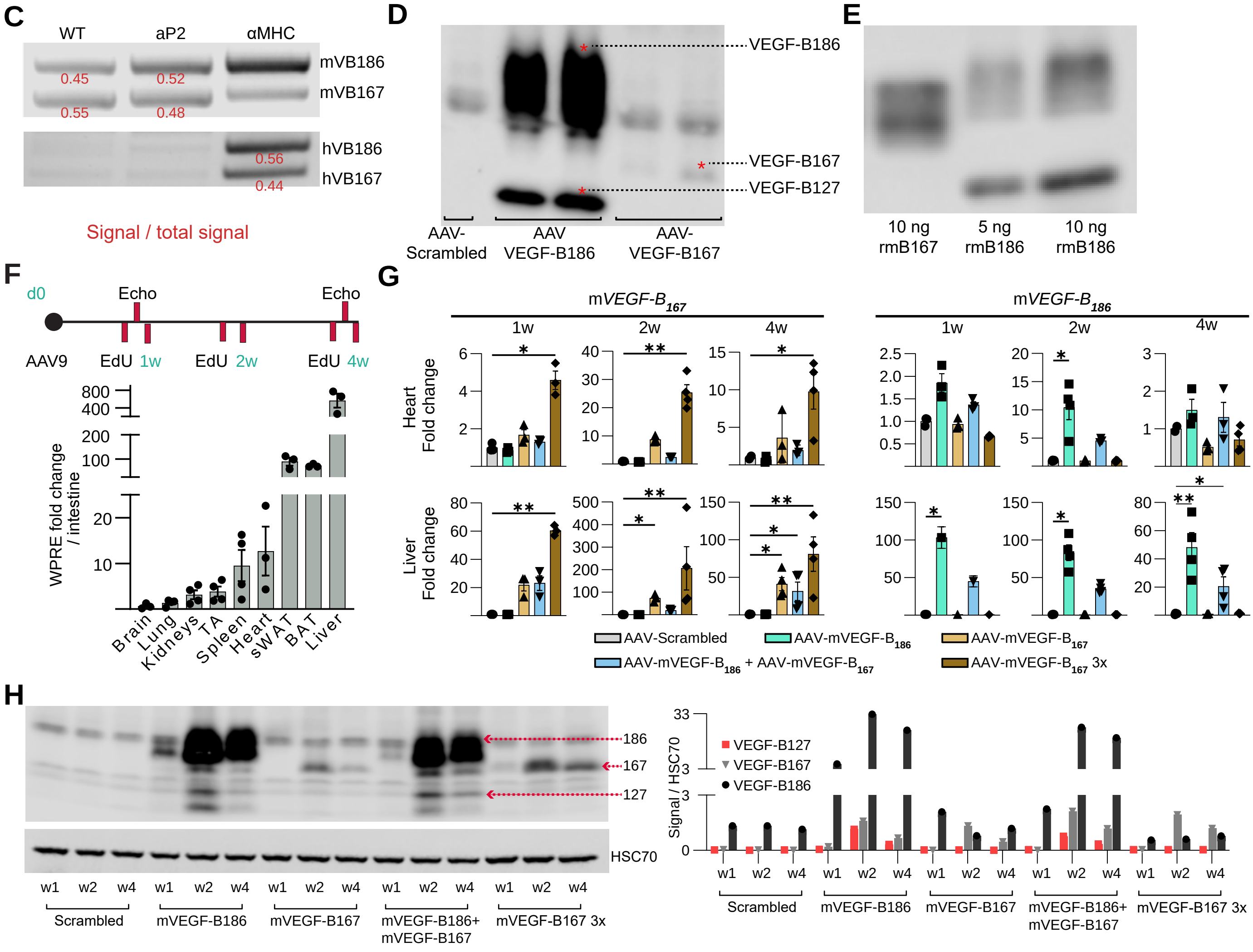

Supplemental figure 12

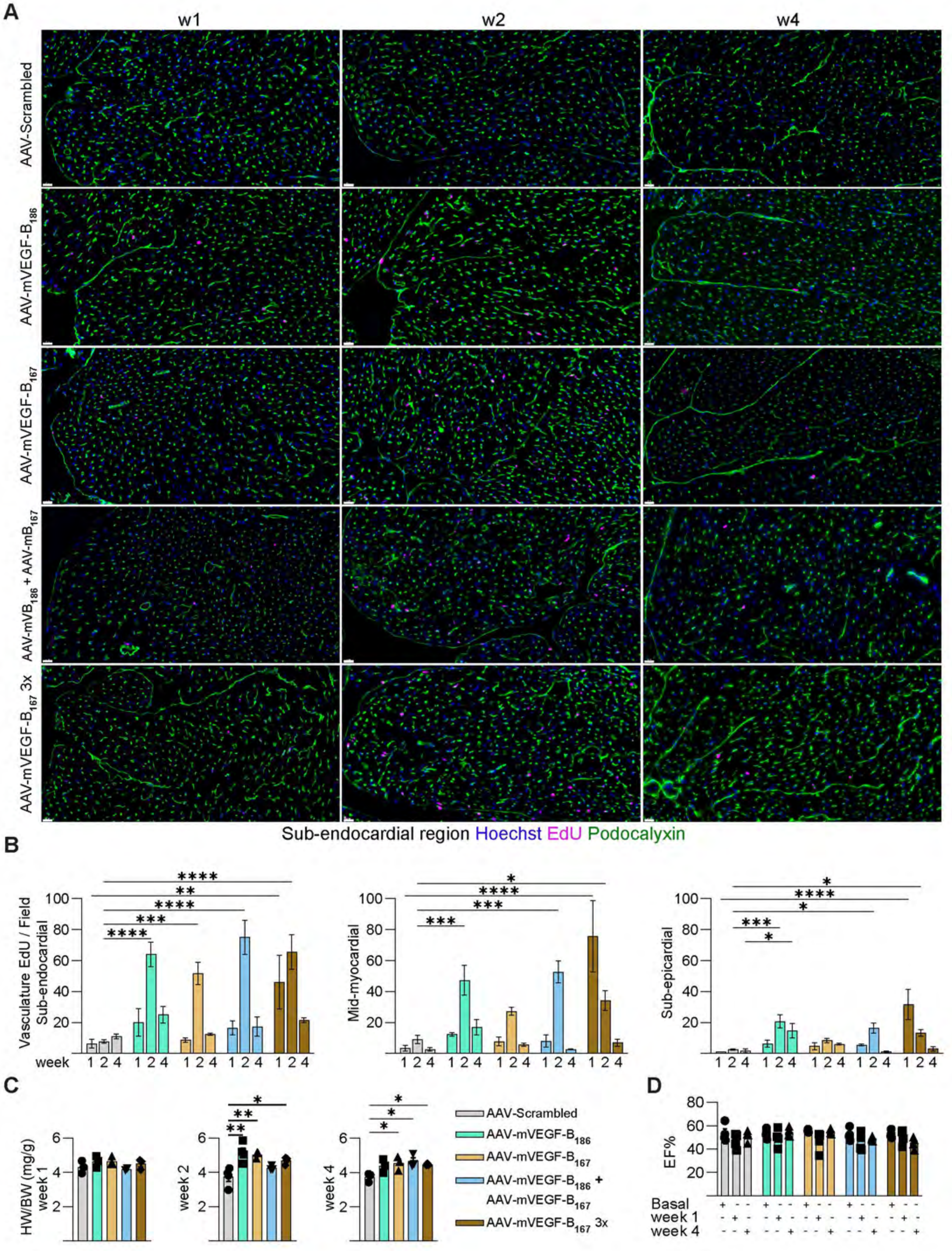

Supplemental figure 13

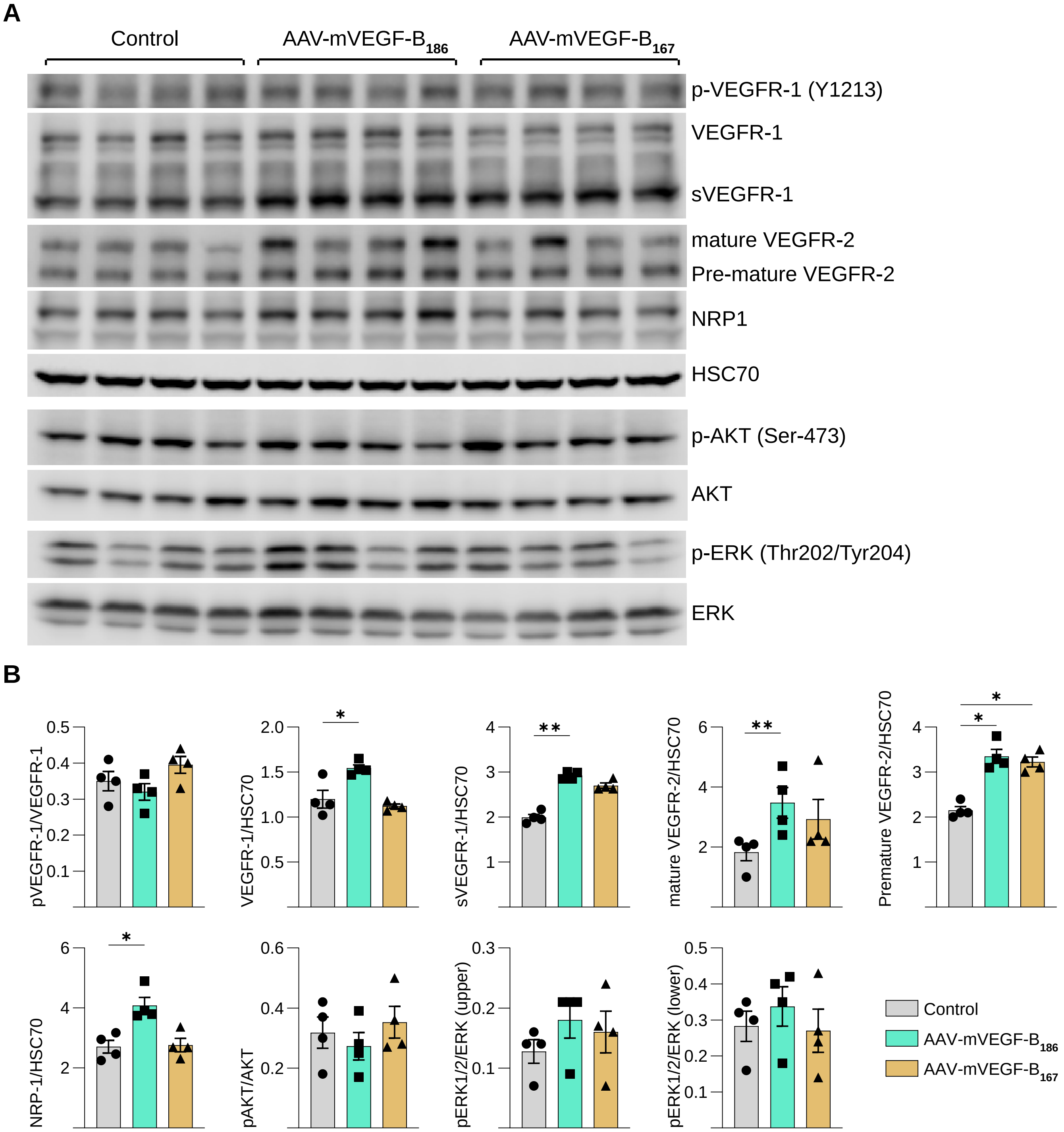

Supplemental figure 14

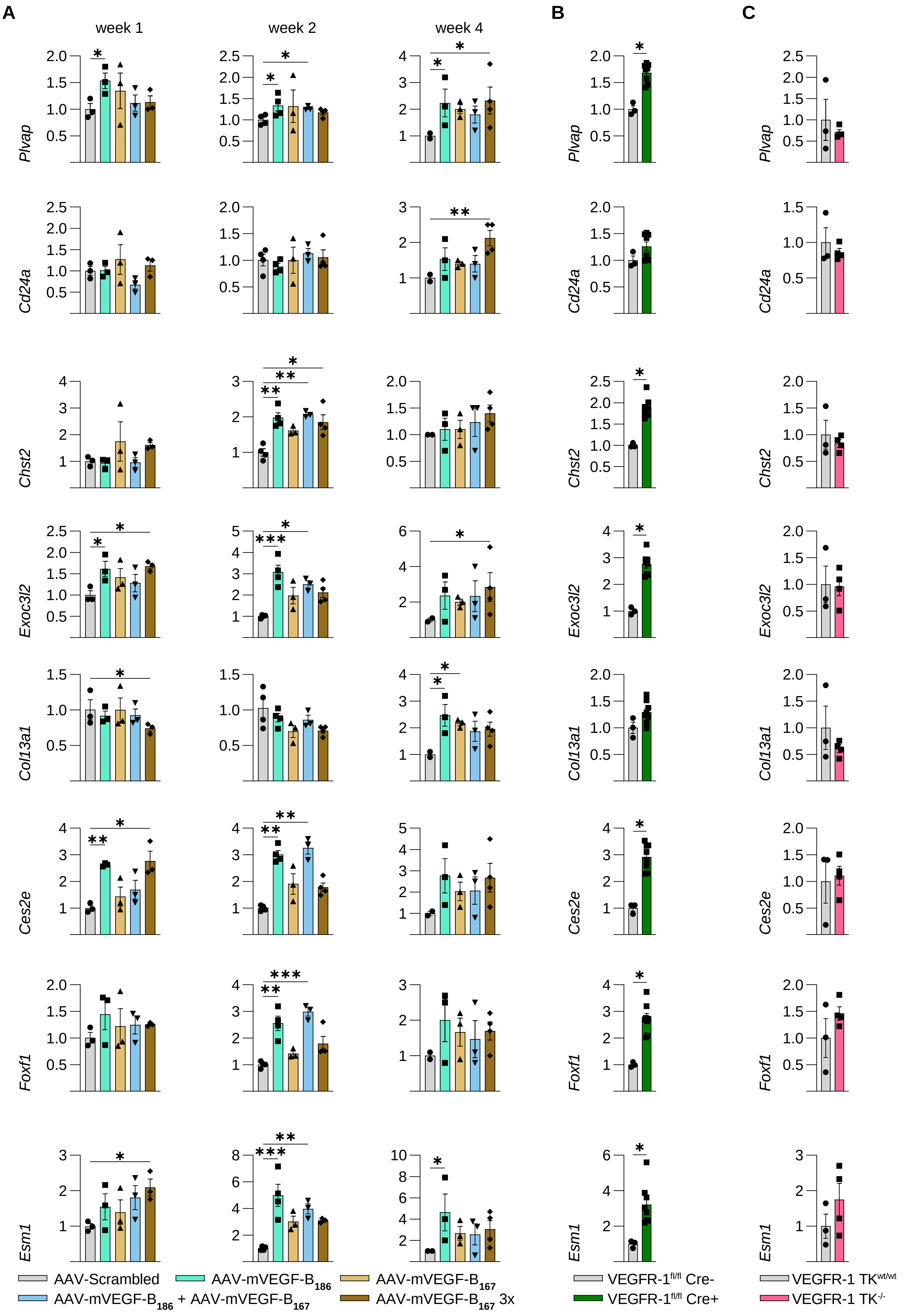

Supplemental figure 15

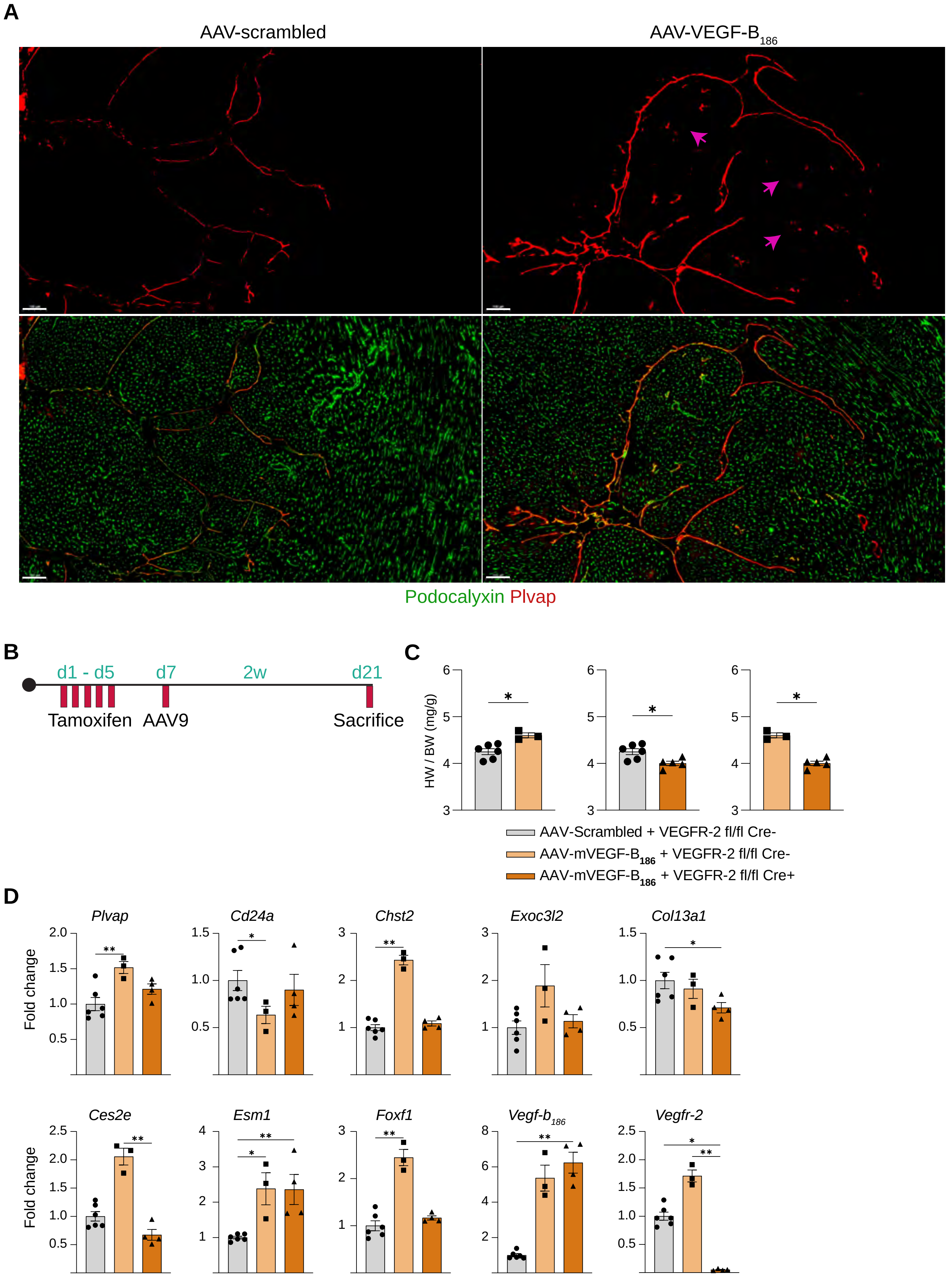

Supplemental figure 16
